# Supplementary material for: Unravelling genomic drivers of speciation in Musa through genome assemblies of wild banana ancestors
Source: Nat Commun. 2025 Jan 23;16:961. doi: 10.1038/s41467-025-56329-4 (PMC11754795; doi:10.1038/s41467-025-56329-4)
Supplement: Supplementary file 1 — Supplementary Information [file 41467_2025_56329_MOESM1_ESM.pdf]

Unravelling genomic drivers of speciation in *Musa* through genome assemblies of wild banana ancestors

Martin *et al.*

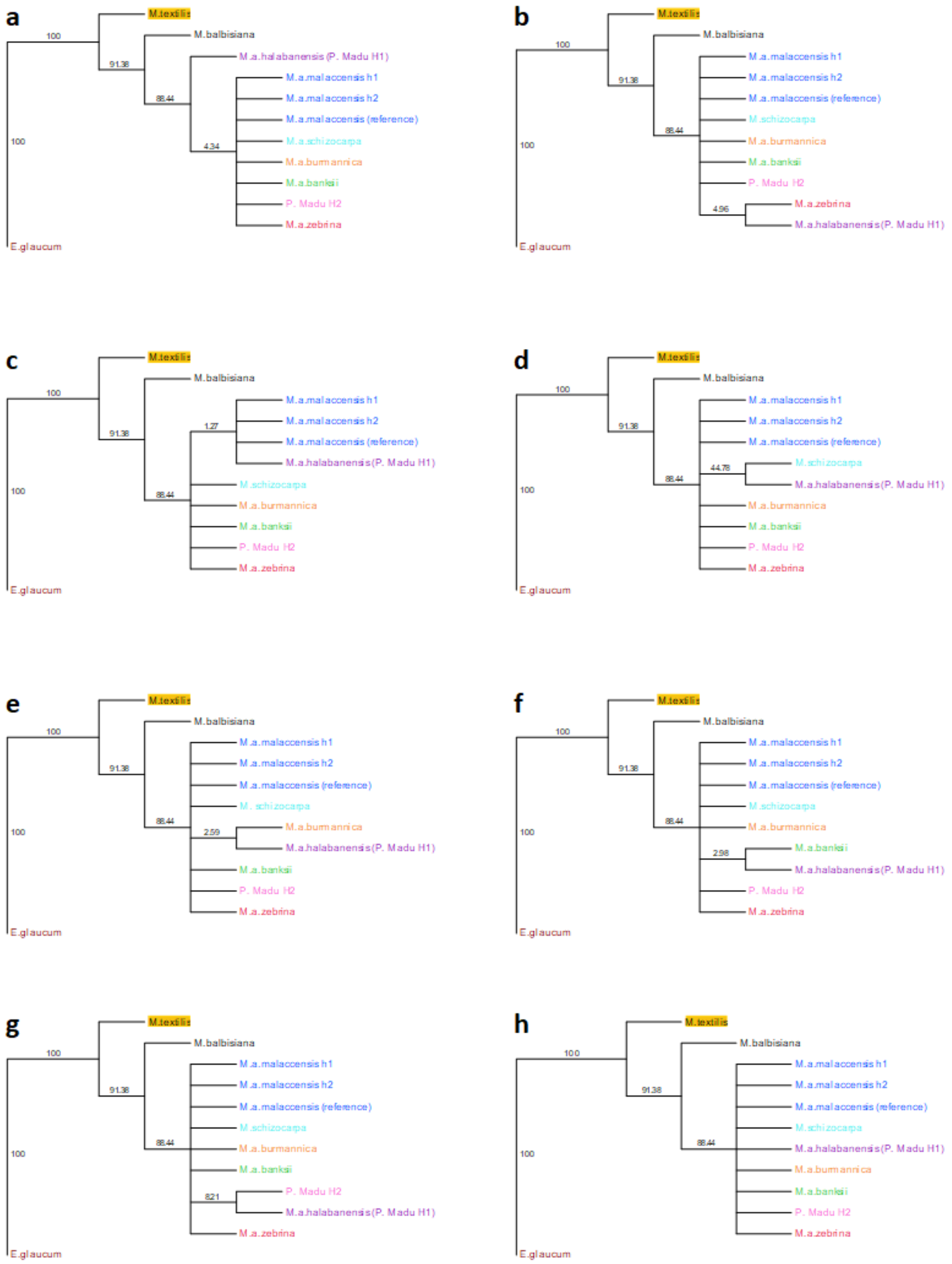

Supplementary Fig. 1. Branch support of alternative position of *M. a. halabanensis* assembly in 10,635 gene pair phylogenies.

From a to h: *M. a. halabanensis* alternative position in gene phylogenies. *M. schizocarpa* and *M.a. halabanensis* formed a monophyletic group (supported by 45% of gene phylogenies) that is at the basis of the monophyletic group formed by all other *M. acuminata* ssp. and the unknown ancestor (supported by 18% of gene phylogenies with alternative positions being each supported by less than 10% gene phylogenies). Source data are provided as a Source Data file.

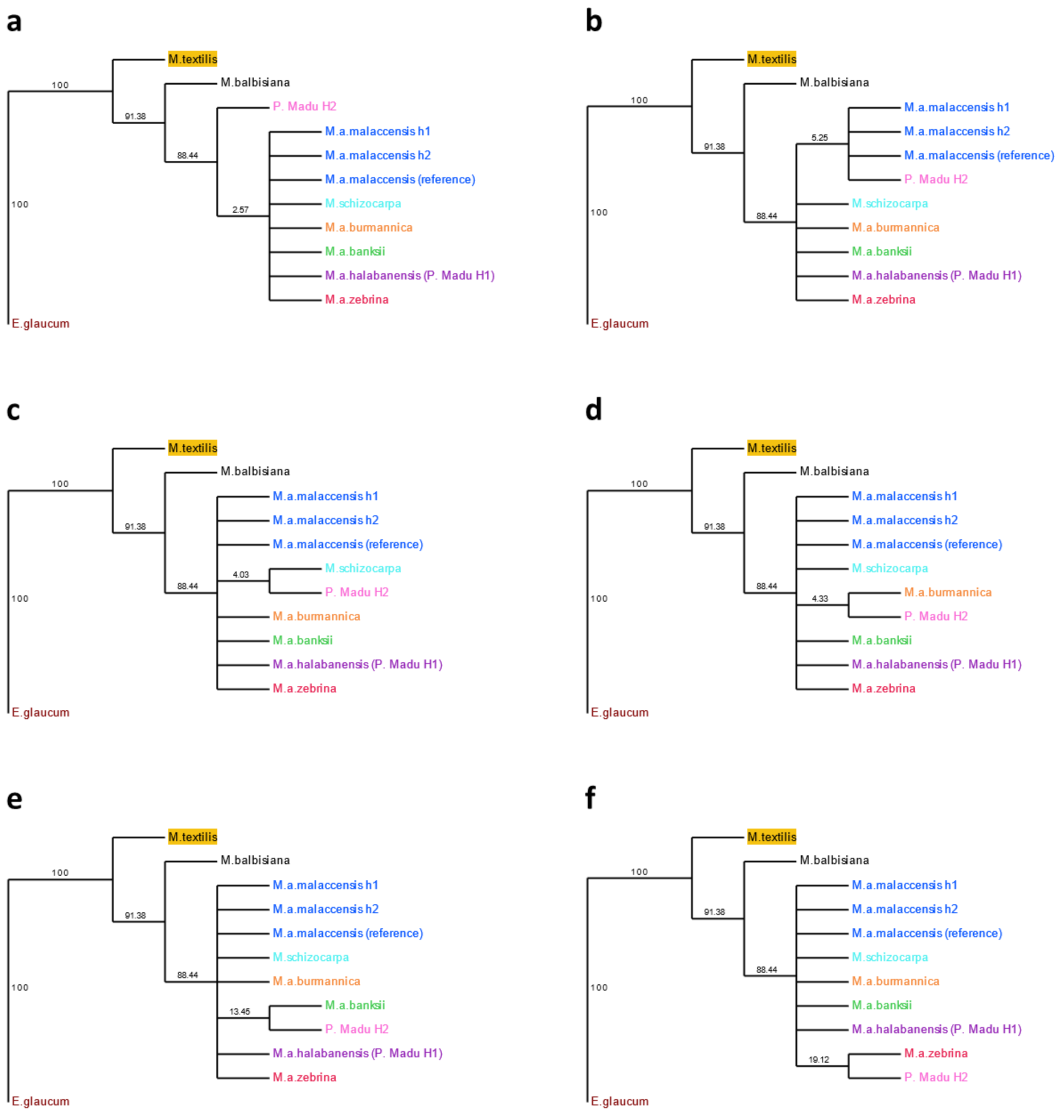

Supplementary Fig. 2. Branch support of alternative position of Pisang Madu H2 assembly in 10,635 gene pair phylogenies.

From a to f: Unknown ancestor Pisang Madu H2 alternative position in gene phylogenies. The unknown ancestor formed a monophyletic group with *M. a. zebrina* (supported by 19% of gene phylogenies with alternative positions being each supported by less than 13% gene phylogenies). Note that all assemblies of *M. a. malaccensis* formed a monophyletic group supported by 31% of gene phylogenies. Source data are provided as a Source Data file.

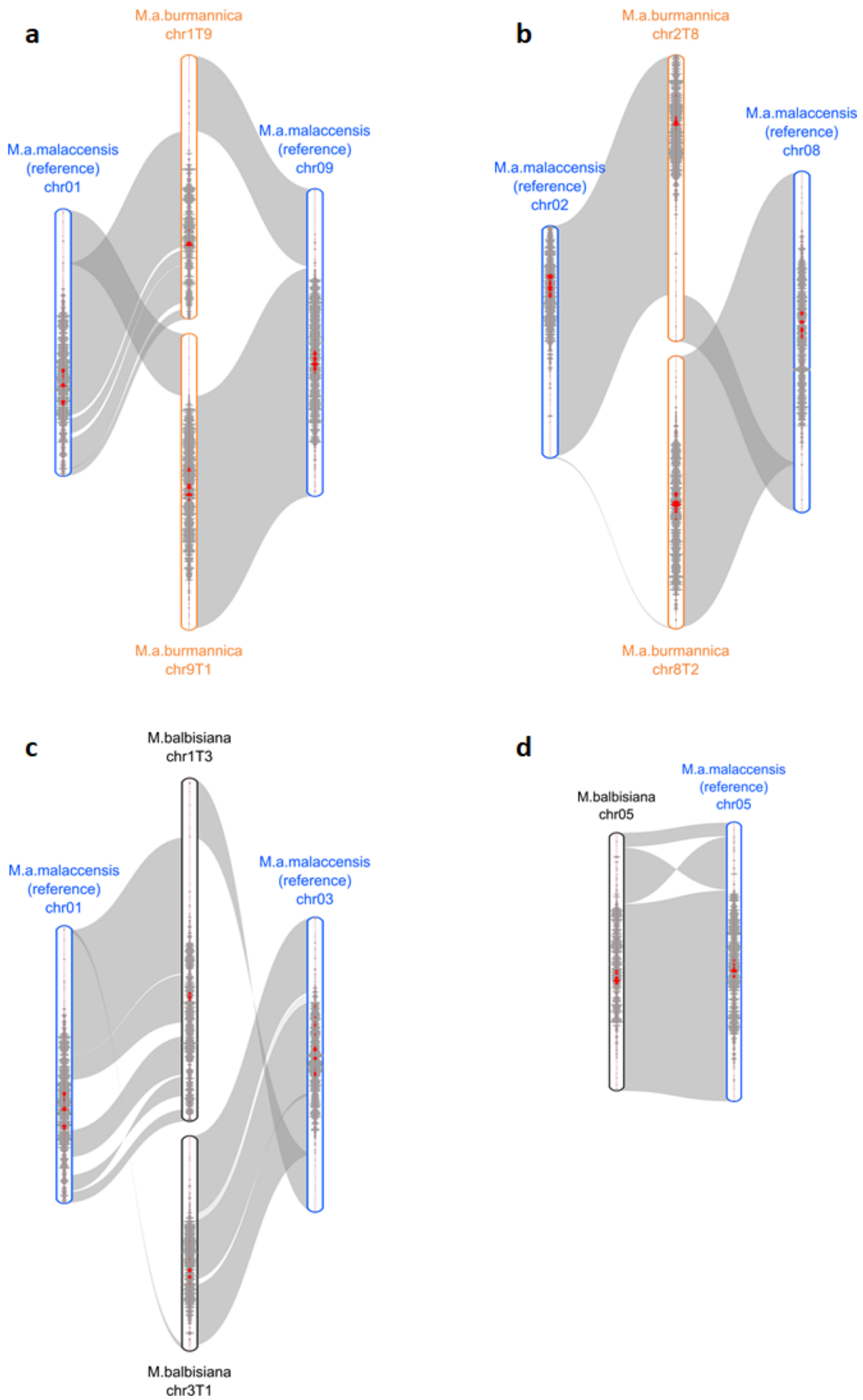

Supplementary Fig. 3. Structure of the 1/4, 3/8 and 1/7 chromosome rearrangements compared to the *M. a. malaccensis* reference structure.

The reference structure corresponds to the *M. a. malaccensis* DH-Pahang (*M. a. malaccensis* (reference)) assembly[1] compared to (a) 1/9 rearranged structure of *M. a. burmannica* assembly; (b) 2/8 rearranged structure of *M. a. burmannica* assembly; (c) 1/3 rearranged structure of *M. balbisiana* assembly[2]; (d) chromosome 05 rearranged structure of *M. balbisiana* assembly[2]. Syntenic regions are indicated with ribbons. Chromosome and translocated fragments were named according to the nomenclature of Martin *et al*.[3]. Curves within the chromosomes correspond to (peri)centromeric repeats in grey with Nanica in red. Source data are provided as a Source Data file.

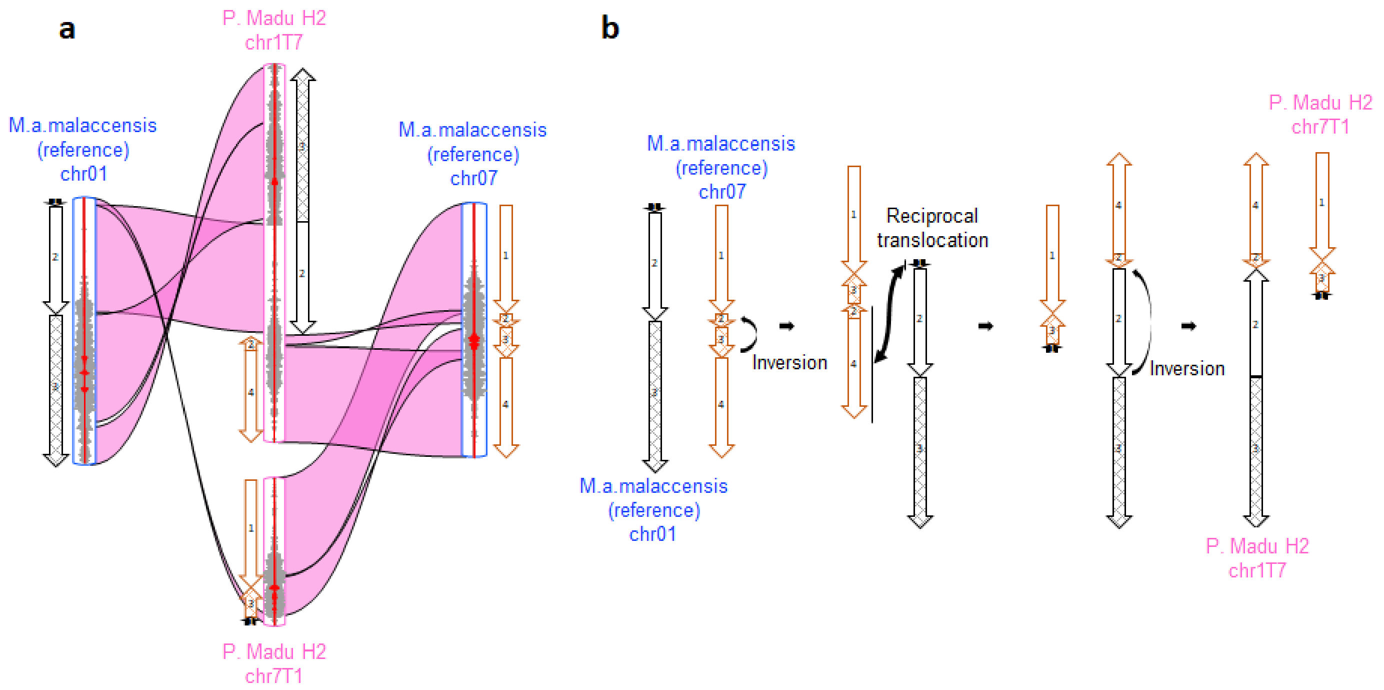

Supplementary Fig. 4. Tentative explanation of structural rearrangement leading to the 1/7 translocation. The reference structure corresponds to the *M. a. malaccensis* DH-Pahang (*M. a. malaccensis* (reference)) assembly[1] compared to 1/7 rearranged structure of Pisang Madu H2 assembly (a). Syntenic regions are indicated with ribbons. Chromosome and translocated fragments were named according to the nomenclature of Martin *et al*.[3]. Curves within the chromosomes correspond to (peri)centromeric repeats in grey with Nanica in red. (b) Structural events that can explain the structure found in Pisang Madu H2 haplotype. Numbered arrows locate rearranged segments and their orientation. Source data are provided as a Source Data file.

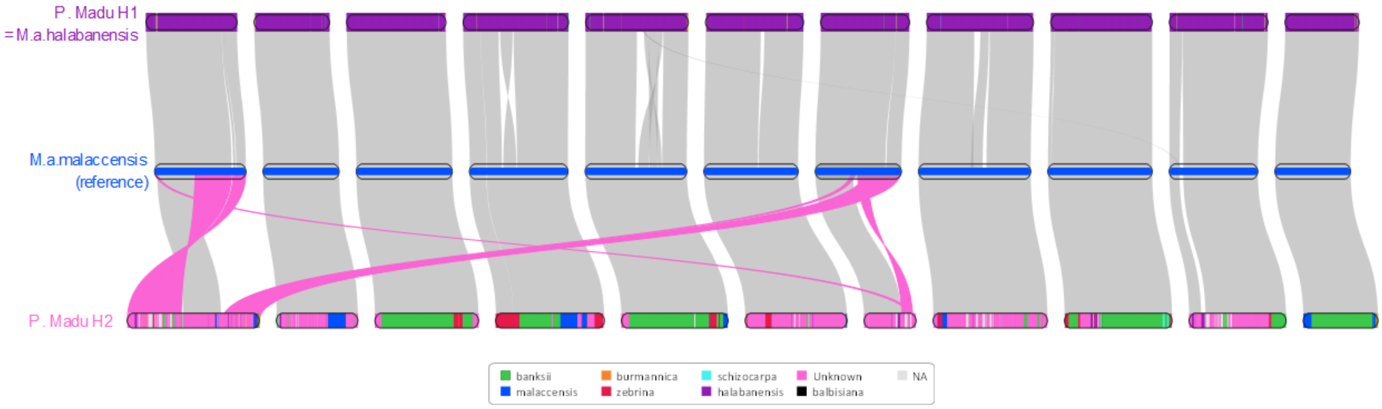

Supplementary Fig. 5. Synteny comparison of Pisang Madu haplotype assemblies with *M. a. malaccensis* reference assembly.
Syntenic regions are indicated with ribbons. Source data are provided as a Source Data file.

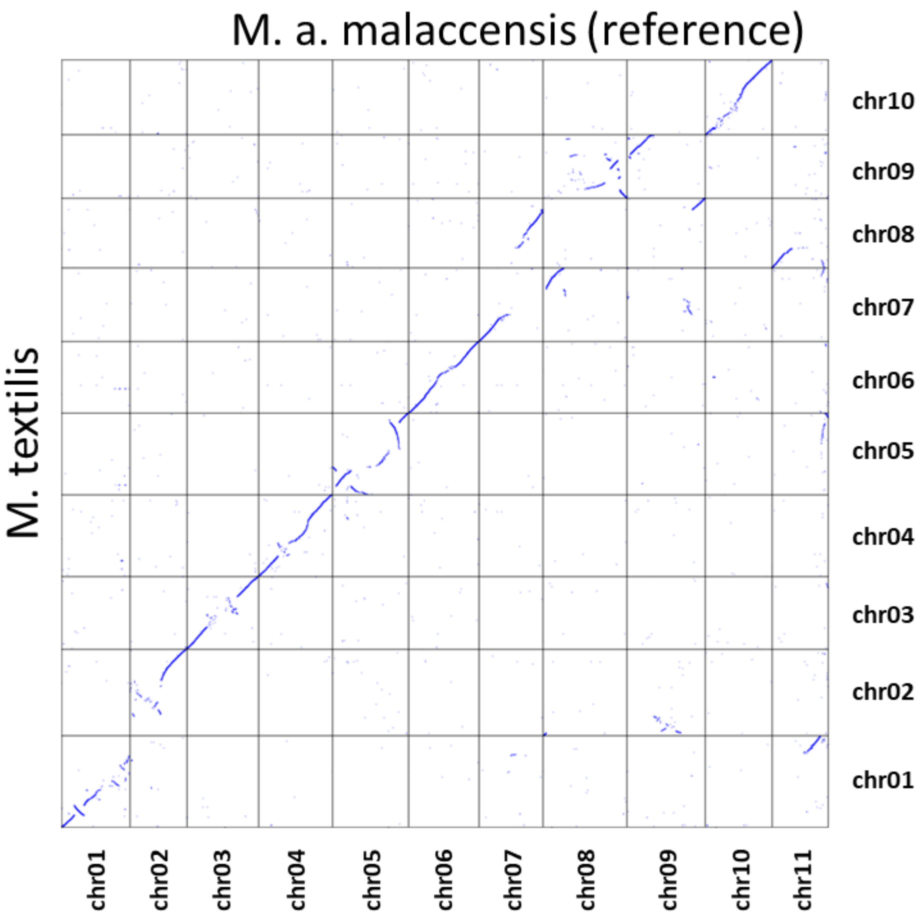

Supplementary Fig. 6. Synteny comparison of the *M. textilis* assembly with the *M. a. malaccensis* reference. *M. textilis* assembly was compared with other assembly using a dot plot approach. Source data are provided as a Source Data file.

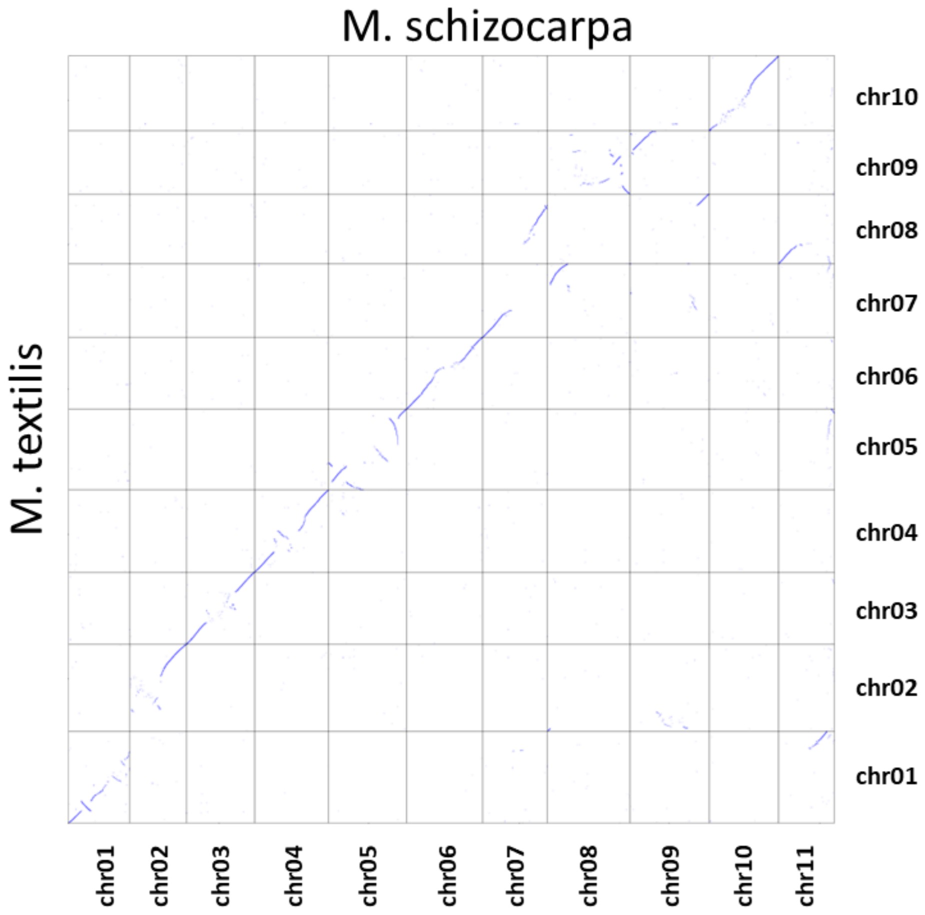

Supplementary Fig. 7. Synteny comparison of the *M. textilis* assembly with the *M. schizocarpa* assembly. *M. textilis* assembly was compared with the other assembly using a dot plot. Source data are provided as a Source Data file.

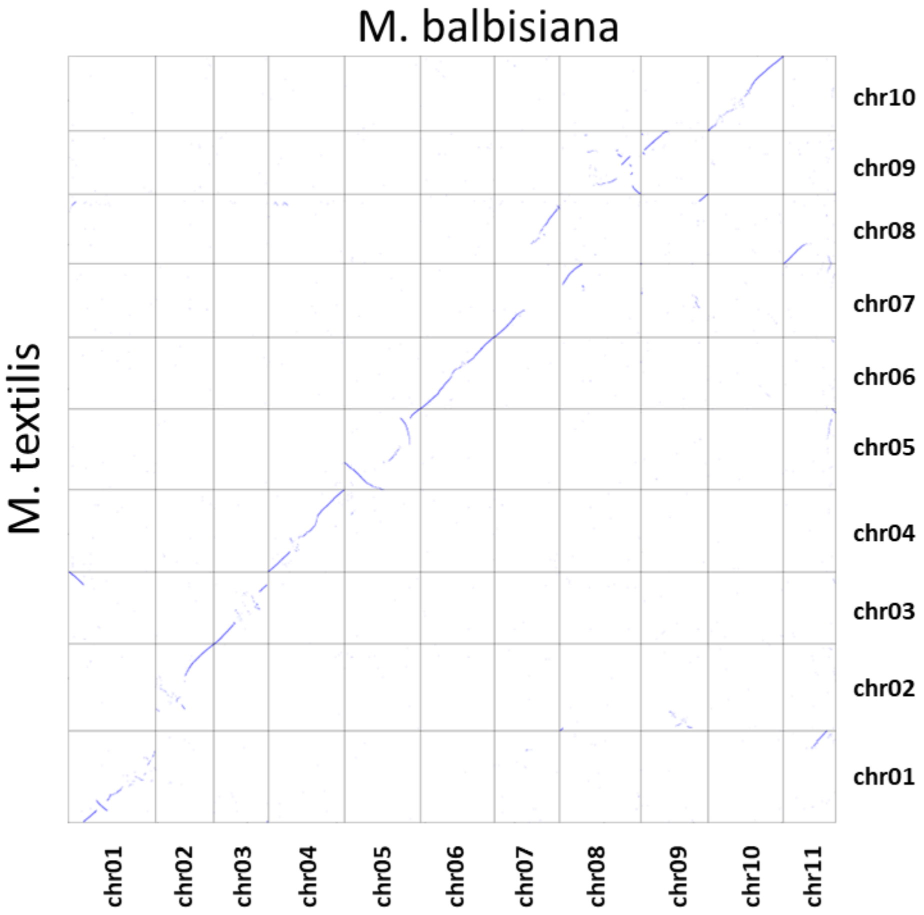

Supplementary Fig. 8. Synteny comparison of the *M. textilis* assembly with the *M. balbisiana* assembly. *M. textilis* assembly was compared with the other assembly using a dot plot. Source data are provided as a Source Data file.

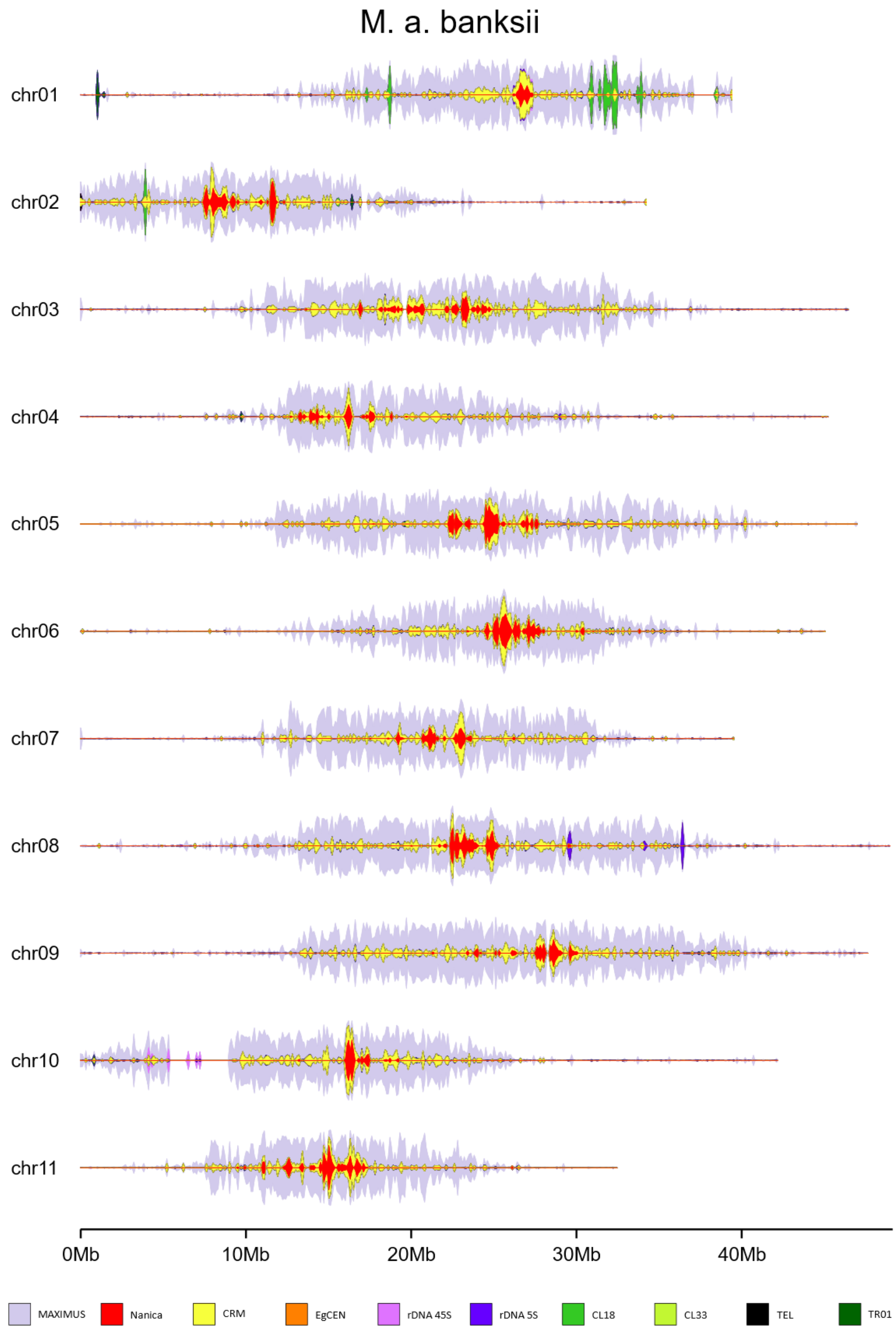

Supplementary Fig. 9. Graphical representation of Centromeric and tandem sequences density on *M. a. banksii* genome assembly.

Stacked curves representing the density of SIRE/Maximus transposable elements, centromeric sequences and tandem repeats along the assemblies. Chromosomes are indicated on the left with the genomic scale at the bottom in Mbases. Color codes for repeated sequences classes are indicated. Source data are provided as a Source Data file.

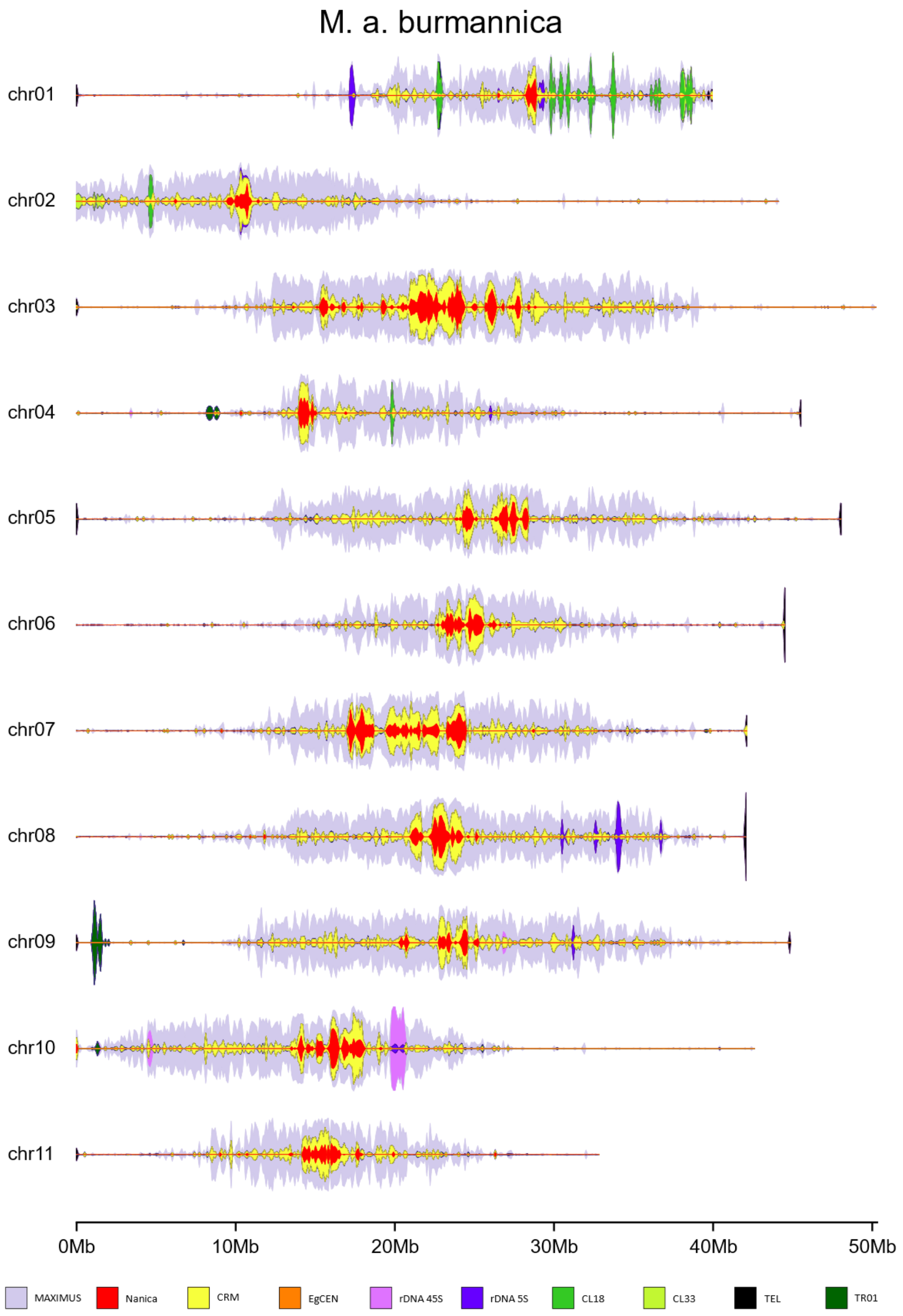

Supplementary Fig. 10. Graphical representation of Centromeric and tandem sequences density on *M. a. burmannica* genome assembly.
Stacked curves representing the density of SIRE/Maximus transposable elements, centromeric sequences and tandem repeats along the assemblies. Chromosomes are indicated on the left with the genomic scale at the bottom in Mbases. Color codes for repeated sequences classes are indicated. Source data are provided as a Source Data file.

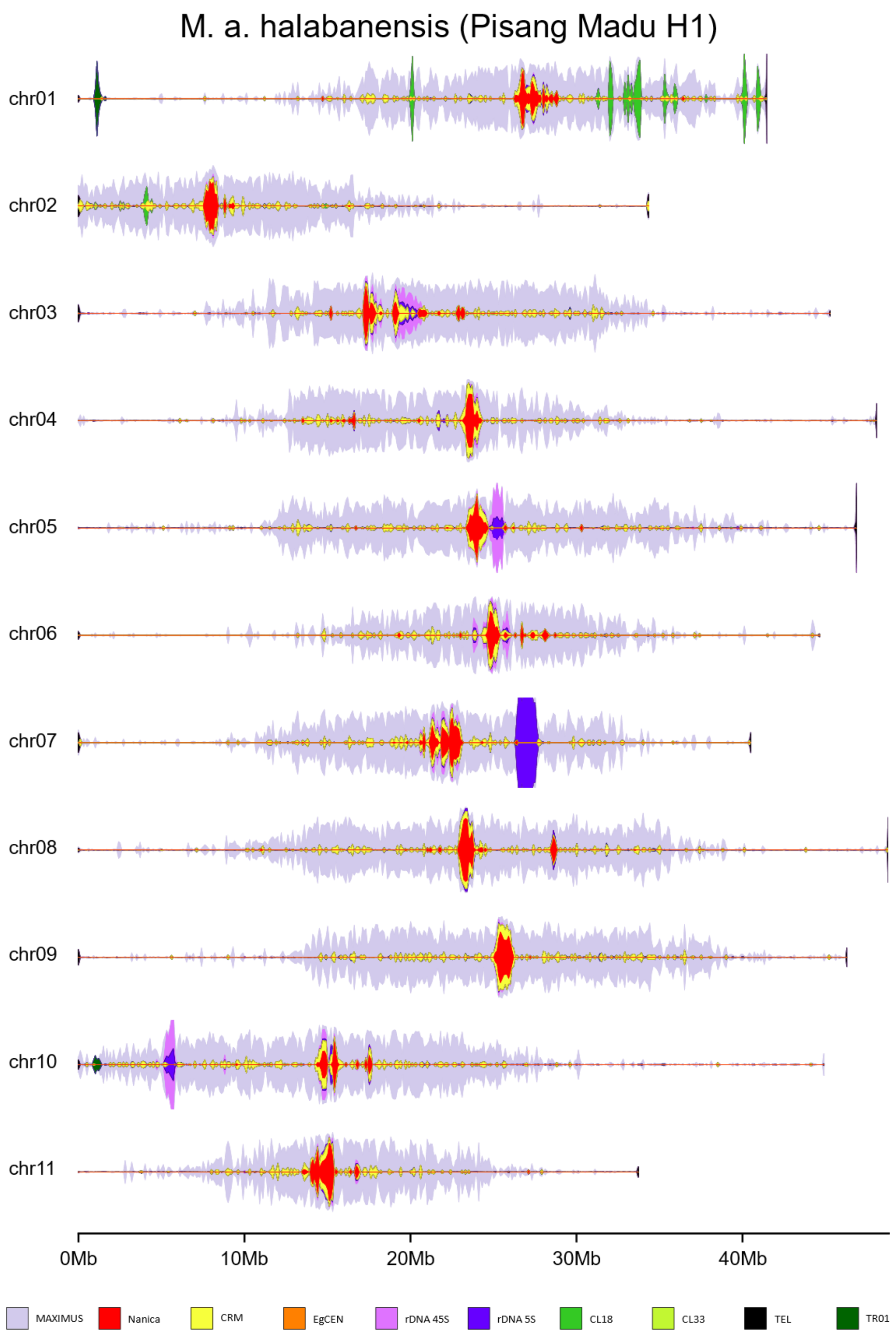

Supplementary Fig. 11. Graphical representation of Centromeric and tandem sequences density on *M. a. halabanensis* (Pisang Madu H1) genome assembly.
Stacked curves representing the density of SIRE/Maximus transposable elements, centromeric sequences and tandem repeats along the assemblies. Chromosomes are indicated on the left with the genomic scale at the bottom in Mbases. Color codes for repeated sequences classes are indicated. Source data are provided as a Source Data file.

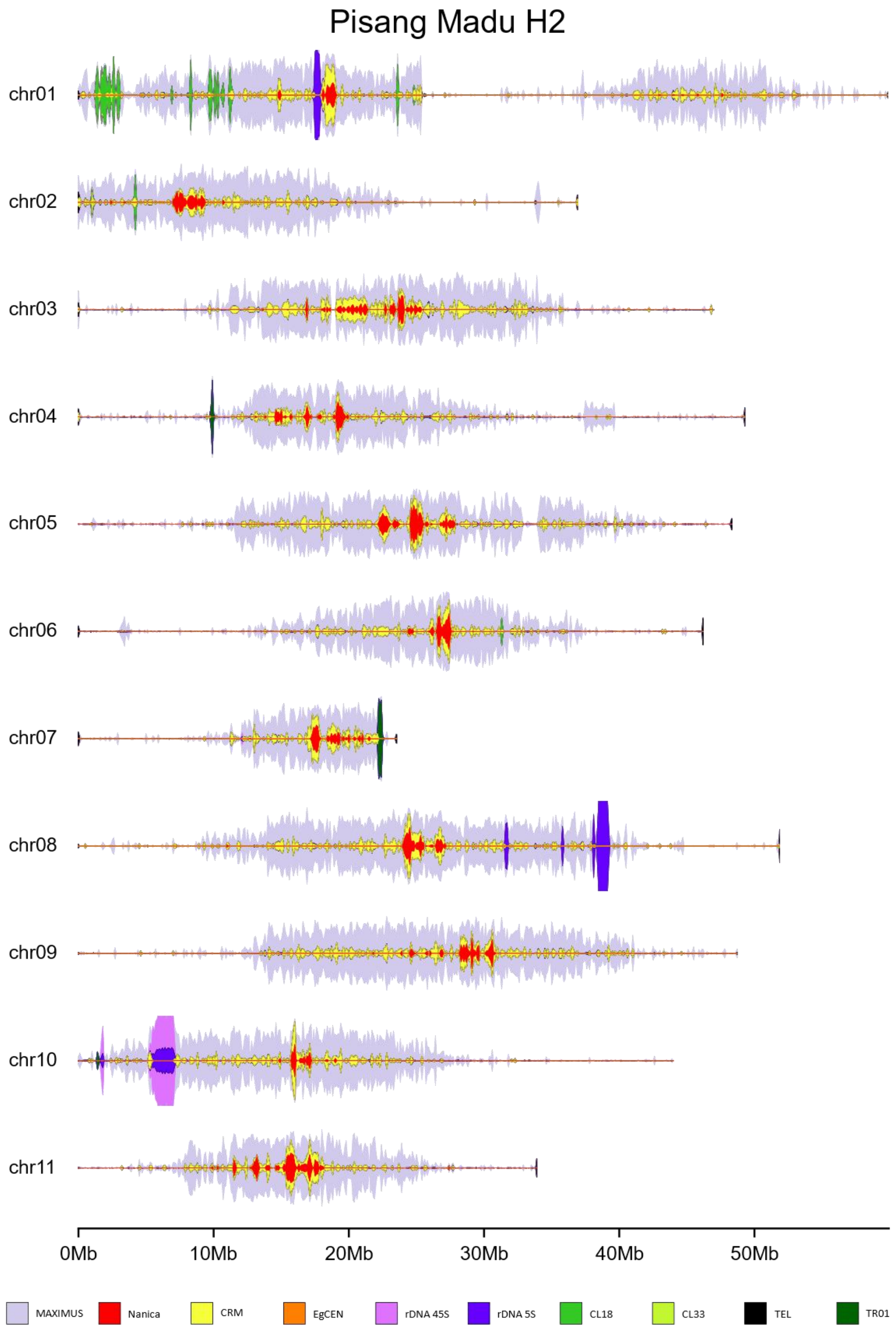

Supplementary Fig. 12. Graphical representation of Centromeric and tandem sequences density on Pisang Madu H2 genome assembly.

Stacked curves representing the density of SIRE/Maximus transposable elements, centromeric sequences and tandem repeats along the assemblies. Chromosomes are indicated on the left with the genomic scale at the bottom in Mbases. Color codes for repeated sequences classes are indicated. Source data are provided as a Source Data file.

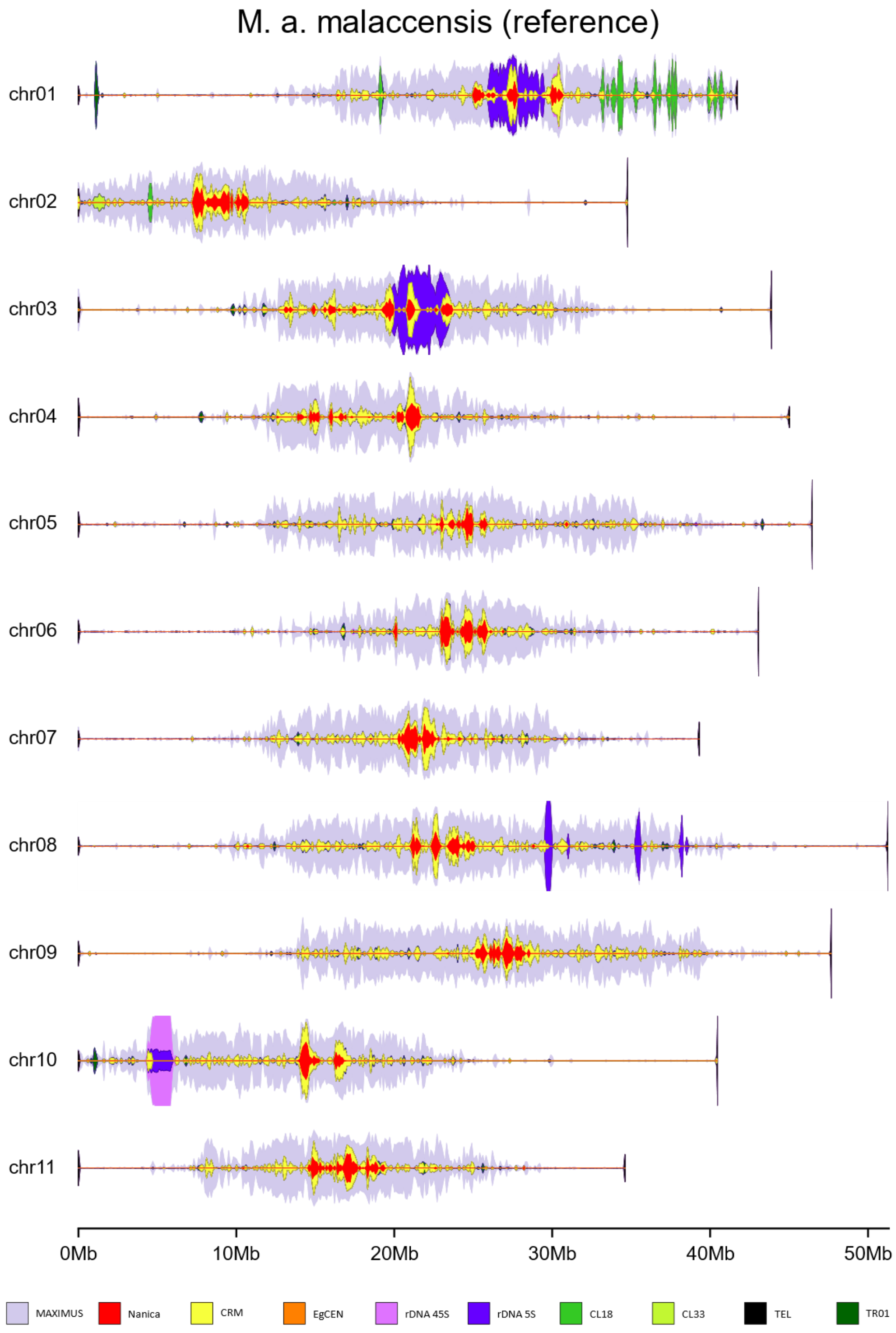

Supplementary Fig. 13. Graphical representation of Centromeric and tandem sequences density on *M. a. malaccensis* reference genome assemblies.

Stacked curves representing the density of SIRE/Maximus transposable elements, centromeric sequences and tandem repeats along the assemblies. Chromosomes are indicated on the left with the genomic scale at the bottom in Mbases. Color codes for repeated sequences classes are indicated. Source data are provided as a Source Data file.

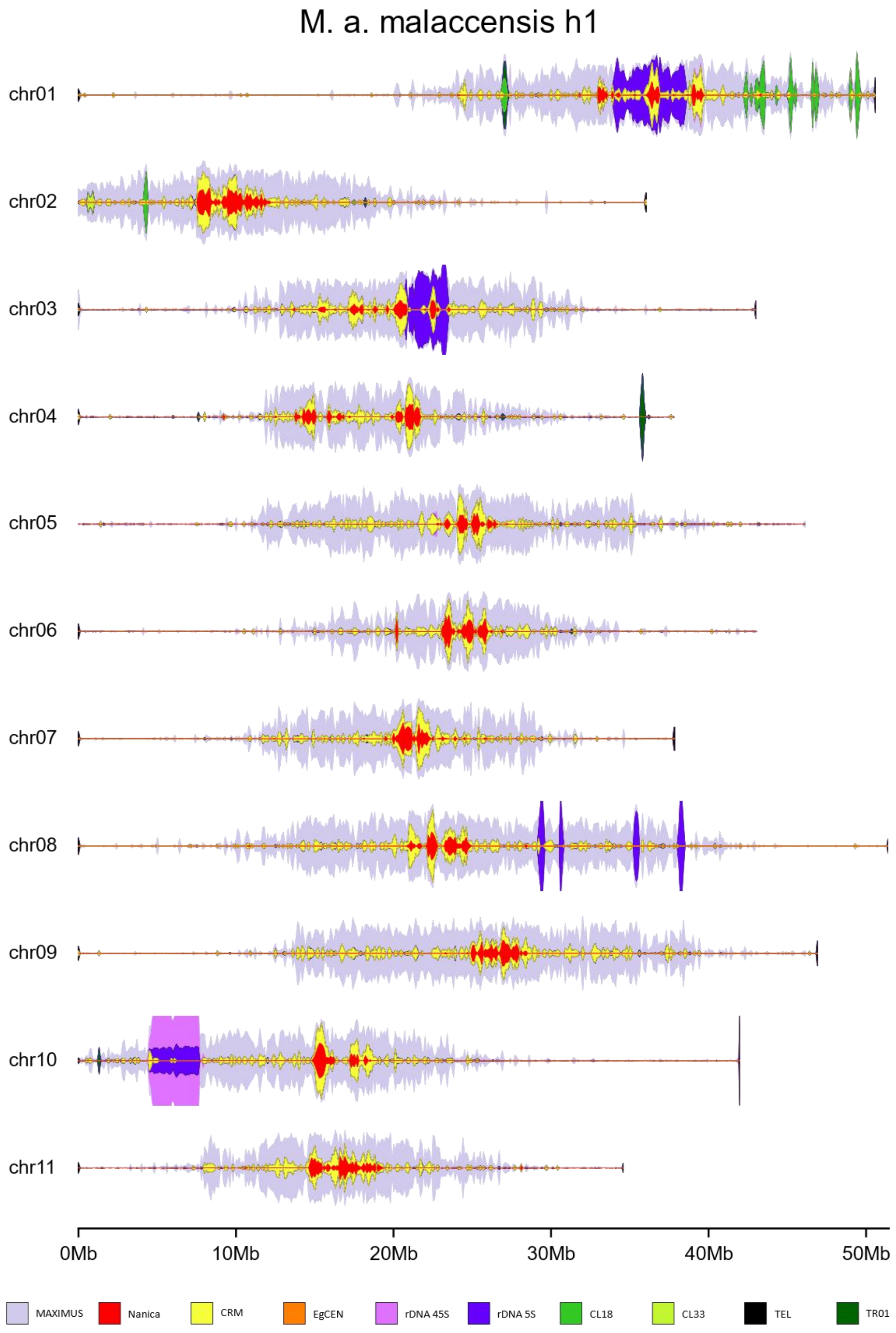

Supplementary Fig. 14. Graphical representation of Centromeric and tandem sequences density on *M. a. malaccensis* H1 genome assembly.

Stacked curves representing the density of SIRE/Maximus transposable elements, centromeric sequences and tandem repeats along the assemblies. Chromosomes are indicated on the left with the genomic scale at the bottom in Mbases. Color codes for repeated sequences classes are indicated. Source data are provided as a Source Data file.

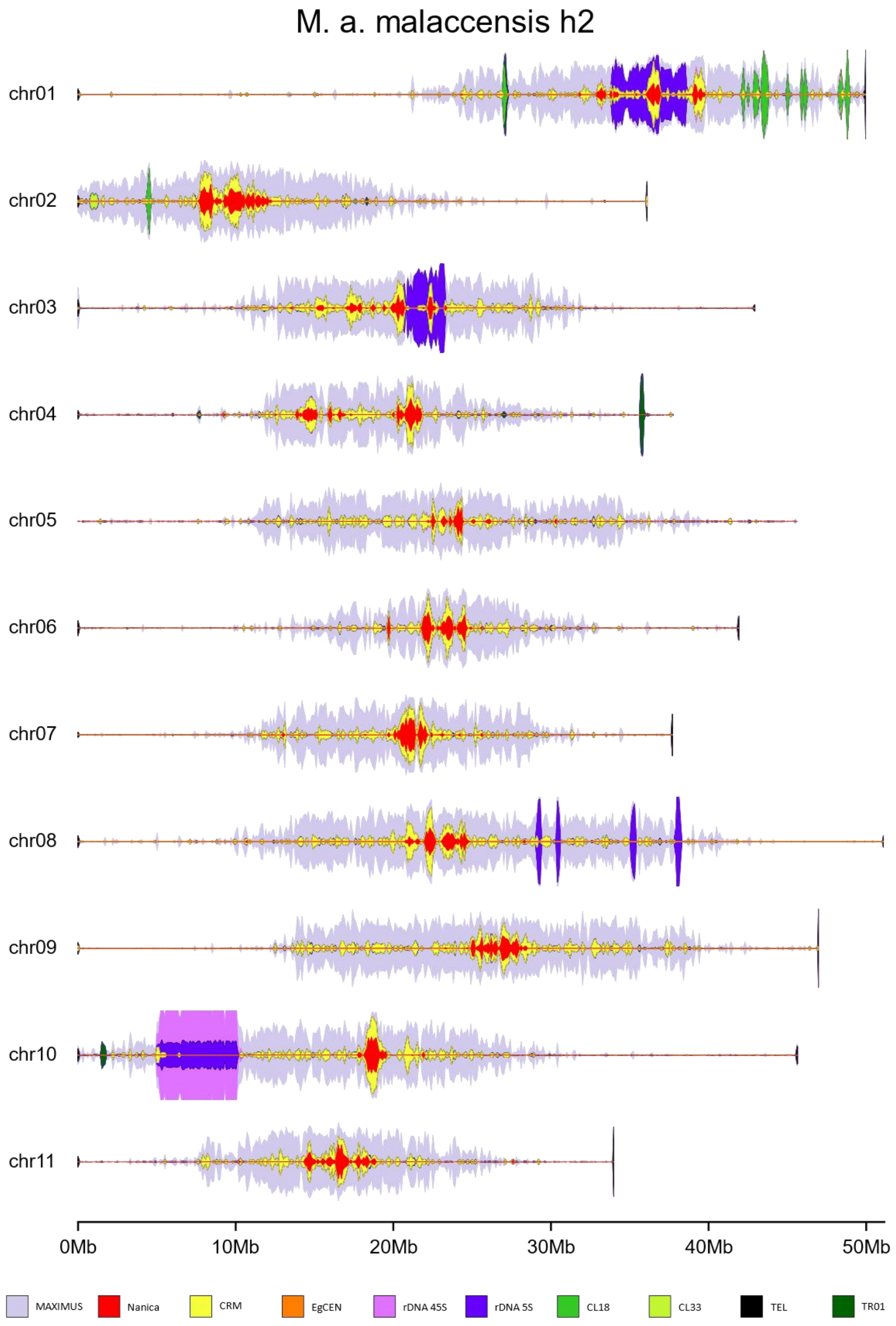

Supplementary Fig. 15. Graphical representation of Centromeric and tandem sequences density on *M. a. malaccensis* H2 genome assembly.

Stacked curves representing the density of SIRE/Maximus transposable elements, centromeric sequences and tandem repeats along the assemblies. Chromosomes are indicated on the left with the genomic scale at the bottom in Mbases. Color codes for repeated sequences classes are indicated. Source data are provided as a Source Data file.

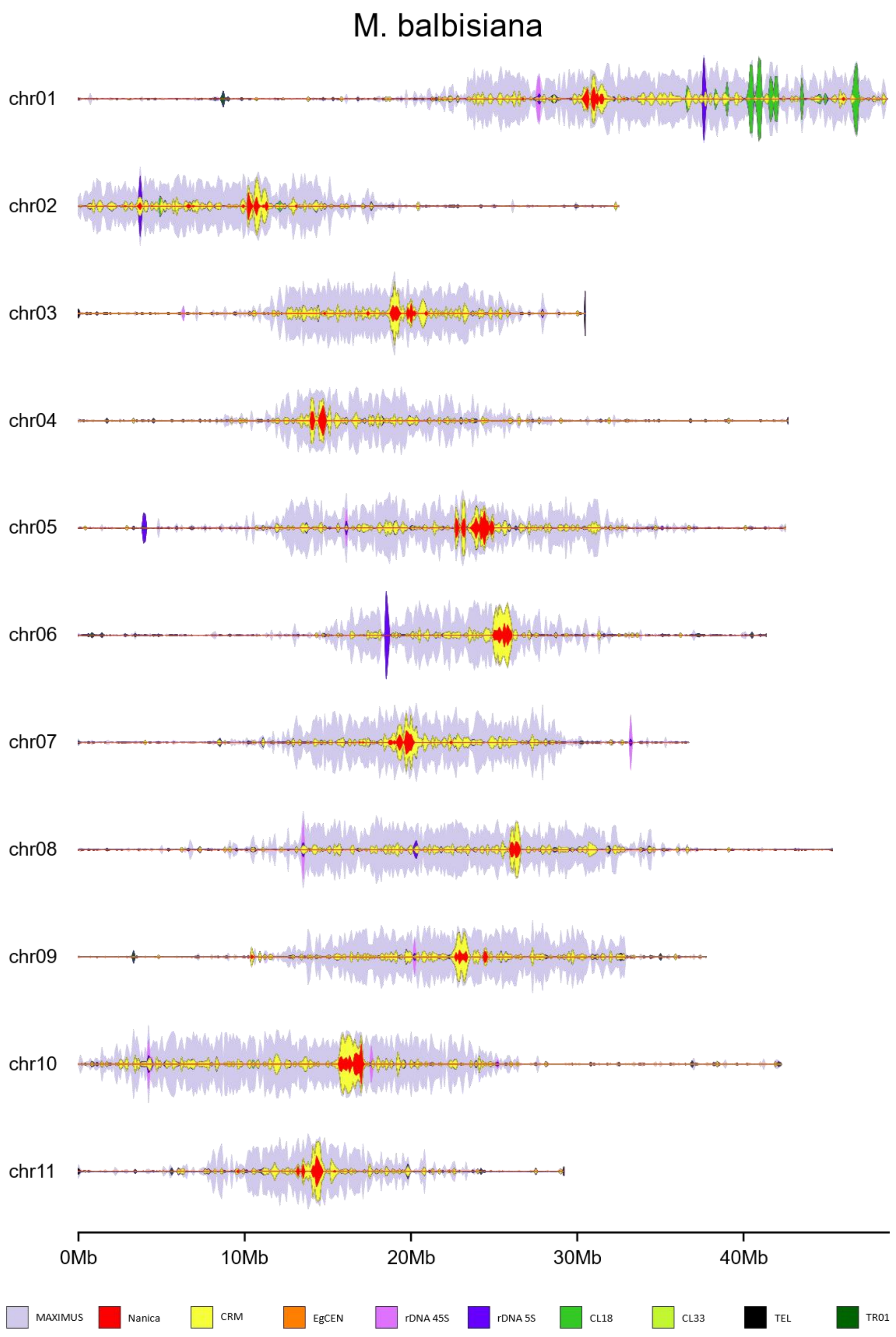

Supplementary Fig. 16. Graphical representation of Centromeric and tandem sequences density on *M. balbisiana* genome assembly.

Stacked curves representing the density of SIRE/Maximus transposable elements, centromeric sequences and tandem repeats along the assemblies. Chromosomes are indicated on the left with the genomic scale at the bottom in Mbases. Color codes for repeated sequences classes are indicated. Source data are provided as a Source Data file.

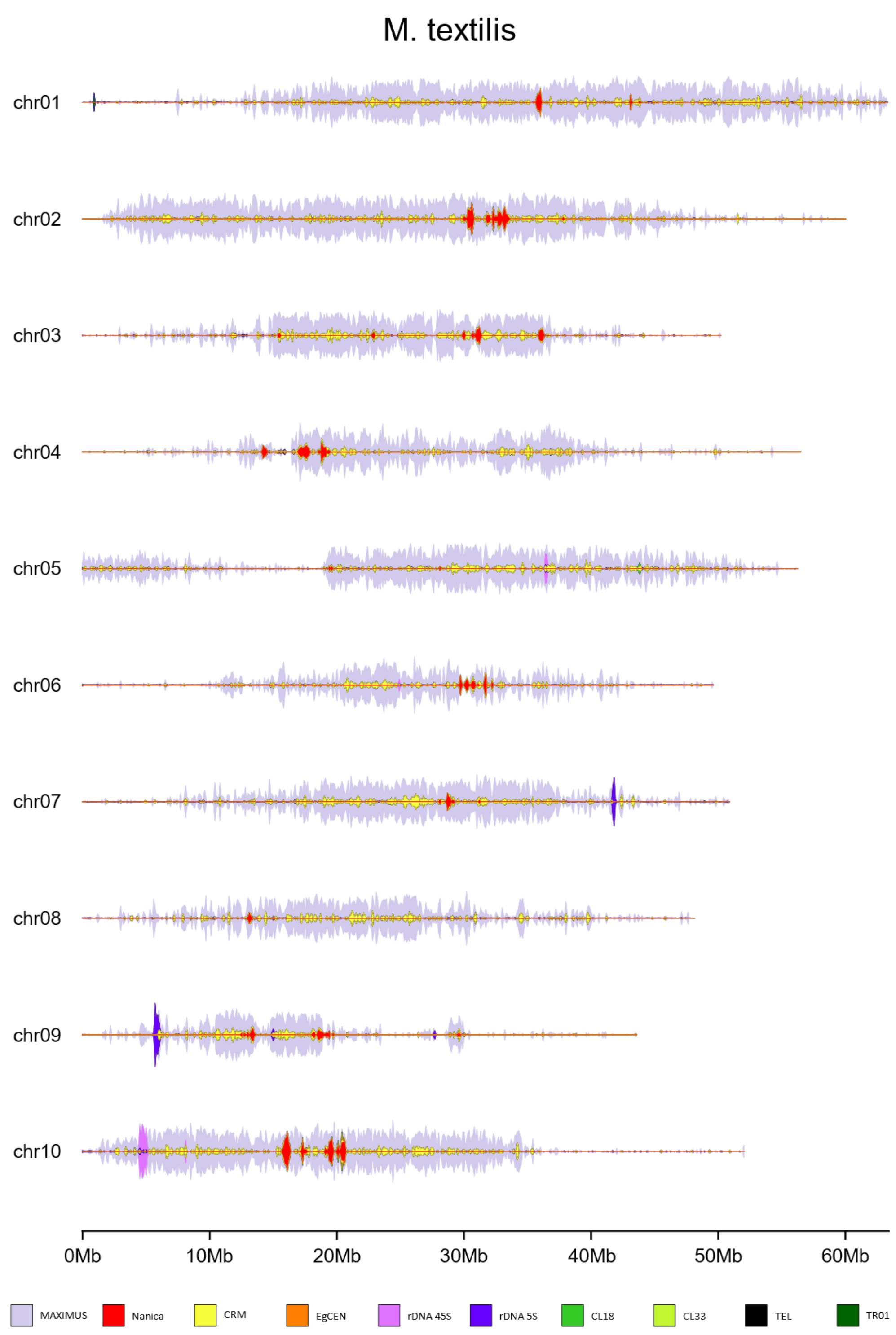

Supplementary Fig. 17. Graphical representation of Centromeric and tandem sequences density on *M. textilis* genome assembly.

Stacked curves representing the density of SIRE/Maximus transposable elements, centromeric sequences and tandem repeats along the assemblies. Chromosomes are indicated on the left with the genomic scale at the bottom in Mbases. Color codes for repeated sequences classes are indicated. Source data are provided as a Source Data file.

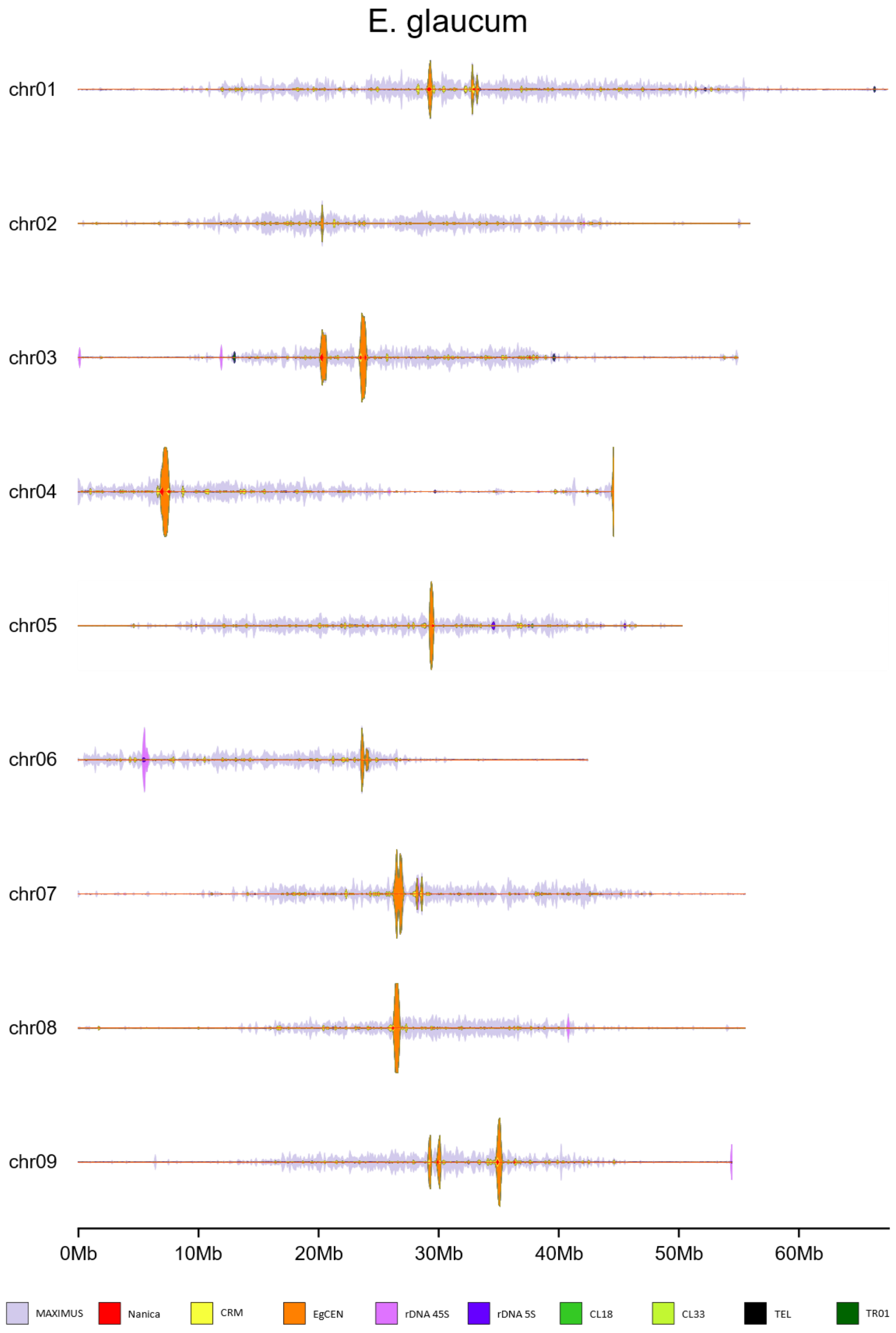

Supplementary Fig. 18. Graphical representation of Centromeric and tandem sequences density on Ensete glaucum genome assembly.

Stacked curves representing the density of SIRE/Maximus transposable elements, centromeric sequences and tandem repeats along the assemblies. Chromosomes are indicated on the left with the genomic scale at the bottom in Mbases. Color codes for repeated sequences classes are indicated. Source data are provided as a Source Data file.

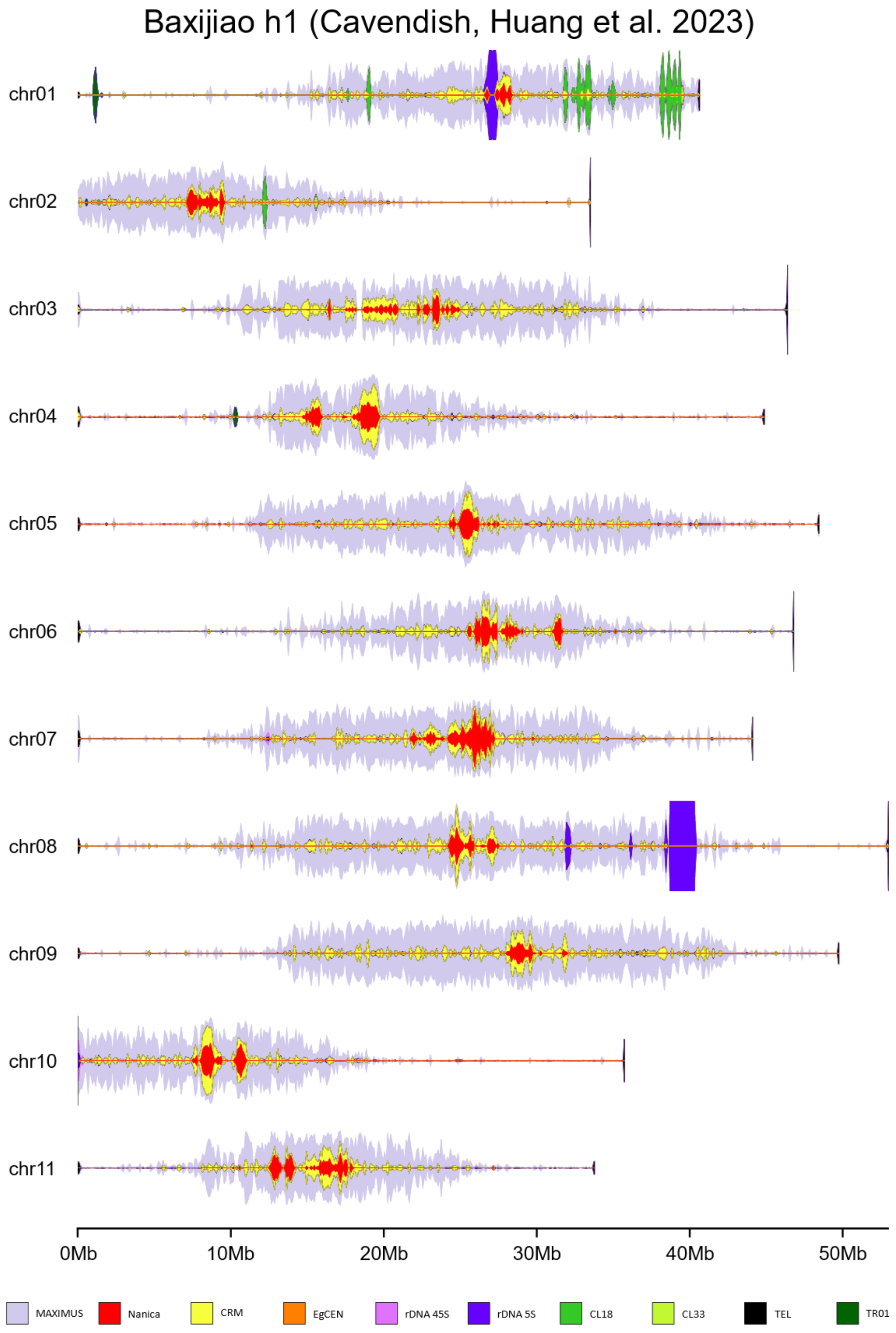

Supplementary Fig. 19. Graphical representation of Centromeric and tandem sequences density on BaxiJiao H1 genome assembly from Huang *et al.* [4].

Stacked curves representing the density of SIRE/Maximus transposable elements, centromeric sequences and tandem repeats along the assemblies. Chromosomes are indicated on the left with the genomic scale at the bottom in Mbases. Color codes for repeated sequences classes are indicated. Source data are provided as a Source Data file.

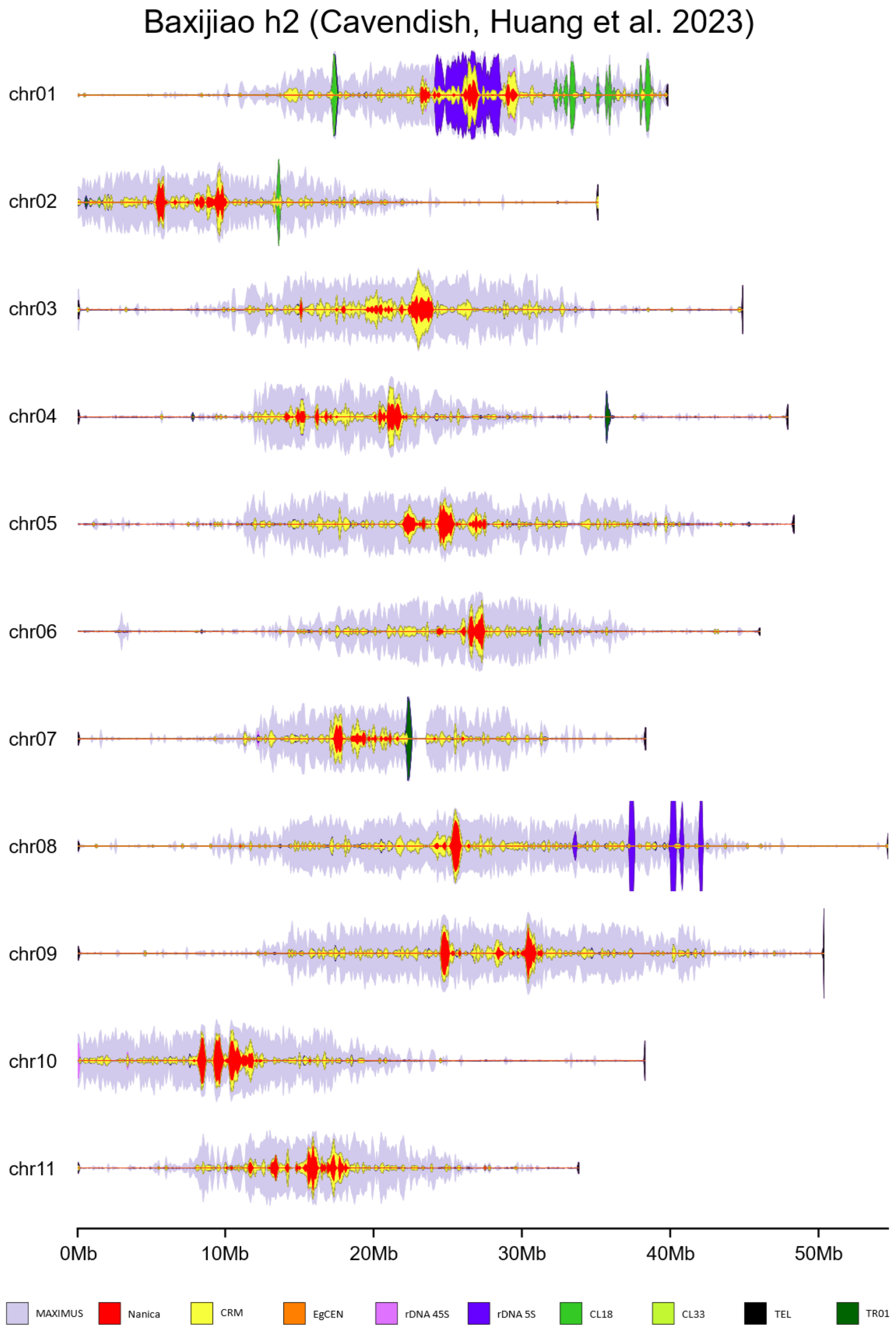

Supplementary Fig. 20. Graphical representation of Centromeric and tandem sequences density on BaxiJiao H2 genome assembly from Huang *et al.* [4].
Stacked curves representing the density of SIRE/Maximus transposable elements, centromeric sequences and tandem repeats along the assemblies. Chromosomes are indicated on the left with the genomic scale at the bottom in Mbases. Color codes for repeated sequences classes are indicated. Source data are provided as a Source Data file.

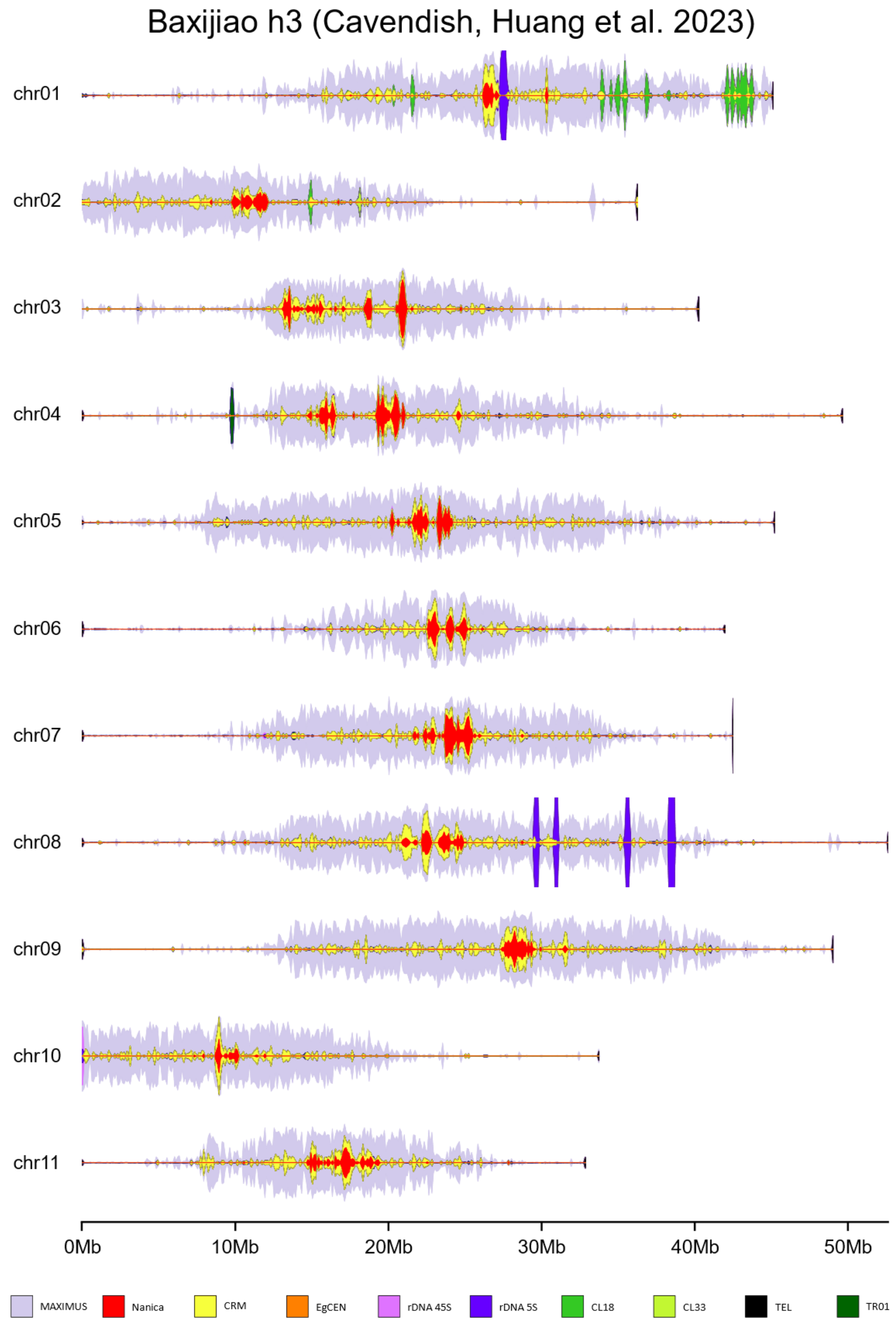

Supplementary Fig. 21. Graphical representation of Centromeric and tandem sequences density on BaxiJiao H3 genome assembly from Huang *et al.* [4].
Stacked curves representing the density of SIRE/Maximus transposable elements, centromeric sequences and tandem repeats along the assemblies. Chromosomes are indicated on the left with the genomic scale at the bottom in Mbases. Color codes for repeated sequences classes are indicated. Source data are provided as a Source Data file.

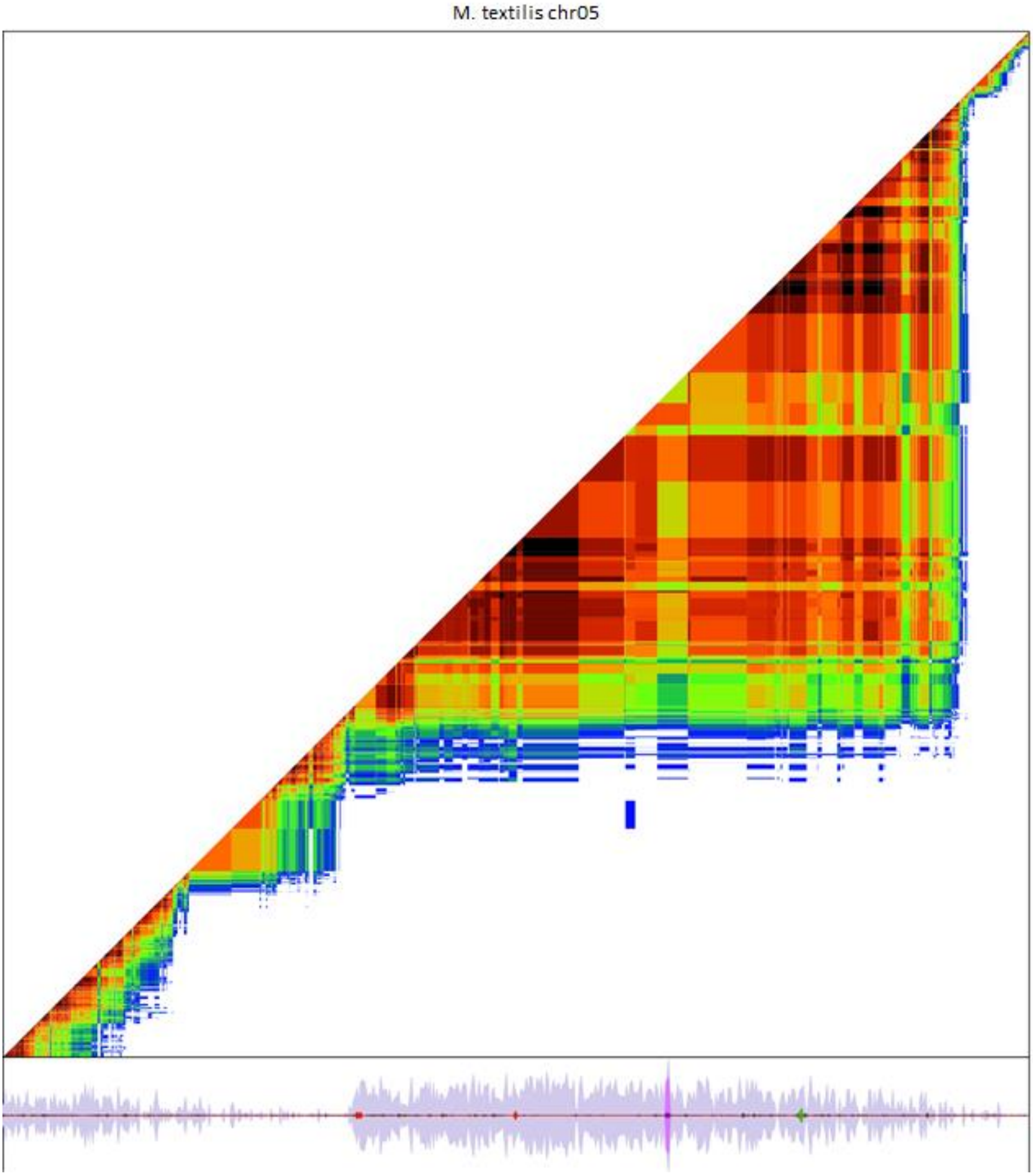

Supplementary Fig. 22. Sequence assembly and pairwise genetic distance of *M. textilis* chromosome 05.
Stacked curves representing the density of SIRE/Maximus transposable elements (grey) tandem repeats (green) and Nanica centromeric repeats (red) along the assemblies of *M. textilis* chromosome 05 (bottom) is compared to pairwise marker genetic distance with a warm-cool color gradient from dark red to blue for strong linkage and weak linkage, respectively. Pairwise genetic distances for chromosome 05 markers are calculated from GBS data of a self-progeny of *M. textilis* and plotted onto assembly (*M. textilis* chr05). Source data are provided as a Source Data file.

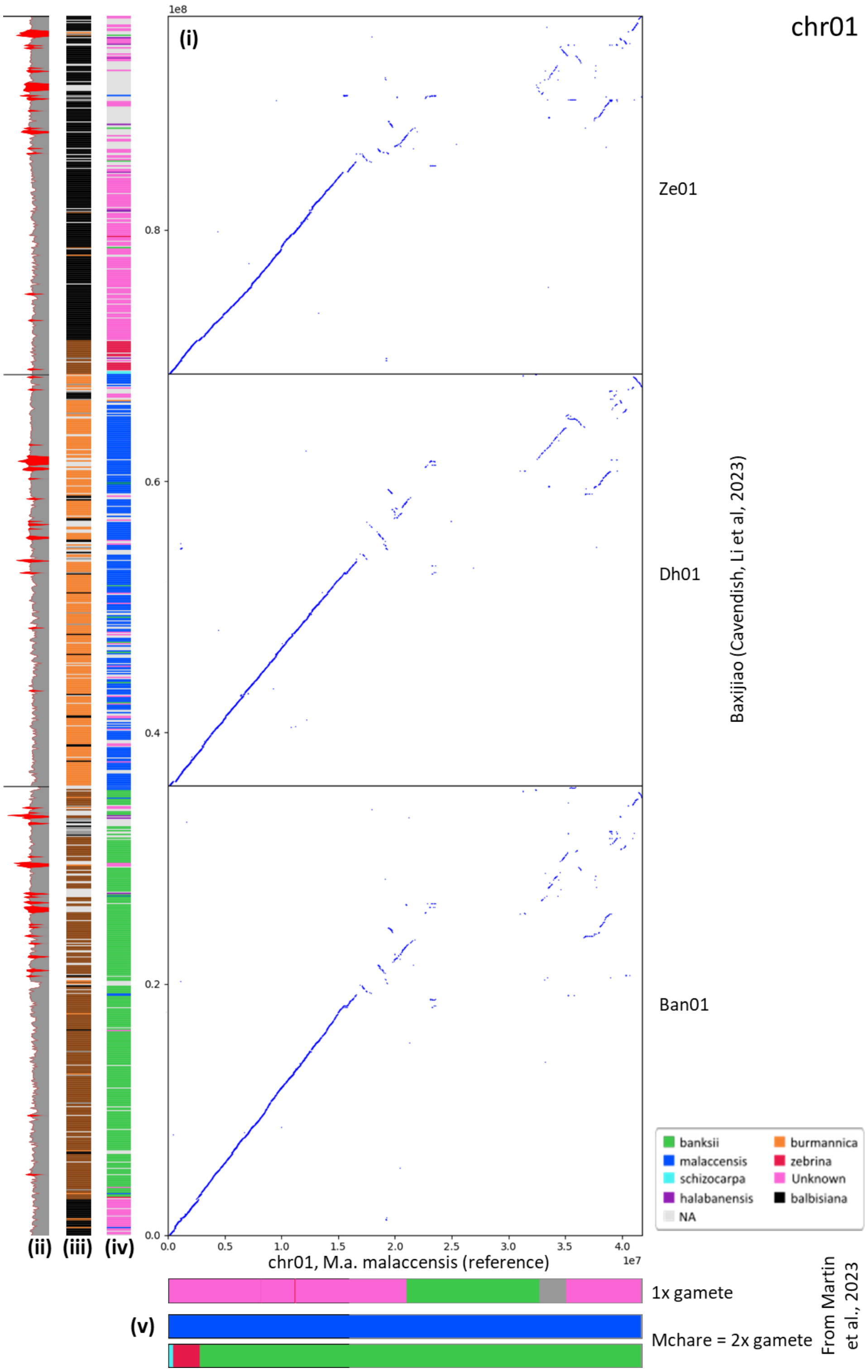

Supplementary Fig. 23. Assessment of Chromosome 01 of Cavendish assembly from Li *et al.*[6].

Cavendish assembly was evaluated using (i) a dot-plot comparison with the *M. a. malaccensis* reference assembly, (ii) Cavendish Illumina read coverage (SRR23425445) along the haplotype assembly, (iii) *in silico* chromosome painting based on hypothesised gametes haplotypes from Martin *et al.*[5] and (iv) *in silico* Cavendish assembly ancestry painting comparison to Cavendish mosaic painting of Martin *et al.*[6] (v). The average read coverage along assembly (ii) was calculated along chromosomes on sliding windows of 100kb. Overcovered regions, likely resulting from the collapse of two or more haplotypes or repeat regions, are distinguished from the normally covered regions (in grey) by a different red colour. In (iii), the black colour is for the 1x gamete and orange and brown for the two haplotypes of the 2x gamete. Regions in grey represent regions transmitted by the 2x gamete but for which it is not possible to determine whether they belong to haplotype 1 or 2 because of homozygosity between the two haplotypes. The legend for ancestry painting (iv and v) is located on the right bottom corner of each figure. Source data are provided as a Source Data file.

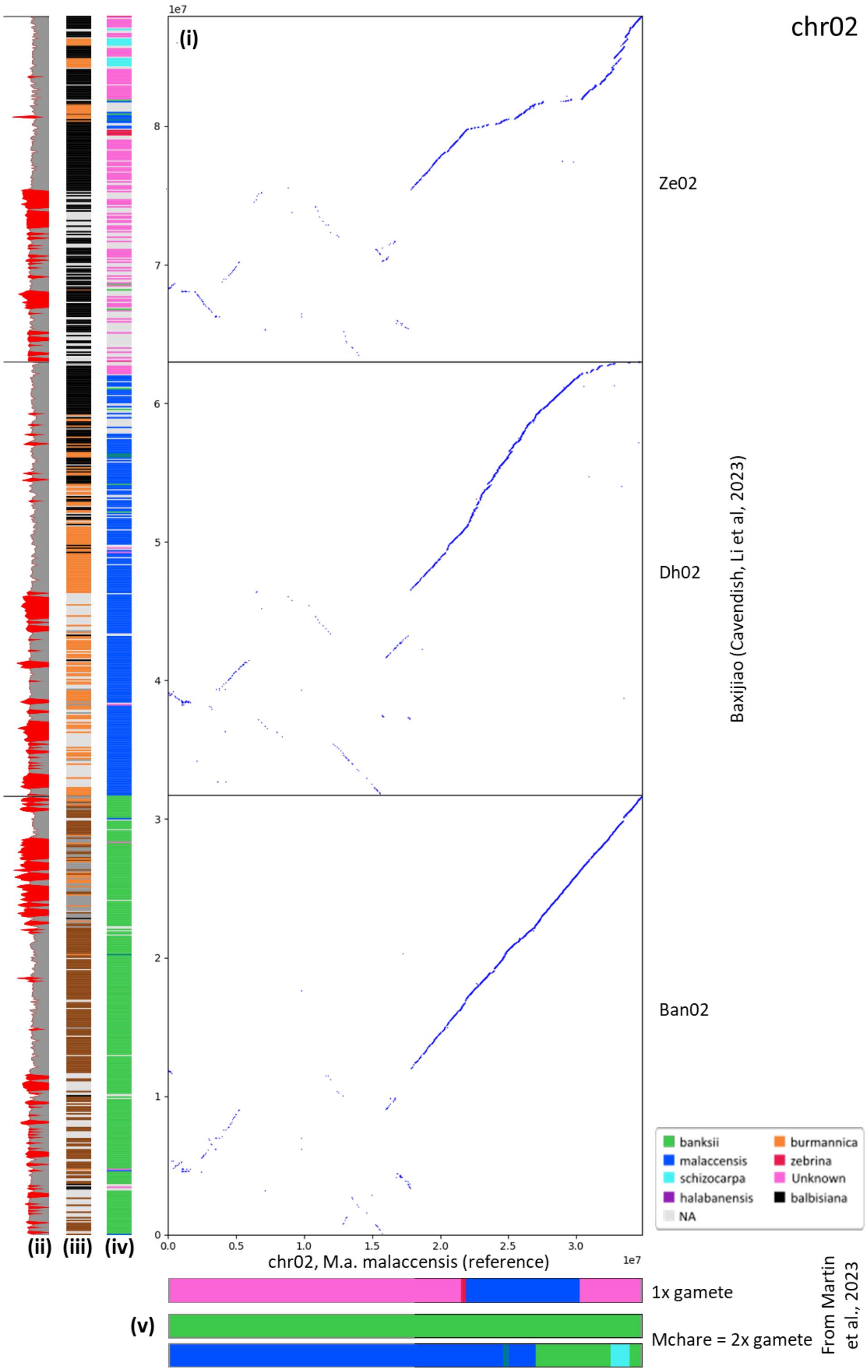

Supplementary Fig. 24. Assessment of Chromosome 02 of Cavendish assembly from Li *et al.* [6].

Cavendish assembly was evaluated using (i) a dot-plot comparison with the *M. a. malaccensis* reference assembly, (ii) Cavendish Illumina read coverage (SRR23425445) along the haplotype assembly, (iii) *in silico* chromosome painting based on hypothesised gametes haplotypes from Martin *et al*.[5] and (iv) *in silico* Cavendish assembly ancestry painting comparison to Cavendish mosaic painting of Martin *et al*.[6] (v). The average read coverage along assembly (ii) was calculated along chromosomes on sliding windows of 100kb. Overcovered regions, likely resulting from the collapse of two or more haplotypes or repeat regions, are distinguished from the normally covered regions (in grey) by a different red colour. In (iii), the black colour is for the 1x gamete and orange and brown for the two haplotypes of the 2x gamete. Regions in grey represent regions transmitted by the 2x gamete but for which it is not possible to determine whether they belong to haplotype 1 or 2 because of homozygosity between the two haplotypes. The legend for ancestry painting (iv and v) is located on the right bottom corner of each figure. Source data are provided as a Source Data file.

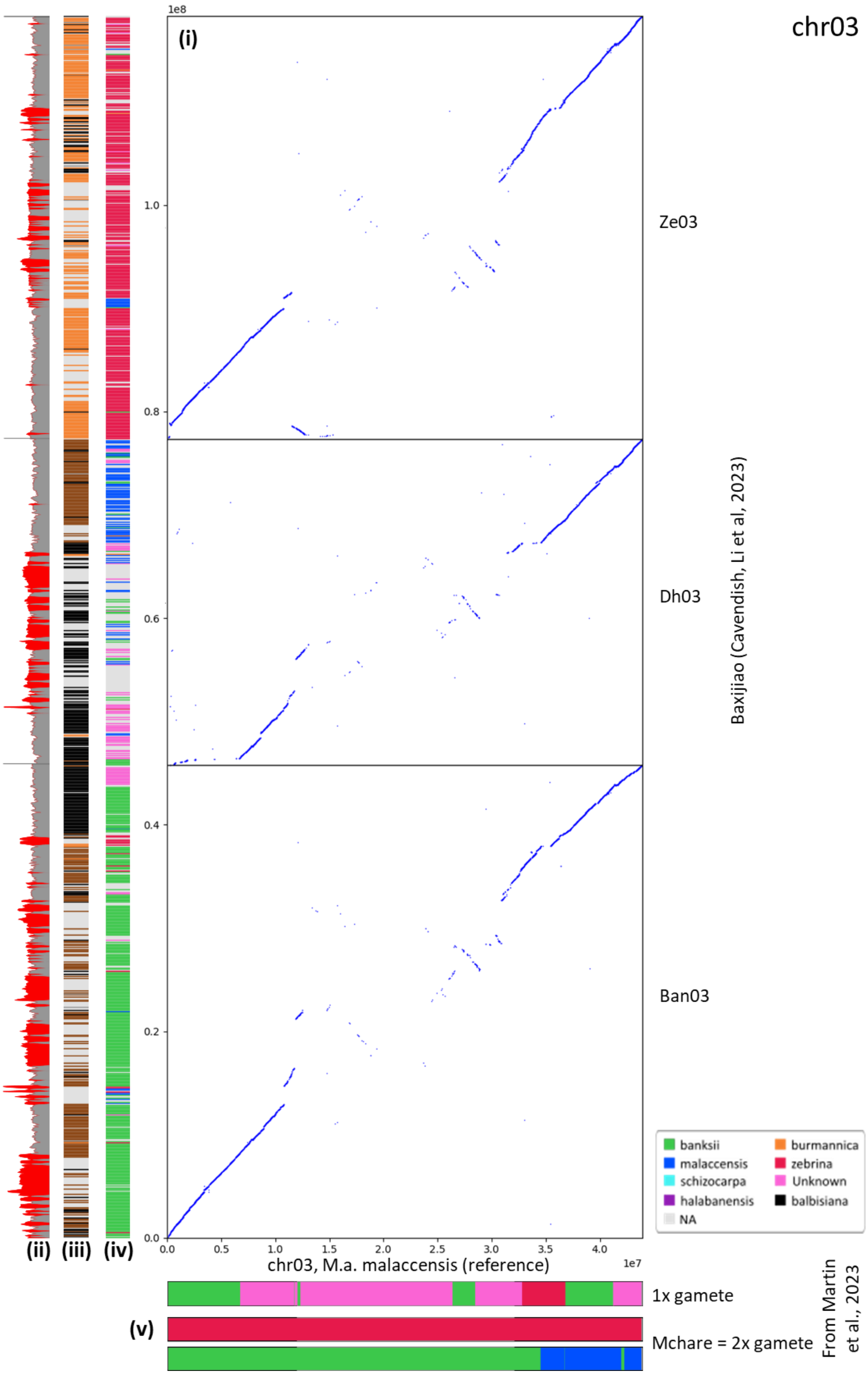

Supplementary Fig. 25. Assessment of Chromosome 03 of Cavendish assembly from Li *et al.* [6].
Cavendish assembly was evaluated using (i) a dot-plot comparison with the *M. a. malaccensis* reference assembly, (ii) Cavendish Illumina read coverage (SRR23425445) along the haplotype assembly, (iii) *in silico* chromosome painting based on hypothesised gametes haplotypes from Martin *et al*.[5] and (iv) *in silico* Cavendish assembly ancestry painting comparison to Cavendish mosaic painting of Martin *et al*.[6] (v). The average read coverage along assembly (ii) was calculated along chromosomes on sliding windows of 100kb. Overcovered regions, likely resulting from the collapse of two or more haplotypes or repeat regions, are distinguished from the normally covered regions (in grey) by a different red colour. In (iii), the black colour is for the 1x gamete and orange and brown for the two haplotypes of the 2x gamete. Regions in grey represent regions transmitted by the 2x gamete but for which it is not possible to determine whether they belong to haplotype 1 or 2 because of homozygosity between the two haplotypes. The legend for ancestry painting (iv and v) is located on the right bottom corner of each figure. Source data are provided as a Source Data file.

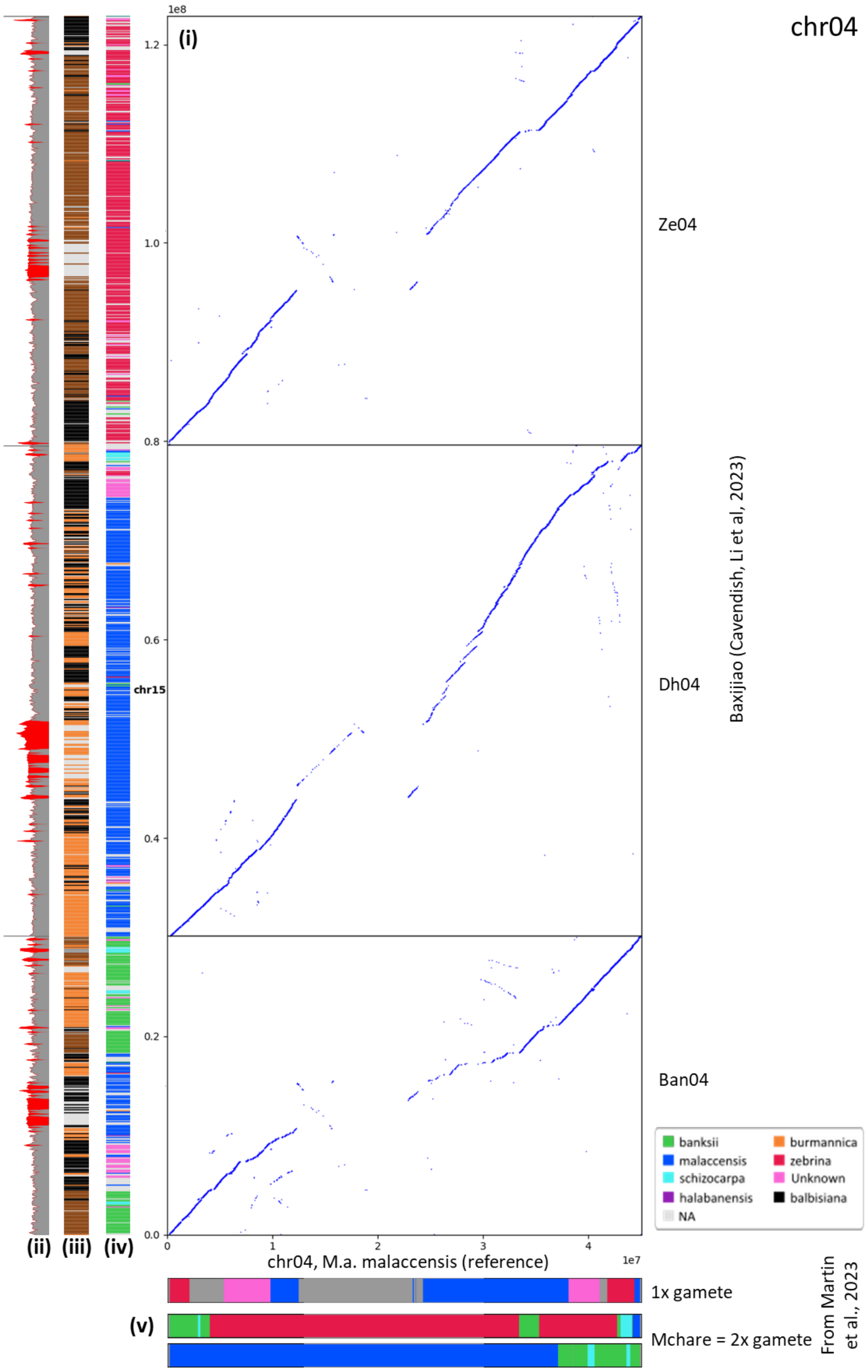

Supplementary Fig. 26. Assessment of Chromosome 04 of Cavendish assembly from Li *et al.* [6].

Cavendish assembly was evaluated using (i) a dot-plot comparison with the *M. a. malaccensis* reference assembly, (ii) Cavendish Illumina read coverage (SRR23425445) along the haplotype assembly, (iii) *in silico* chromosome painting based on hypothesised gametes haplotypes from Martin *et al*.[5] and (iv) *in silico* Cavendish assembly ancestry painting comparison to Cavendish mosaic painting of Martin *et al*.[6] (v). The average read coverage along assembly (ii) was calculated along chromosomes on sliding windows of 100kb. Overcovered regions, likely resulting from the collapse of two or more haplotypes or repeat regions, are distinguished from the normally covered regions (in grey) by a different red colour. In (iii), the black colour is for the 1x gamete and orange and brown for the two haplotypes of the 2x gamete. Regions in grey represent regions transmitted by the 2x gamete but for which it is not possible to determine whether they belong to haplotype 1 or 2 because of homozygosity between the two haplotypes. The legend for ancestry painting (iv and v) is located on the right bottom corner of each figure. Source data are provided as a Source Data file.

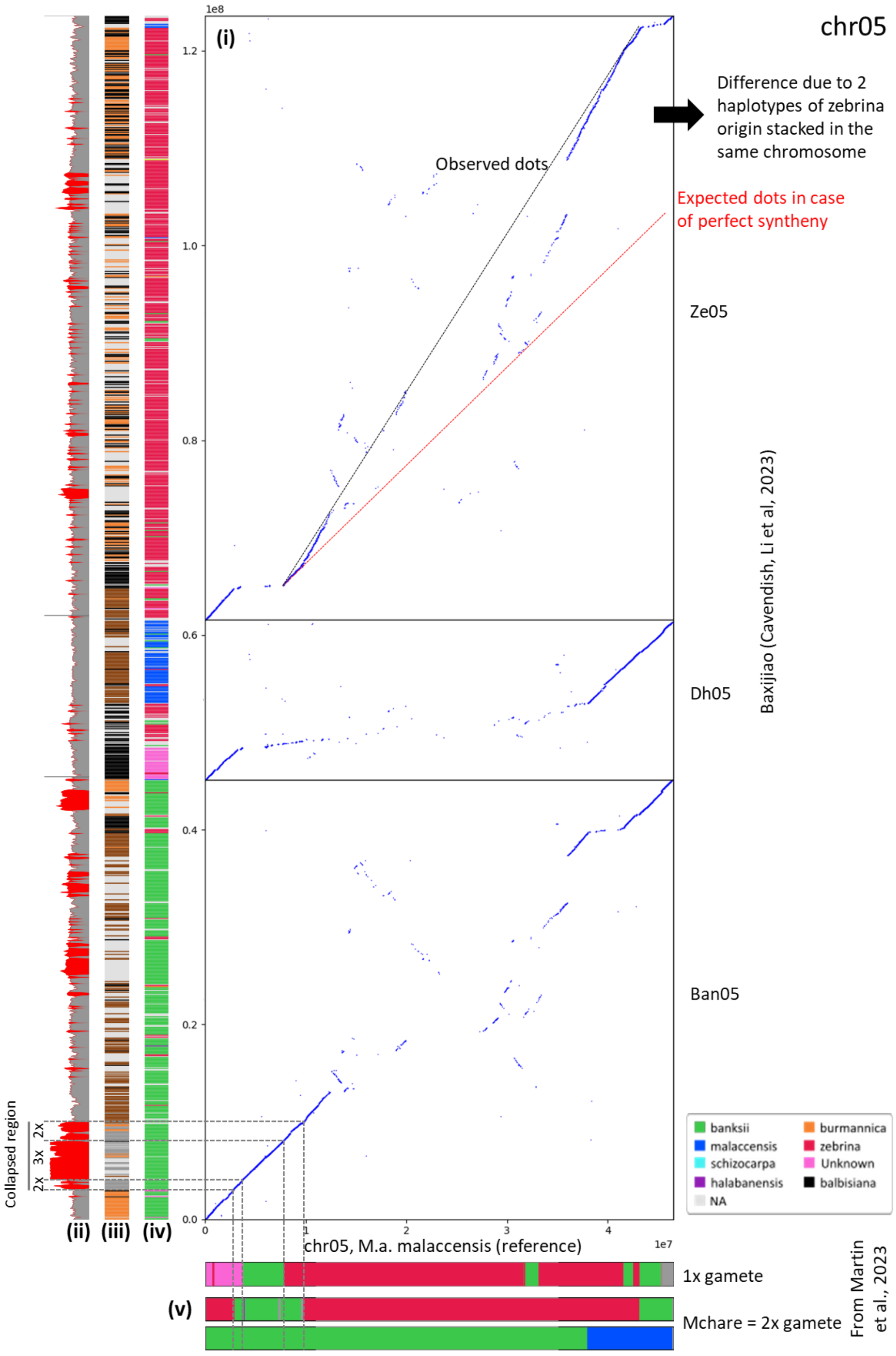

Supplementary Fig. 27. Assessment of Chromosome 05 of Cavendish assembly from Li *et al.* [6].
Same legend as Supplementary Fig. 23. Examples of collapsed regions probably due to homozygosity in 2 or three haplotypes can be observed here. The region is only present in one or two of the three haplotype assemblies (dot-plot) but read coverage information suggests that these regions are present in three copies as expected from a triploid. In e, the red dashed line corresponds to the expectation in case of perfect synteny between the *M. a. malaccensis* reference assembly and Ze05 chromosome assembly. The black dashed line corresponds to the average synteny observed between the two assemblies in the region. The observed difference between the red and the black lines suggest that the Ze05 chromosome has more sequence than chr05 in the *M. a. malaccensis* reference assembly. This is the result of stacked regions from two chromosome 5 haplotypes of Cavendish into one chromosome due to heterozygosity in two haplotypes of the same ancestral origin. Source data are provided as a Source Data file.

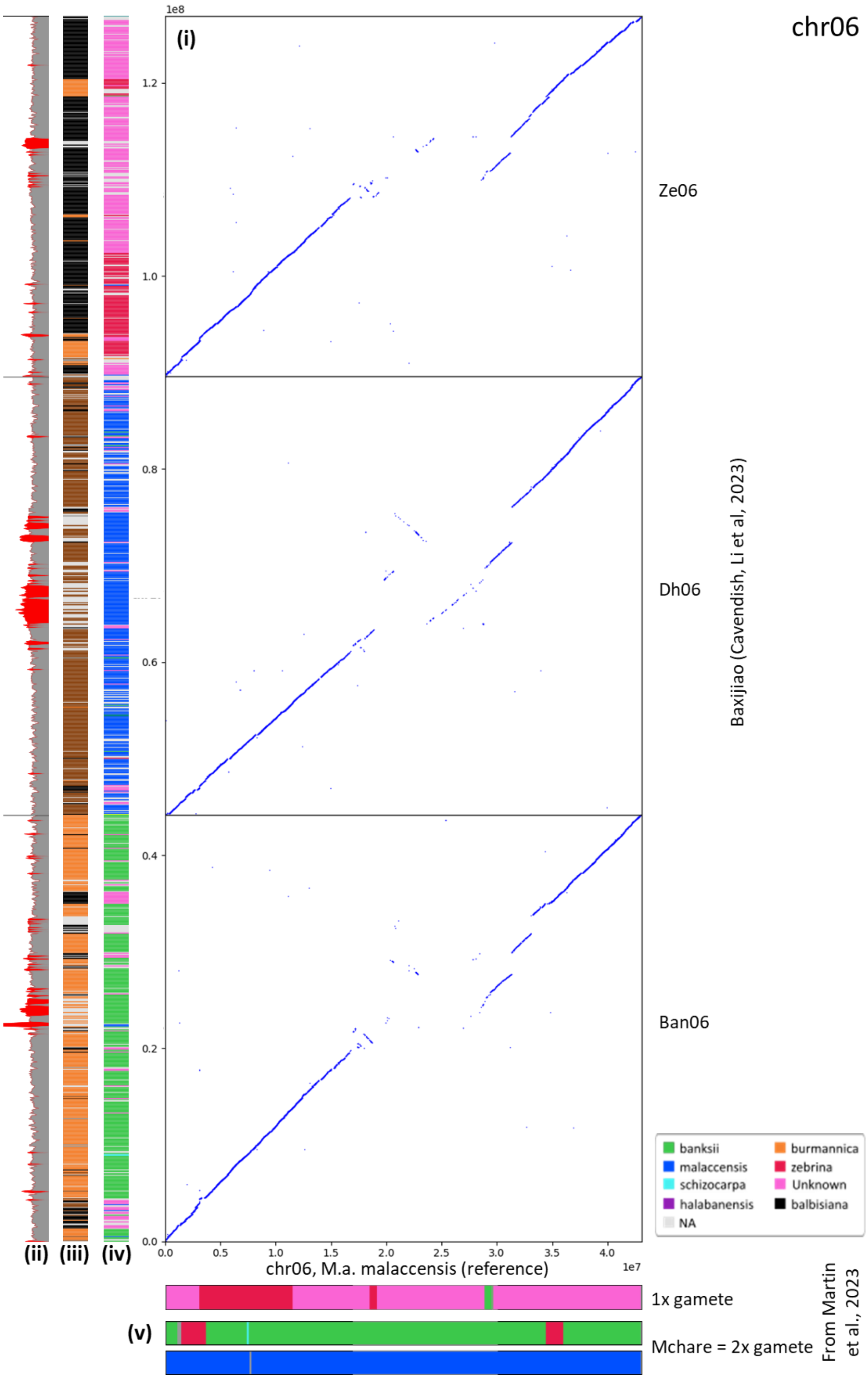

Supplementary Fig. 28. Assessment of Chromosome 06 of Cavendish assembly from Li *et al.* [6].
Cavendish assembly was evaluated using (i) a dot-plot comparison with the *M. a. malaccensis* reference assembly, (ii) Cavendish Illumina read coverage (SRR23425445) along the haplotype assembly, (iii) *in silico* chromosome painting based on hypothesised gametes haplotypes from Martin *et al.*[5] and (iv) *in silico* Cavendish assembly ancestry painting comparison to Cavendish mosaic painting of Martin *et al.*[6] (v). The average read coverage along assembly (ii) was calculated along chromosomes on sliding windows of 100kb. Overcovered regions, likely resulting from the collapse of two or more haplotypes or repeat regions, are distinguished from the normally covered regions (in grey) by a different red colour. In (iii), the black colour is for the 1x gamete and orange and brown for the two haplotypes of the 2x gamete. Regions in grey represent regions transmitted by the 2x gamete but for which it is not possible to determine whether they belong to haplotype 1 or 2 because of homozygosity between the two haplotypes. The legend for ancestry painting (iv and v) is located on the right bottom corner of each figure. Source data are provided as a Source Data file.

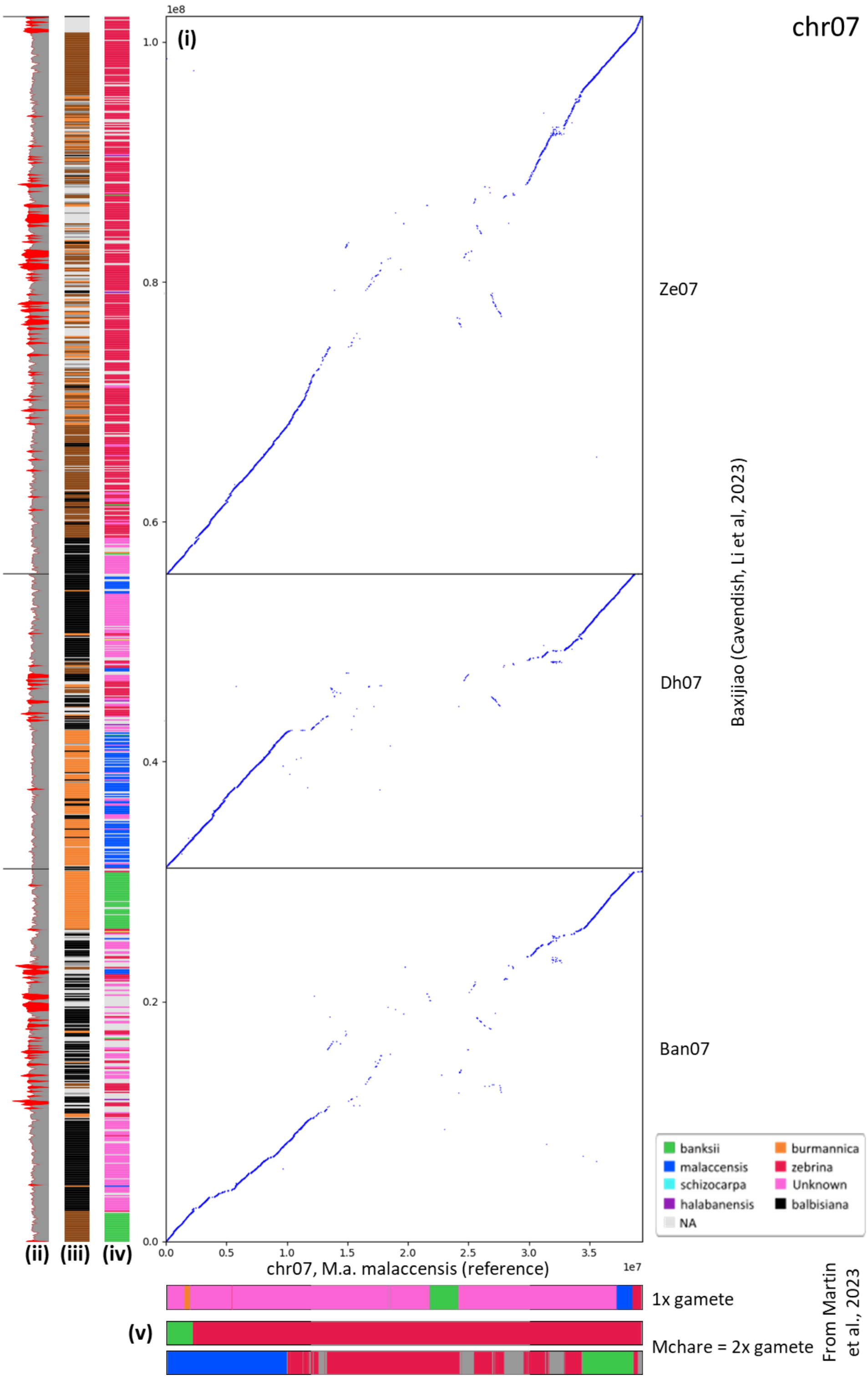

Supplementary Fig. 29. Assessment of Chromosome 07 of Cavendish assembly from Li *et al.* [6].
Cavendish assembly was evaluated using (i) a dot-plot comparison with the *M. a. malaccensis* reference assembly, (ii) Cavendish Illumina read coverage (SRR23425445) along the haplotype assembly, (iii) *in silico* chromosome painting based on hypothesised gametes haplotypes from Martin *et al*.[5] and (iv) *in silico* Cavendish assembly ancestry painting comparison to Cavendish mosaic painting of Martin *et al*.[6] (v). The average read coverage along assembly (ii) was calculated along chromosomes on sliding windows of 100kb. Overcovered regions, likely resulting from the collapse of two or more haplotypes or repeat regions, are distinguished from the normally covered regions (in grey) by a different red colour. In (iii), the black colour is for the 1x gamete and orange and brown for the two haplotypes of the 2x gamete. Regions in grey represent regions transmitted by the 2x gamete but for which it is not possible to determine whether they belong to haplotype 1 or 2 because of homozygosity between the two haplotypes. The legend for ancestry painting (iv and v) is located on the right bottom corner of each figure. Source data are provided as a Source Data file.

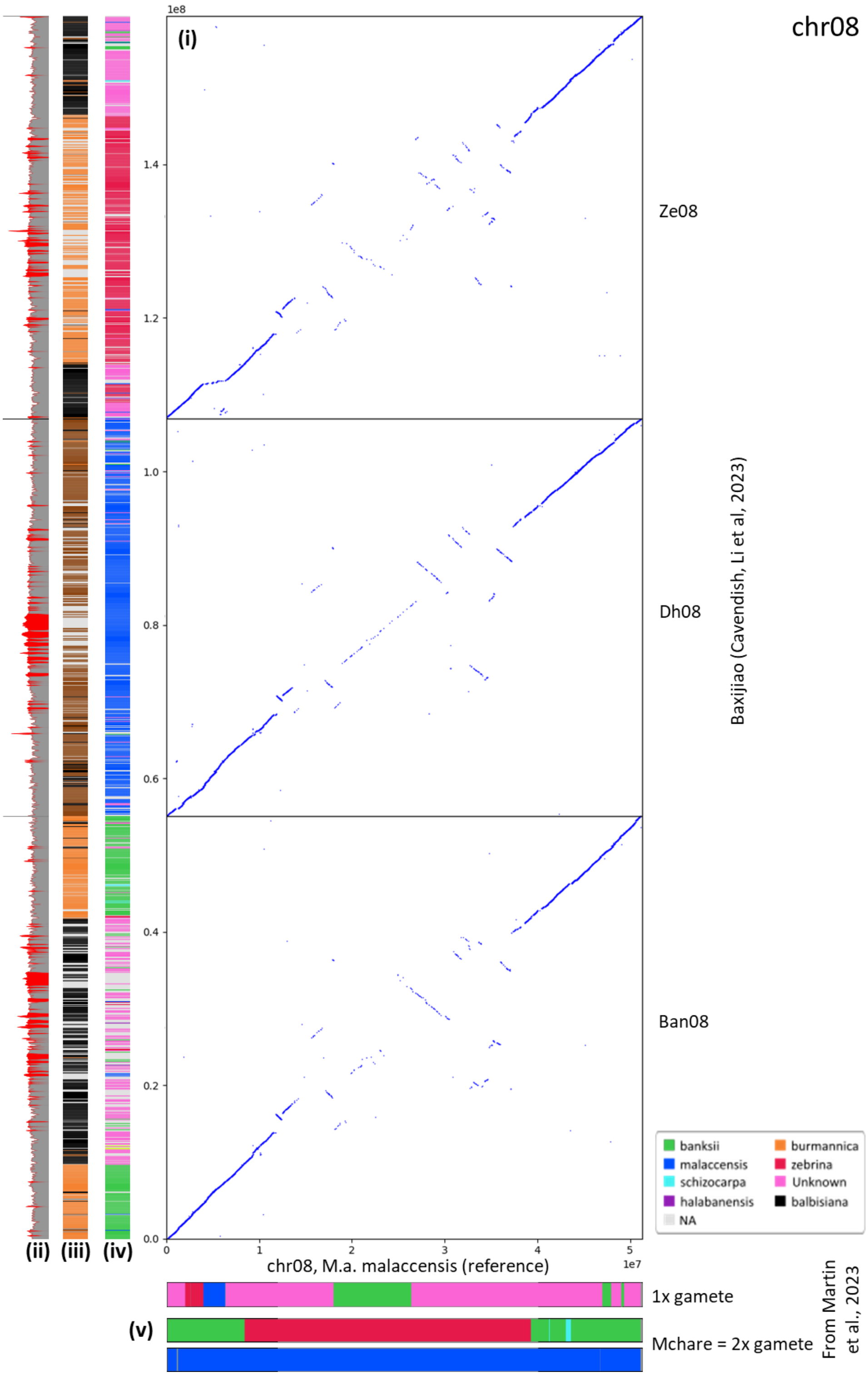

Supplementary Fig. 30. Assessment of Chromosome 08 of Cavendish assembly from Li *et al.* [6].
Cavendish assembly was evaluated using (i) a dot-plot comparison with the *M. a. malaccensis* reference assembly, (ii) Cavendish Illumina read coverage (SRR23425445) along the haplotype assembly, (iii) *in silico* chromosome painting based on hypothesised gametes haplotypes from Martin *et al*.[5] and (iv) *in silico* Cavendish assembly ancestry painting comparison to Cavendish mosaic painting of Martin *et al*.[6] (v). The average read coverage along assembly (ii) was calculated along chromosomes on sliding windows of 100kb. Overcovered regions, likely resulting from the collapse of two or more haplotypes or repeat regions, are distinguished from the normally covered regions (in grey) by a different red colour. In (iii), the black colour is for the 1x gamete and orange and brown for the two haplotypes of the 2x gamete. Regions in grey represent regions transmitted by the 2x gamete but for which it is not possible to determine whether they belong to haplotype 1 or 2 because of homozygosity between the two haplotypes. The legend for ancestry painting (iv and v) is located on the right bottom corner of each figure. Source data are provided as a Source Data file.

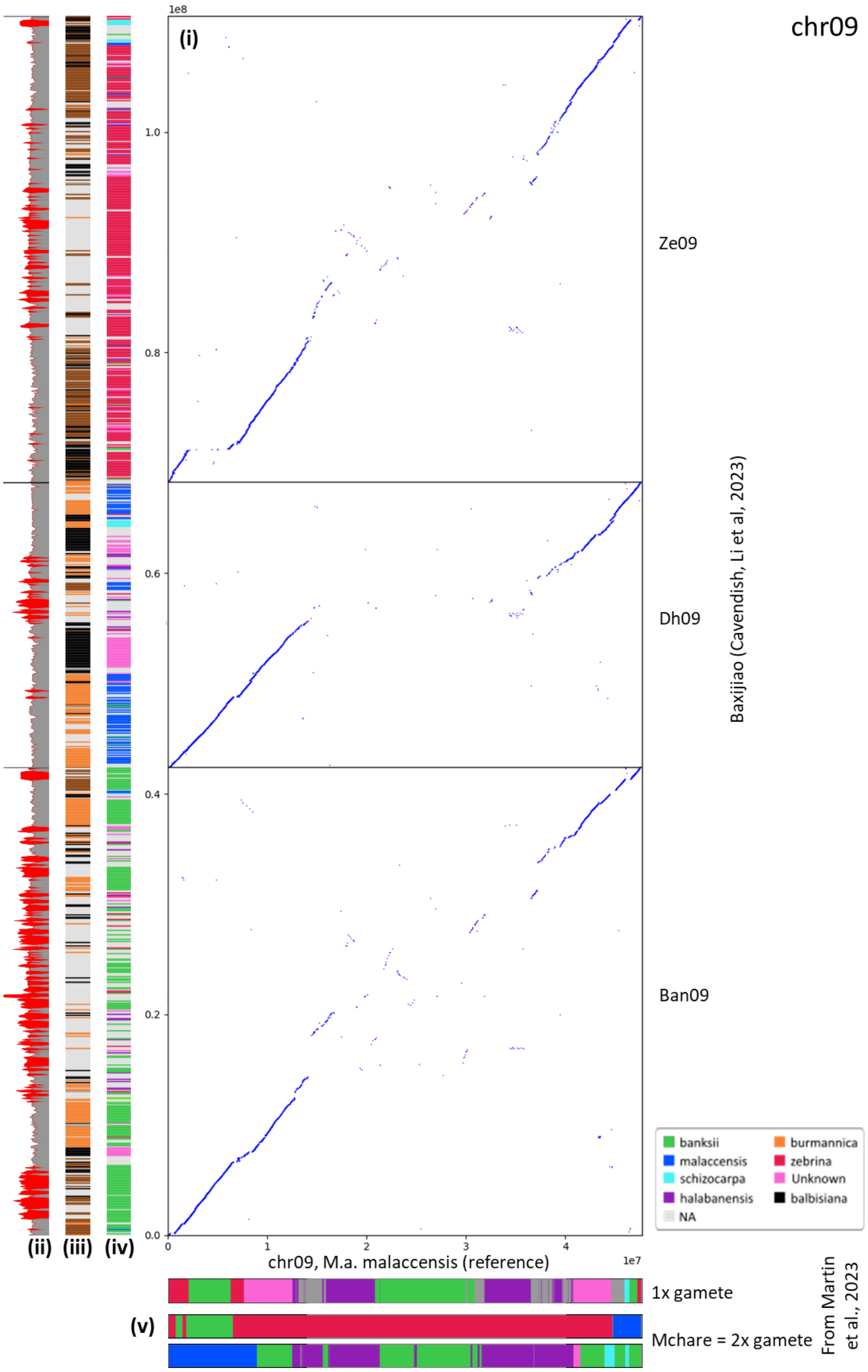

Supplementary Fig. 31. Assessment of Chromosome 09 of Cavendish assembly from Li *et al.* [6].
Cavendish assembly was evaluated using (i) a dot-plot comparison with the *M. a. malaccensis* reference assembly, (ii) Cavendish Illumina read coverage (SRR23425445) along the haplotype assembly, (iii) *in silico* chromosome painting based on hypothesised gametes haplotypes from Martin *et al.*[5] and (iv) *in silico* Cavendish assembly ancestry painting comparison to Cavendish mosaic painting of Martin *et al.*[6] (v). The average read coverage along assembly (ii) was calculated along chromosomes on sliding windows of 100kb. Overcovered regions, likely resulting from the collapse of two or more haplotypes or repeat regions, are distinguished from the normally covered regions (in grey) by a different red colour. In (iii), the black colour is for the 1x gamete and orange and brown for the two haplotypes of the 2x gamete. Regions in grey represent regions transmitted by the 2x gamete but for which it is not possible to determine whether they belong to haplotype 1 or 2 because of homozygosity between the two haplotypes. The legend for ancestry painting (iv and v) is located on the right bottom corner of each figure. Source data are provided as a Source Data file.

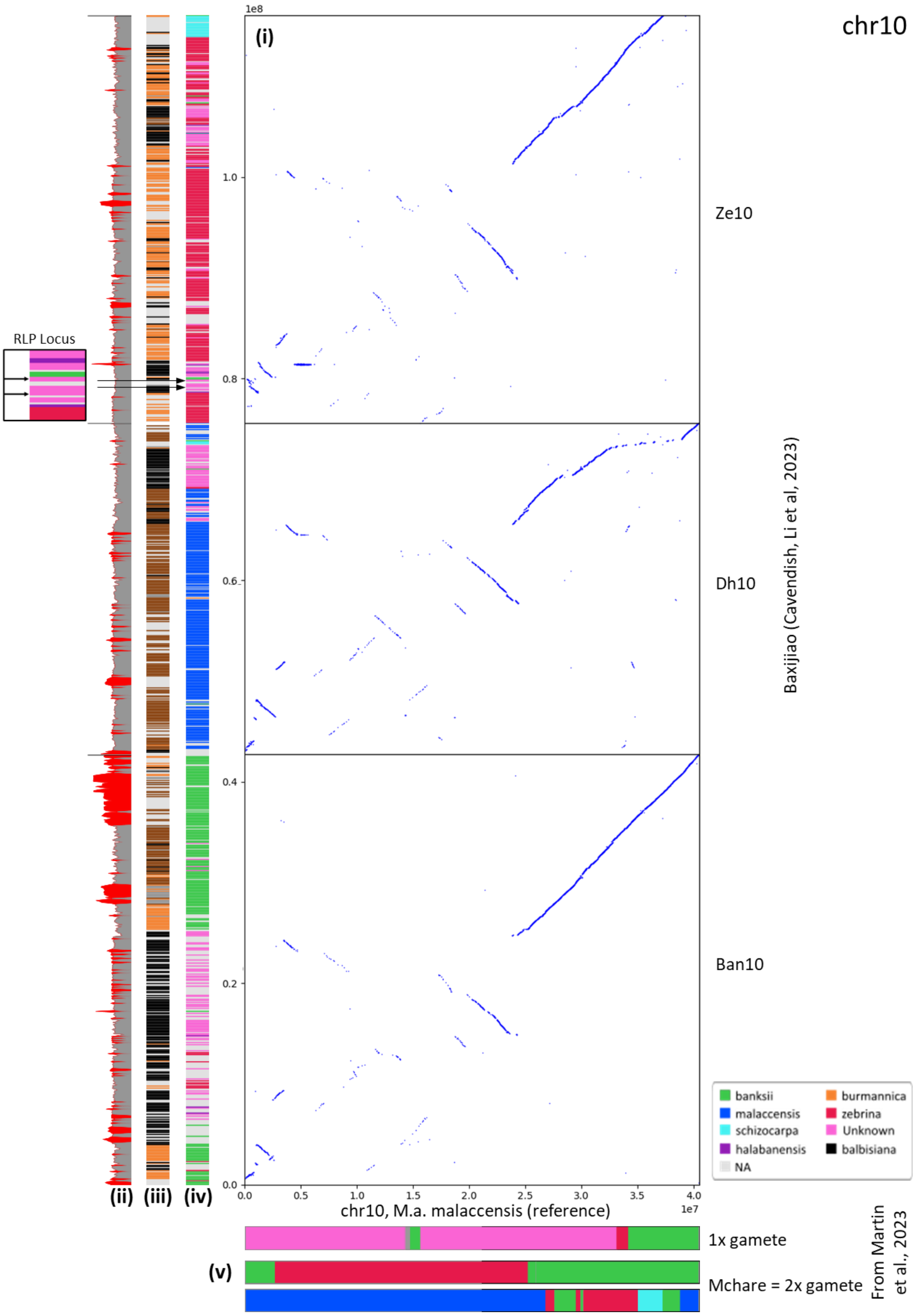

Supplementary Fig. 32. Assessment of Chromosome 10 of Cavendish assembly from Li *et al*. [6].
Same legend as Supplementary Fig. 23. Black arrows locate the RLP locus cluster suspected to be involved in Foc1 resistance[7] and show its unknown ancestral origin. A zoom was performed in the region to have a better view of the ancestral origin in the region. Source data are provided as a Source Data file.

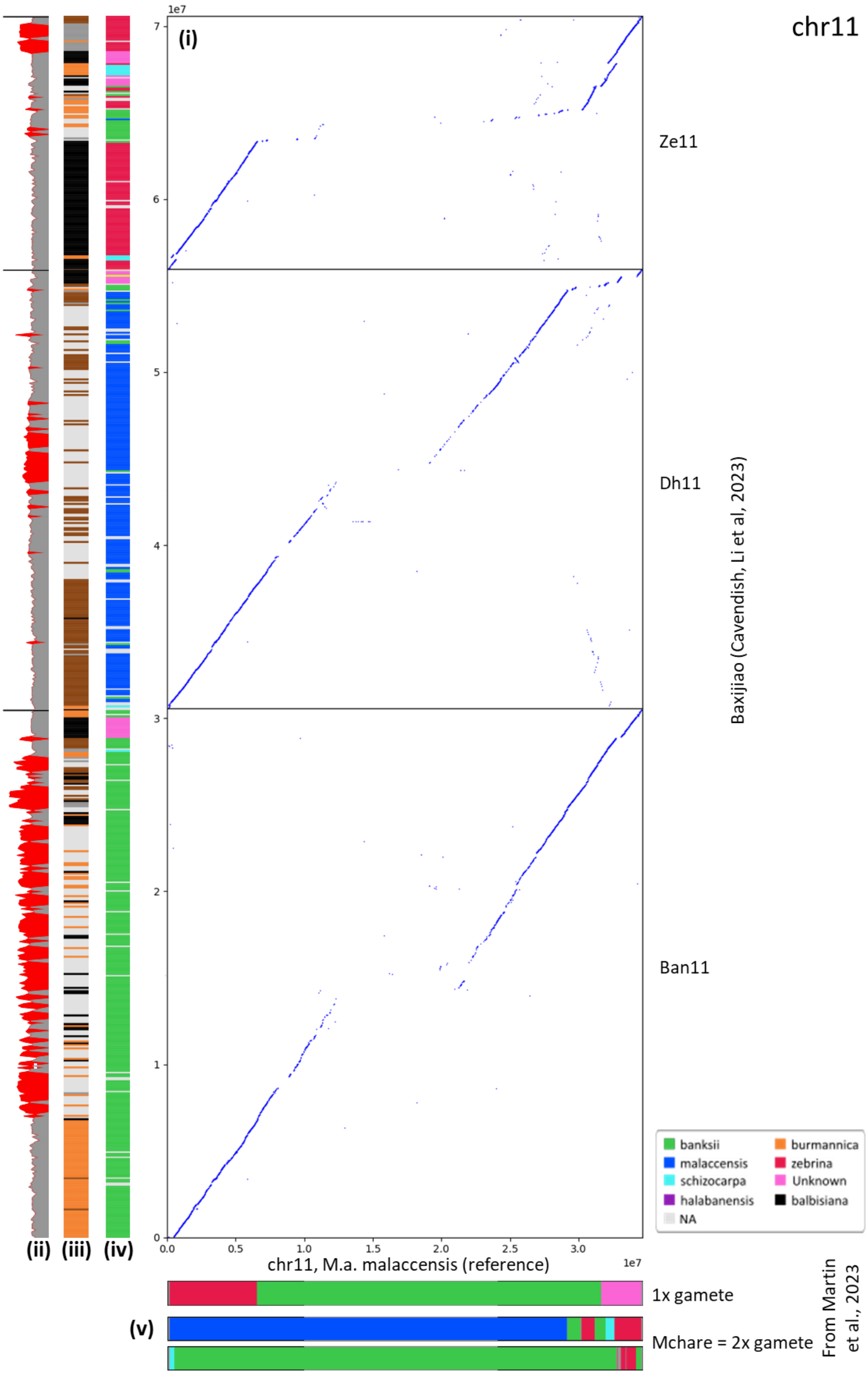

Supplementary Fig. 33. Assessment of Chromosome 11 of Cavendish assembly from Li *et al.* [6].
Cavendish assembly was evaluated using (i) a dot-plot comparison with the *M. a. malaccensis* reference assembly, (ii) Cavendish Illumina read coverage (SRR23425445) along the haplotype assembly, (iii) *in silico* chromosome painting based on hypothesised gametes haplotypes from Martin *et al*.[5] and (iv) *in silico* Cavendish assembly ancestry painting comparison to Cavendish mosaic painting of Martin *et al*.[6] (v). The average read coverage along assembly (ii) was calculated along chromosomes on sliding windows of 100kb. Overcovered regions, likely resulting from the collapse of two or more haplotypes or repeat regions, are distinguished from the normally covered regions (in grey) by a different red colour. In (iii), the black colour is for the 1x gamete and orange and brown for the two haplotypes of the 2x gamete. Regions in grey represent regions transmitted by the 2x gamete but for which it is not possible to determine whether they belong to haplotype 1 or 2 because of homozygosity between the two haplotypes. The legend for ancestry painting (iv and v) is located on the right bottom corner of each figure. Source data are provided as a Source Data file.

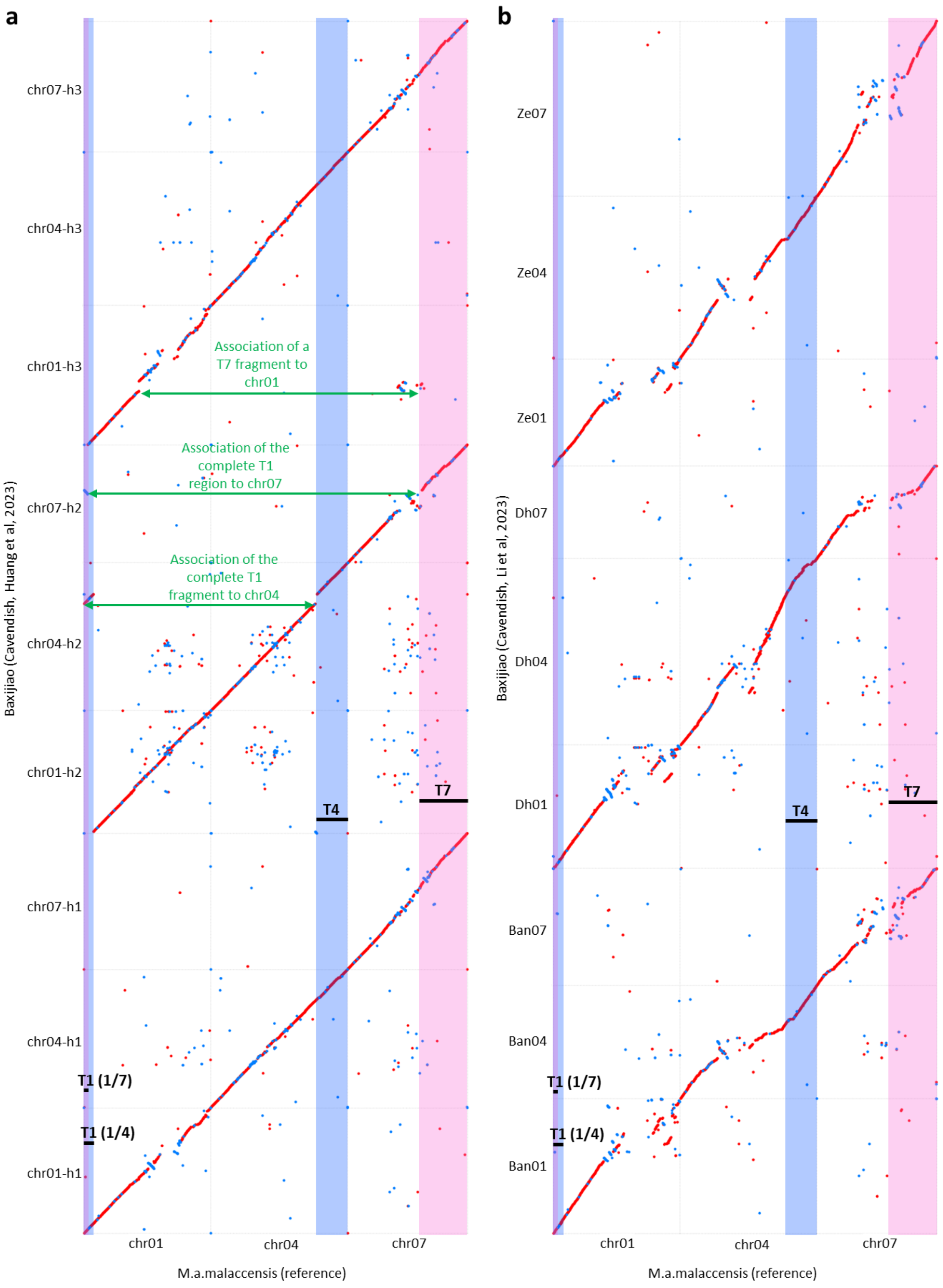

Supplementary Fig. 34. Dot-plot comparison of chromosomes 1, 4 and 7 between Cavendish assemblies and *M. a. malaccensis* reference assembly.

Dot plot comparison was performed between Cavendish assemblies from Huang *et al*.[4] (a) or Li *et al*.[7] (b) and *M. a. malaccensis* (reference) assemblies for chromosomes implicated in reciprocal translocations. Expected translocated regions are indicated by light blue and pink areas respectively for the translocations implicating chromosomes 1 and 4, 1 and 7. Translocated fragments are named T1 (1/4), T1 (1/7), T7, according to Martin *et al*.[3]. The green arrows locate expected translocation breakpoints that are present in the assembly. Source data are provided as a Source Data file.

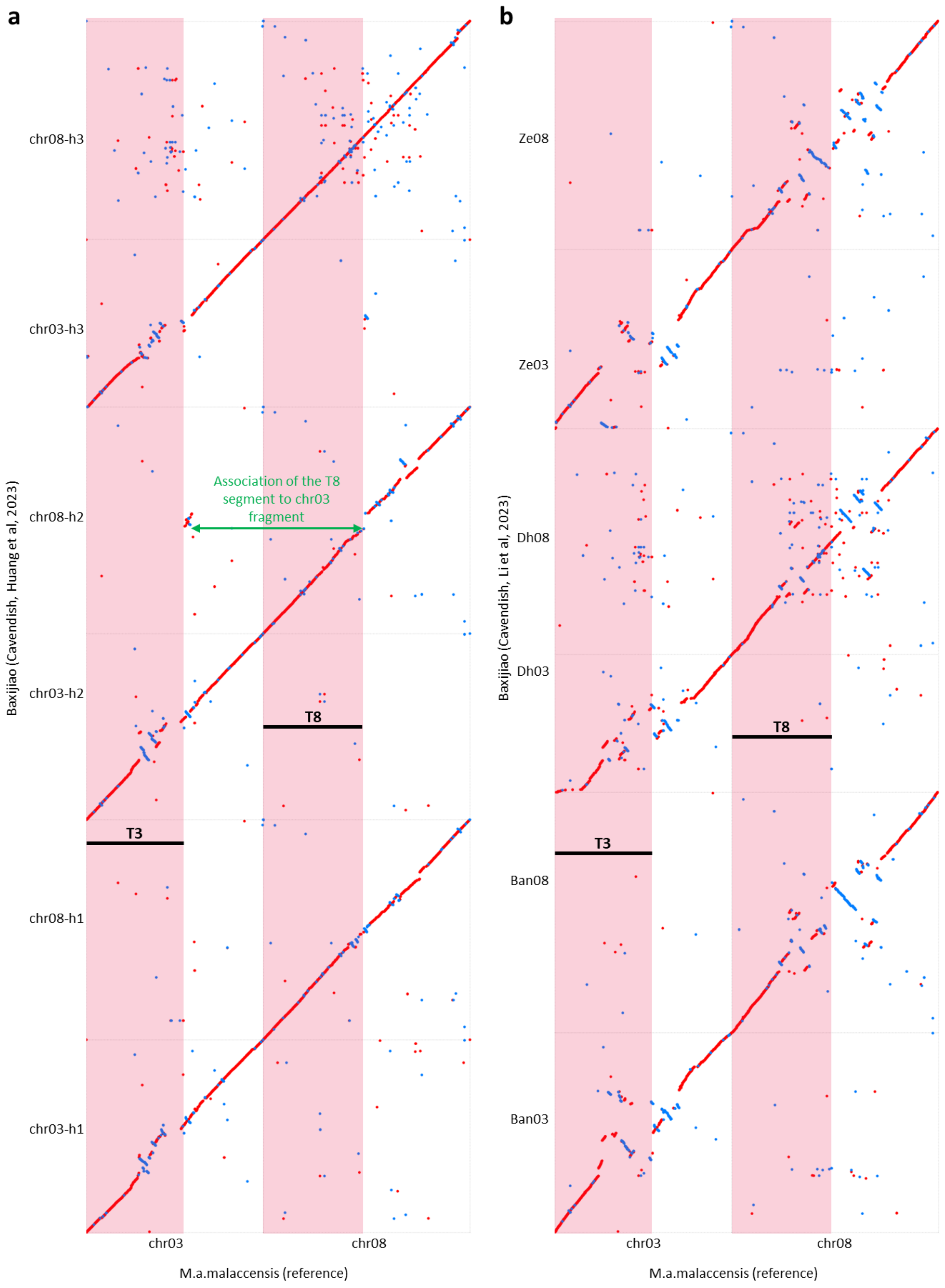

Supplementary Fig. 35. Dot-plot comparison of chromosomes 3 and 8 between Cavendish assemblies and *M. a. malaccensis* reference assembly.

Dot plot comparison was performed between Cavendish assemblies from Huang *et al.*[4] (a) or Li *et al.*[7] (b) and *M. a. malaccensis* (reference) assemblies for chromosomes implicated in reciprocal translocations. Expected translocated regions are indicated by light red areas for the translocations implicating chromosomes 3 and 8. Translocated fragments are named T3 and T8 according to Martin *et al.*[3]. The green arrows locate expected translocation breakpoints that are present in the assembly. Source data are provided as a Source Data file.

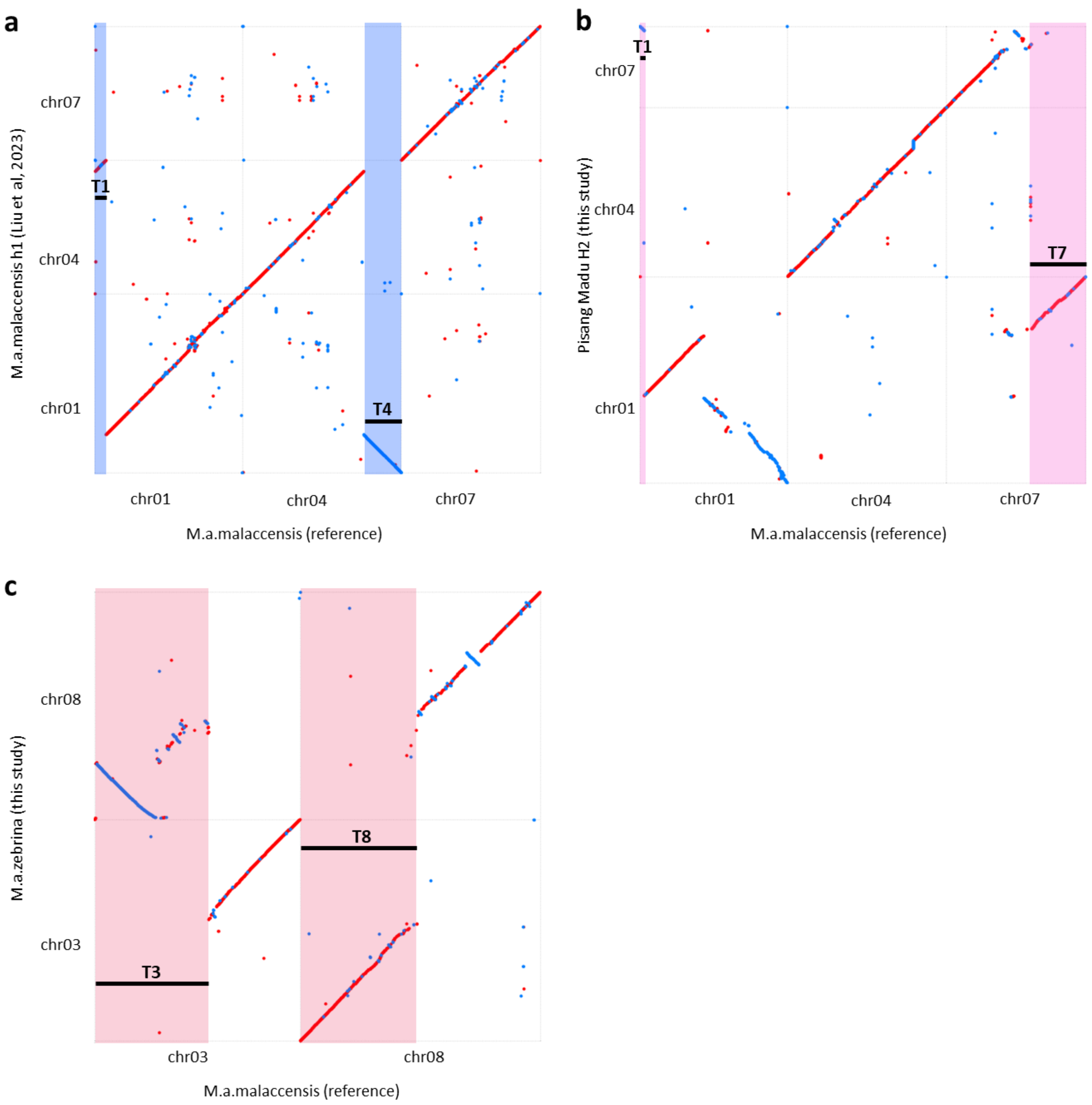

Supplementary Fig. 36. Dot-plot comparison of chromosomes implicated in translocations between *M. a. malaccensis h1, Pisang Madu H2,* and *M. a. zebrina* and *M. a. malaccensis* reference assembly.

Dot-plots between *M. a. malaccensis* (reference) assemblies[1] with *M. a. malaccensis h1*[8] (a), *Pisang Madu H2* (b) and *M. a. zebrina* (c) assemblies having respectively the 1/4, 1/7, and 3/8 translocated structures were also performed for comparison with Supplementary Fig. 34 and 35. Translocated regions are indicated by light blue, pink and red areas respectively for the translocations implicating chromosomes 1 and 4, 1 and 7 and 3 and 8. Translocated fragments are named T1 (1/4), T1 (1/7), T7, T3 and T8 according to Martin *et al*.[3]. Source data are provided as a Source Data file.

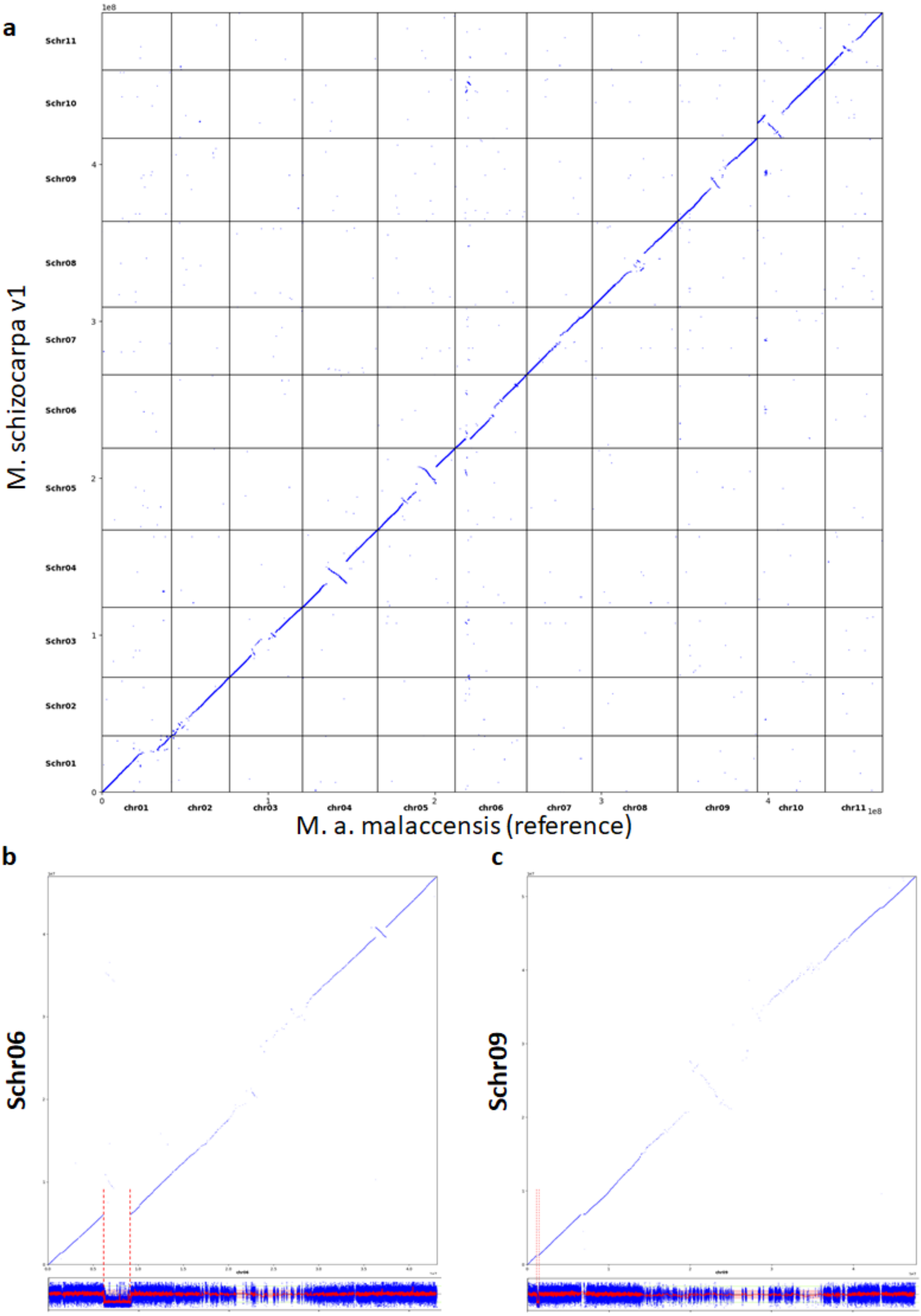

Supplementary Fig. 37. Dot plot and read coverage highlighting problems in the first version of the *M. schizocarpa* assembly.

(a) Dotplot comparison of *M. schizocarpa* assembly[9] to the last version of the *M. a. malaccensis* reference sequence, DH-Pahang v4[10] revealed a global synteny except in centromeric regions. It also revealed two large regions on chromosomes 6 (b) and 9 (c), located by red vertical dashed lines, which are present in the *M. a. malaccensis* reference sequence but are absent in *M. schizocarpa* assembly[9]. The coverage density of *M. schizocarpa* short reads along the *M. a. malaccensis* reference sequence (clouds of blue dots and the red curve representing the average read coverage on sliding windows of 400 positions at the bottom of under figure b and c) is about half of the other segments in these regions suggesting that only one haplotype of *M. schizocarpa* contains the sequence. Source data are provided as a Source Data file.

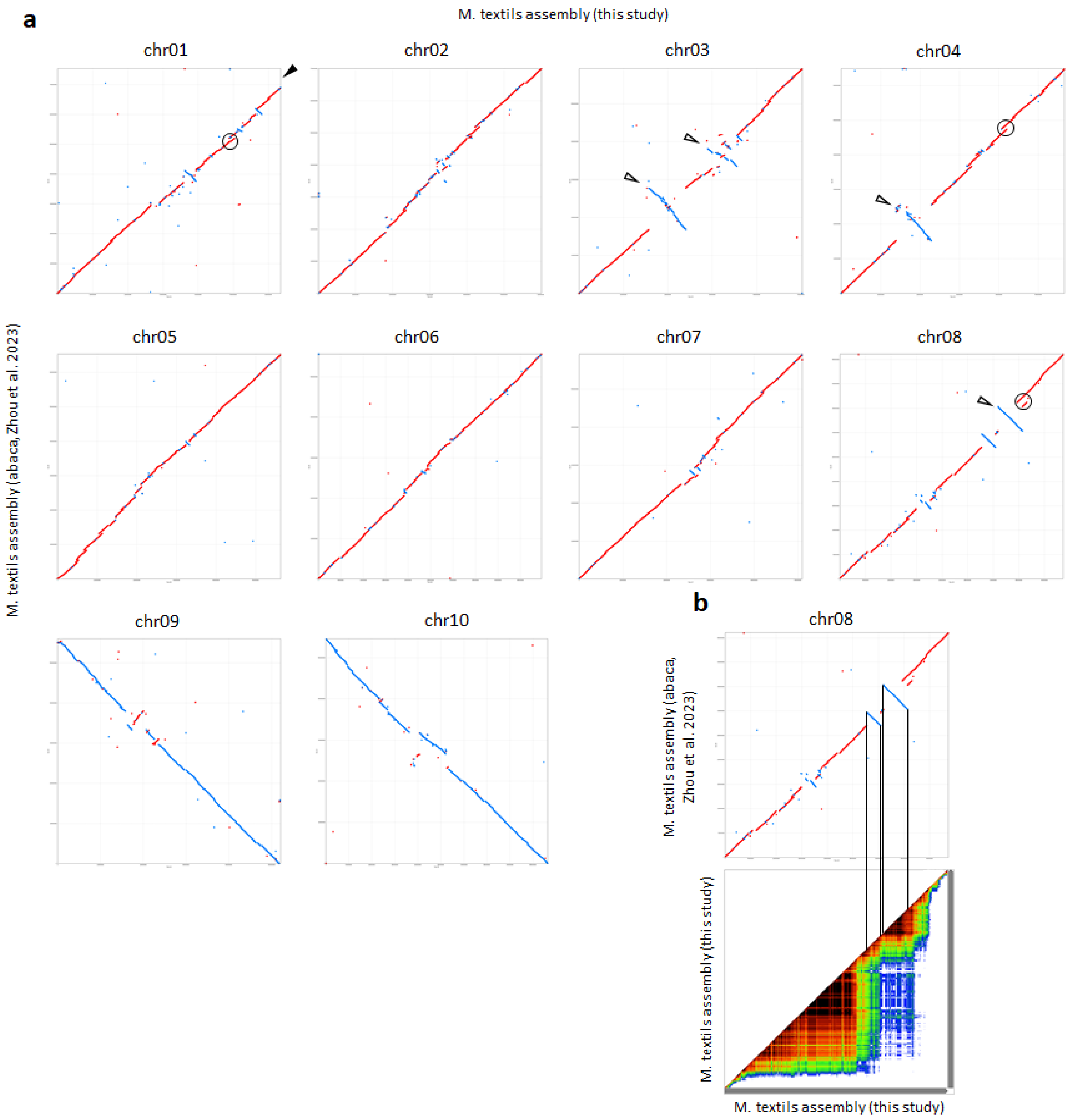

Supplementary Fig. 38. Comparison of *M. textilis* assemblies.
Chromosome comparison between *M. textilis* assemblies from this study (x-axis) and from Zhou *et al.*[11] (y-axis). The black arrowhead indicates the *M. textilis* missing fragment in the assembly from this study, open arrowheads indicate large centromeric inversions between assemblies and open circles indicate duplications in the Zhou *et al.*[11] genome. b) Example of pairwise genetic distances for chromosome 08 markers plotted along chromosome 08 of *M. textilis* assembly from this study and validating the structure proposed relative to the structure proposed by Zhou *et al.*[11] assembly. Pairwise genetic distances are calculated from GBS data of the AFTXT population (Supplementary Data 5). Source data are provided as a Source Data file.

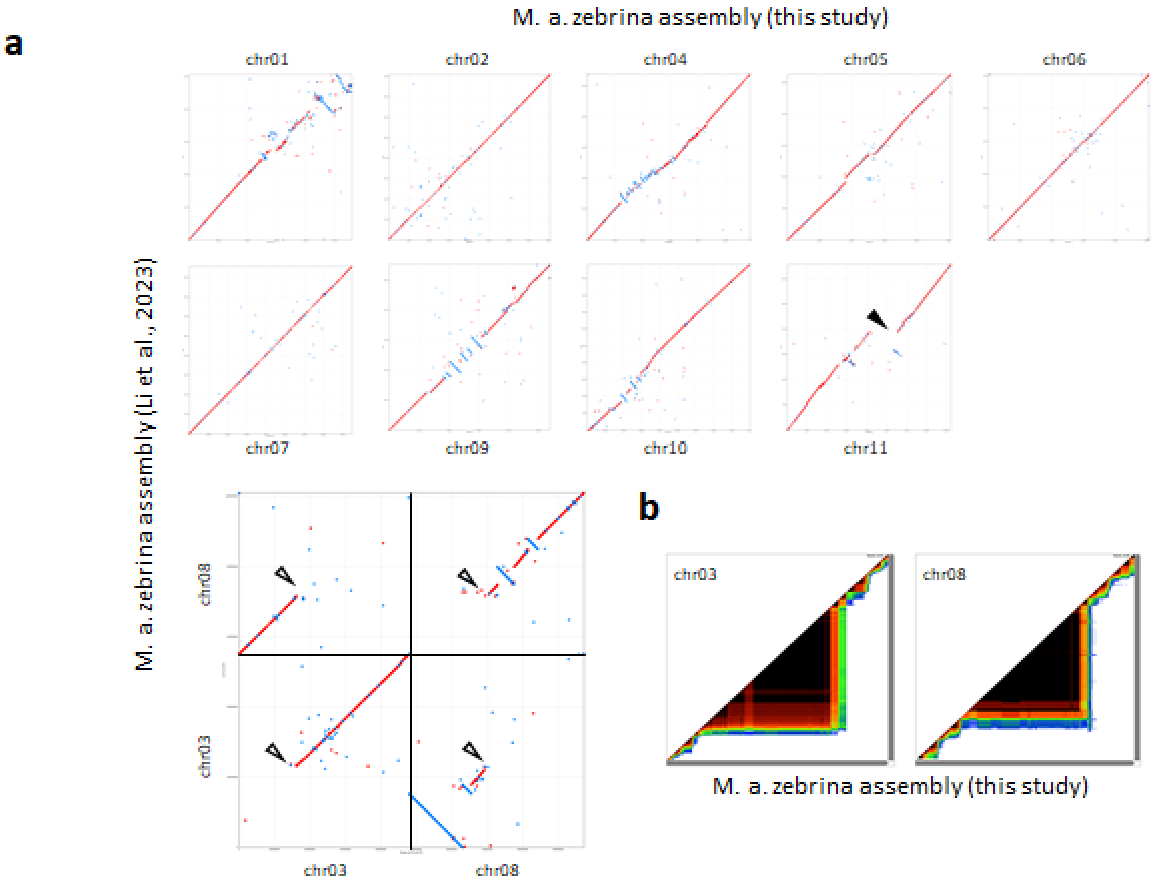

Supplementary Fig. 39. Comparison of *M. a. zebrina* assemblies.
(a) Chromosome comparison between *M. a. zebrina* assemblies from this study (x-axis) and from Li *et al.*[7] (y-axis). Arrowheads indicate synteny break with loss of sequence (black arrows) and reciprocal translocation (open arrows). Chromosomes 03 and 08 have been concatenated to highlight differences between the assemblies in relation with the reciprocal translocation. (b) Pairwise genetic distances for chromosome 03 and 08 markers plotted along chromosome 03 and 08 of *M. a. zebrina* assembly from this study and validating the proposed structure. Pairwise genetic distances are calculated from GBS data of the PCMo population (Supplementary Data 5). Source data are provided as a Source Data file.

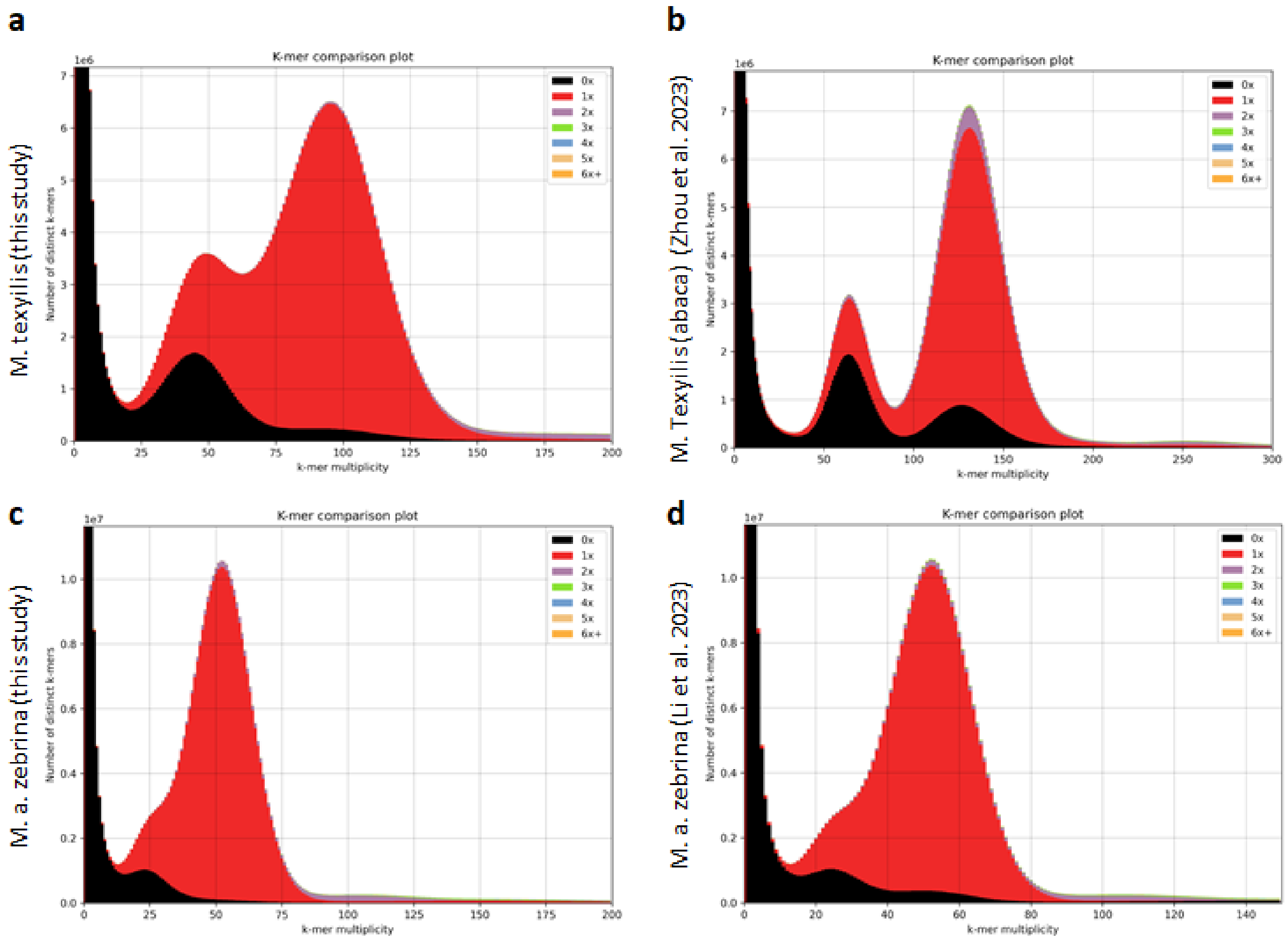

Supplementary Fig. 40. KAT plots of Musa assemblies.

KAT plots were calculated on the assemblies in this study for which a high-quality assembly of the same species is already available: i.e. *M. textilis* (a) and *M. a. zebrina* (c). For comparison, KAT plots were also calculated on these already available assemblies: (b) *M. textilis* (abaca)[11] and (d) *M. a. zebrina*[7]. KAT plots were calculated using Illumina sequencing data of corresponding assemblies. Source data are provided as a Source Data file.

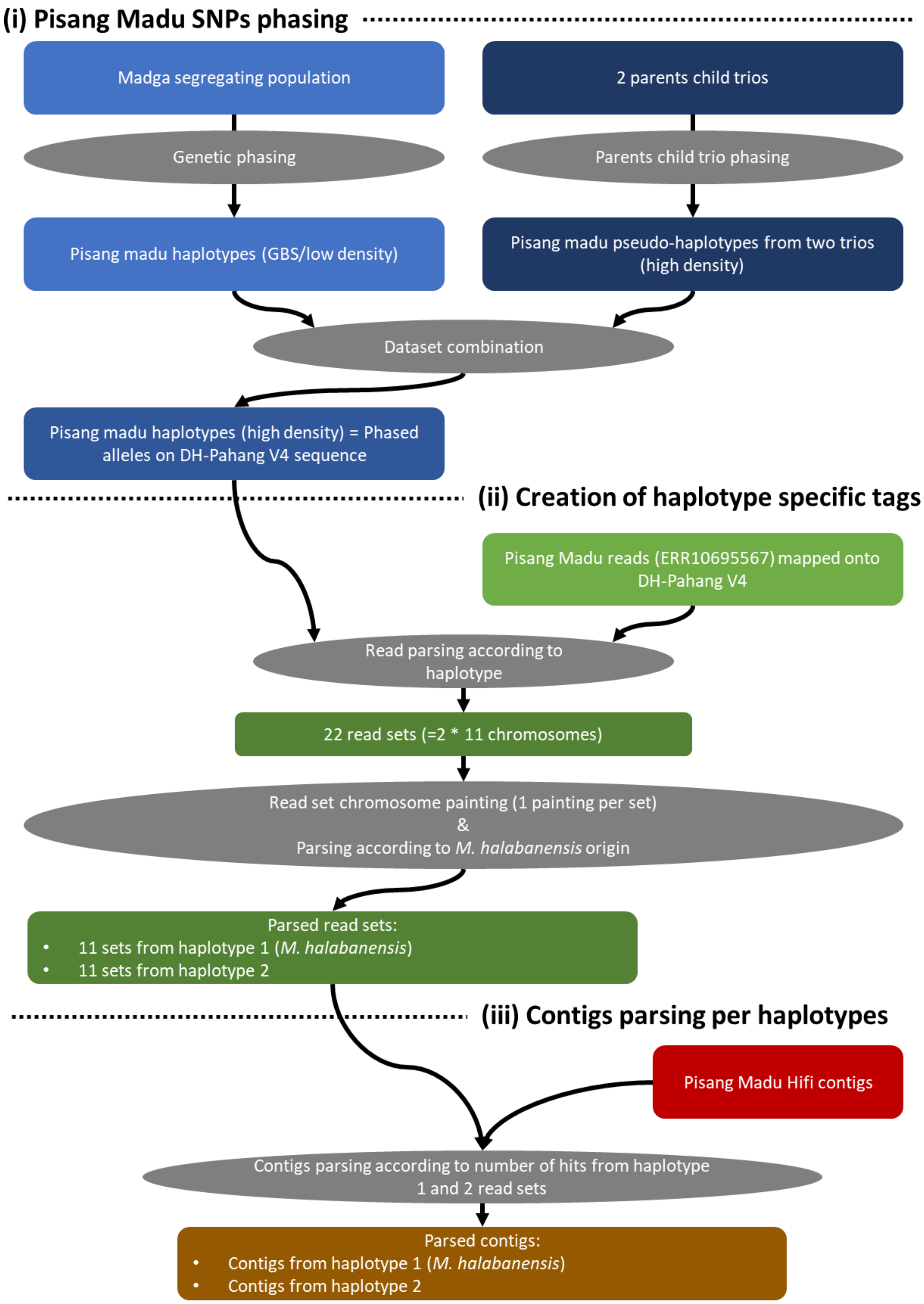

Supplementary Fig. 41. Workflow describing the process to parse contigs from Pisang Madu haplotype assembly.

Genotyping by sequencing data from a bi-parental population implicating Pisang Madu as one parent and Illumina whole genome sequencing of two parents-child trios with Pisang Madu as parent were used (i) to phase SNPs from Pisang Madu, (ii) create tags specific of each Pisang Madu haplotypes that were used to (iii) parse Pisang Madu contigs into two haplotypes.

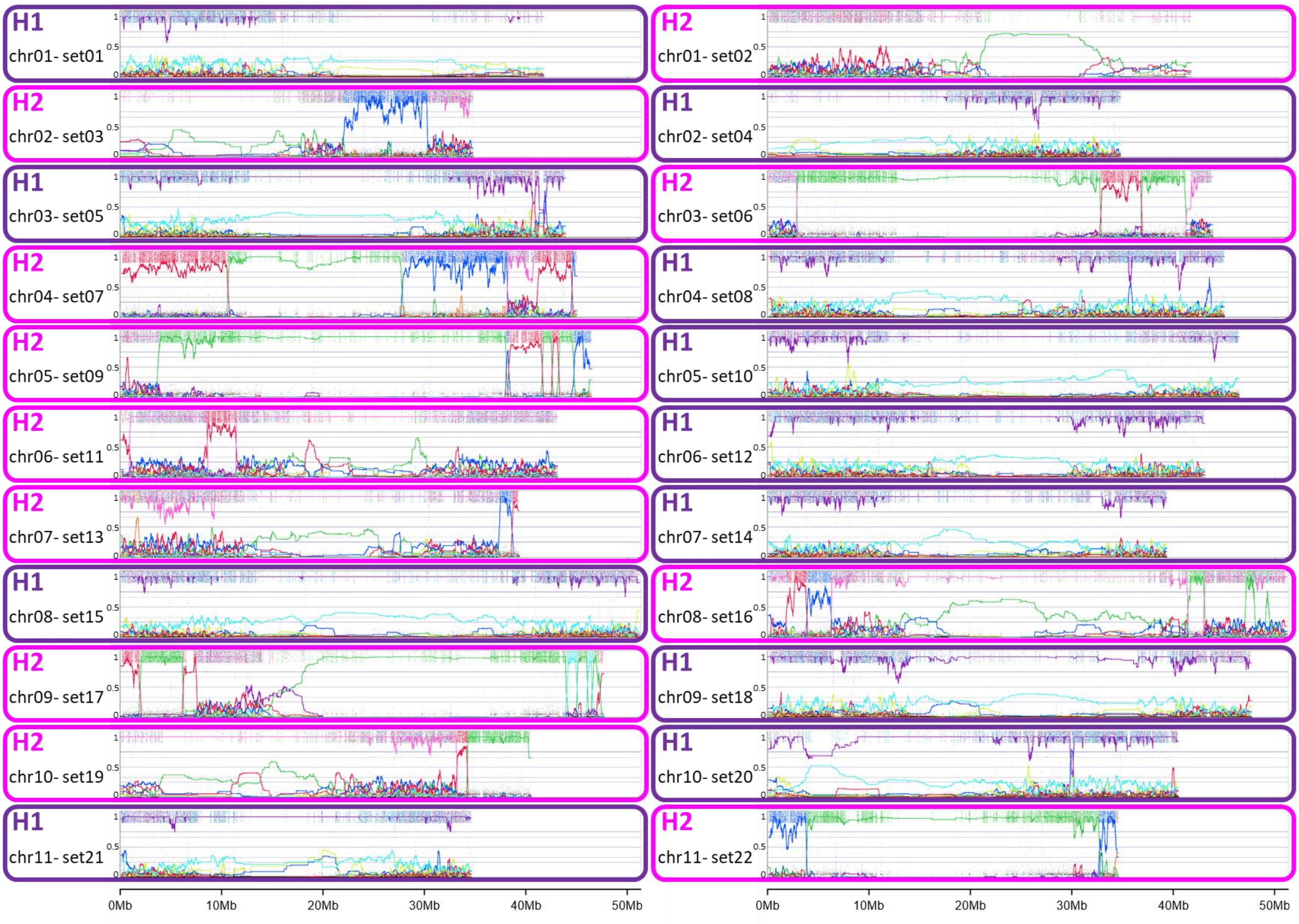

Supplementary Fig. 42. Normalized curve ratio for each read set of Pisang Madu.
Source data are provided as a Source Data file.

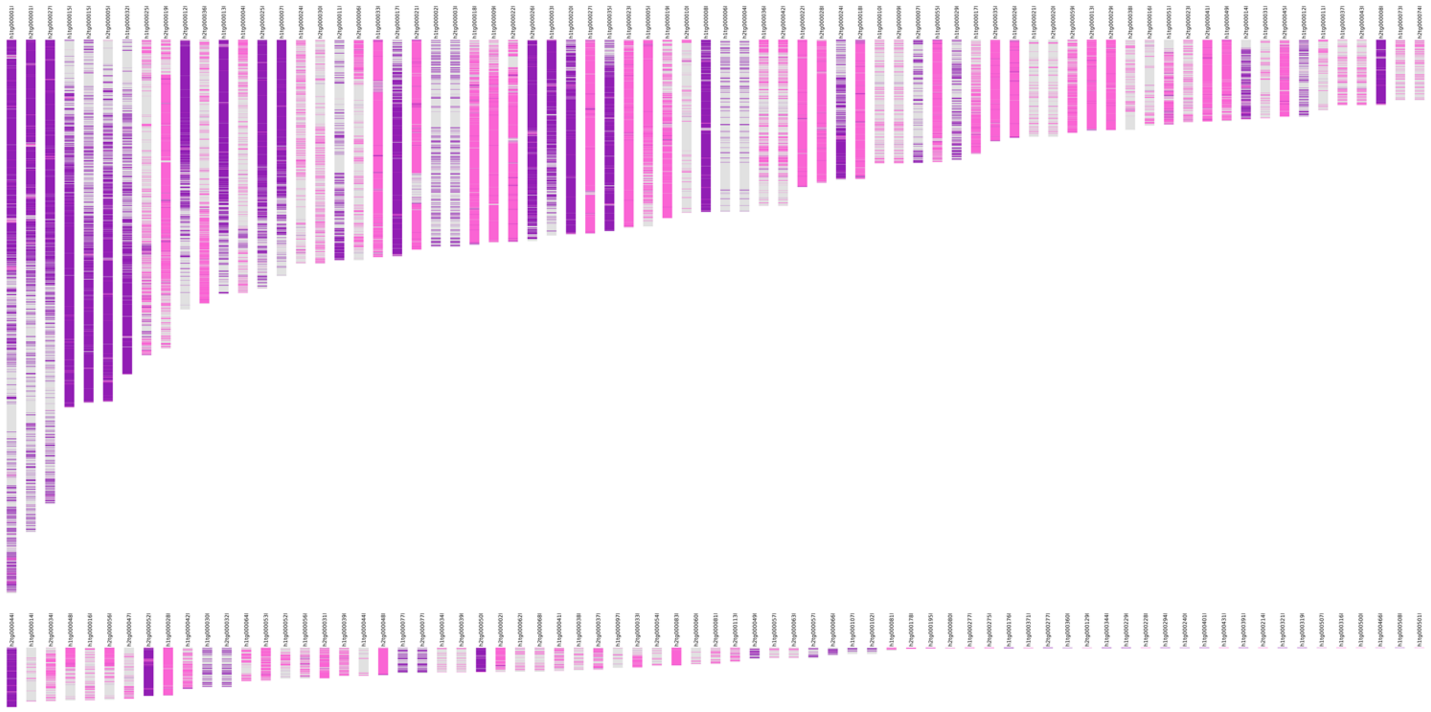

Supplementary Fig. 43. Pisang Madu contigs chimerism validation and attribution to haplotypes.
Pisang Madu contigs painting using Pisang Madu haplotype specific read sets previously defined. Painting was obtained by mapping haplotype specific reads set along each contigs independently, selecting only perfect match and attributing a color (haplotype 1 = purple, haplotype2 = pink) based on a majority rule on a sliding window of 10kb. Source data are provided as a Source Data file.

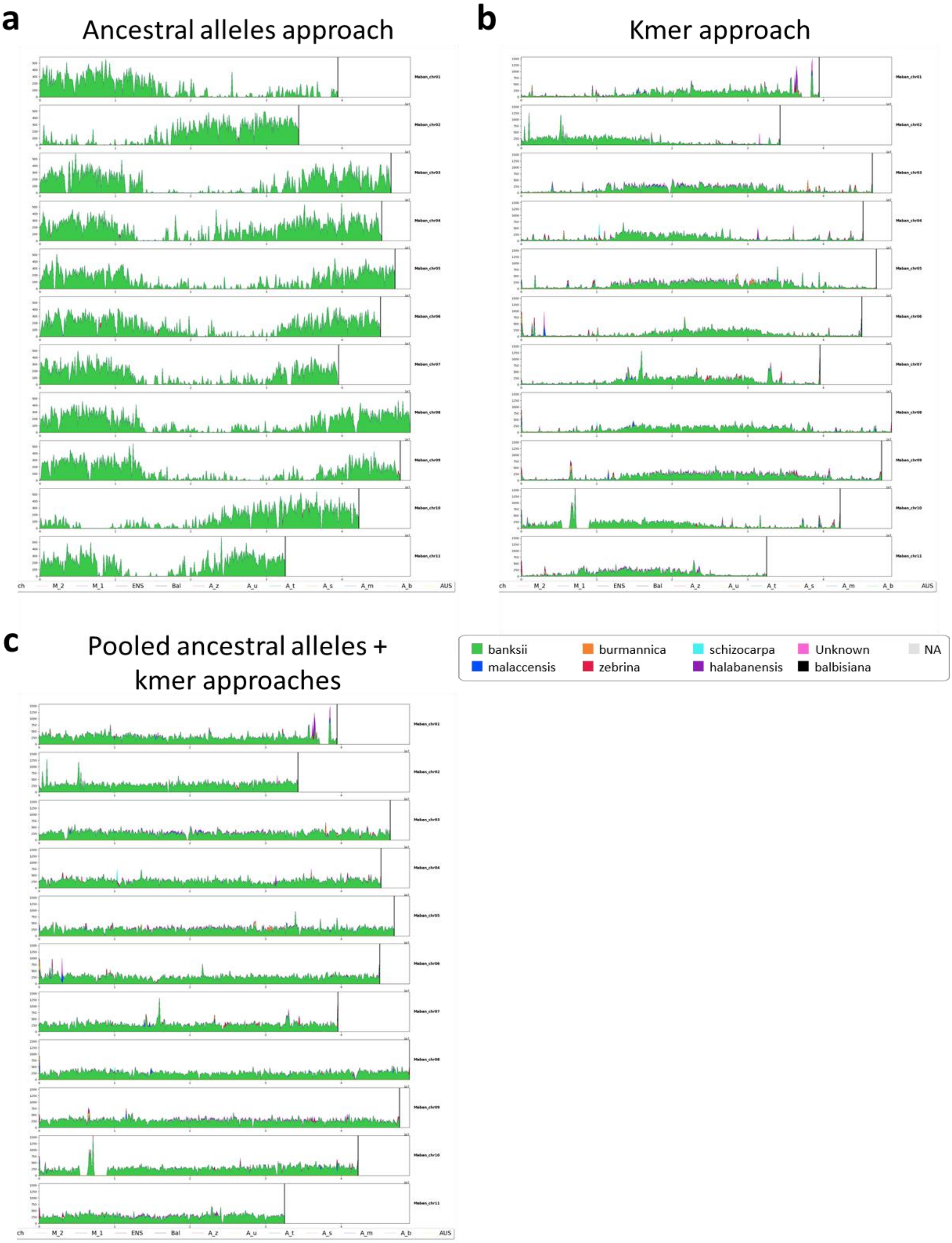

Supplementary Fig. 44. Example of the number of perfect alignments of each ancestral origin tags along the *M. a. banksii* assembly.

The number of ancestral origin tags generated using the ancestry specific SNPs identified in Martin *et al*.[6] (a), the k-mer in repeats portion (b) and a pooled of both tags (c) is displayed along the chromosomes of *M. a. banksii* assembly. In (a) the tag sampling is biased towards over-representing chromosome arms; in (b) the tag sampling is biased towards over-representing pericentromeric regions; in (c) the merging of both samples displays a homogeneous tag sampling along chromosomes. Satisfyingly, the vast majority of tags are from the green origin (representing the *M. a. banksii* genetic pool) which was expected for the *M. a. banksii* assembly. Source data are provided as a Source Data file.

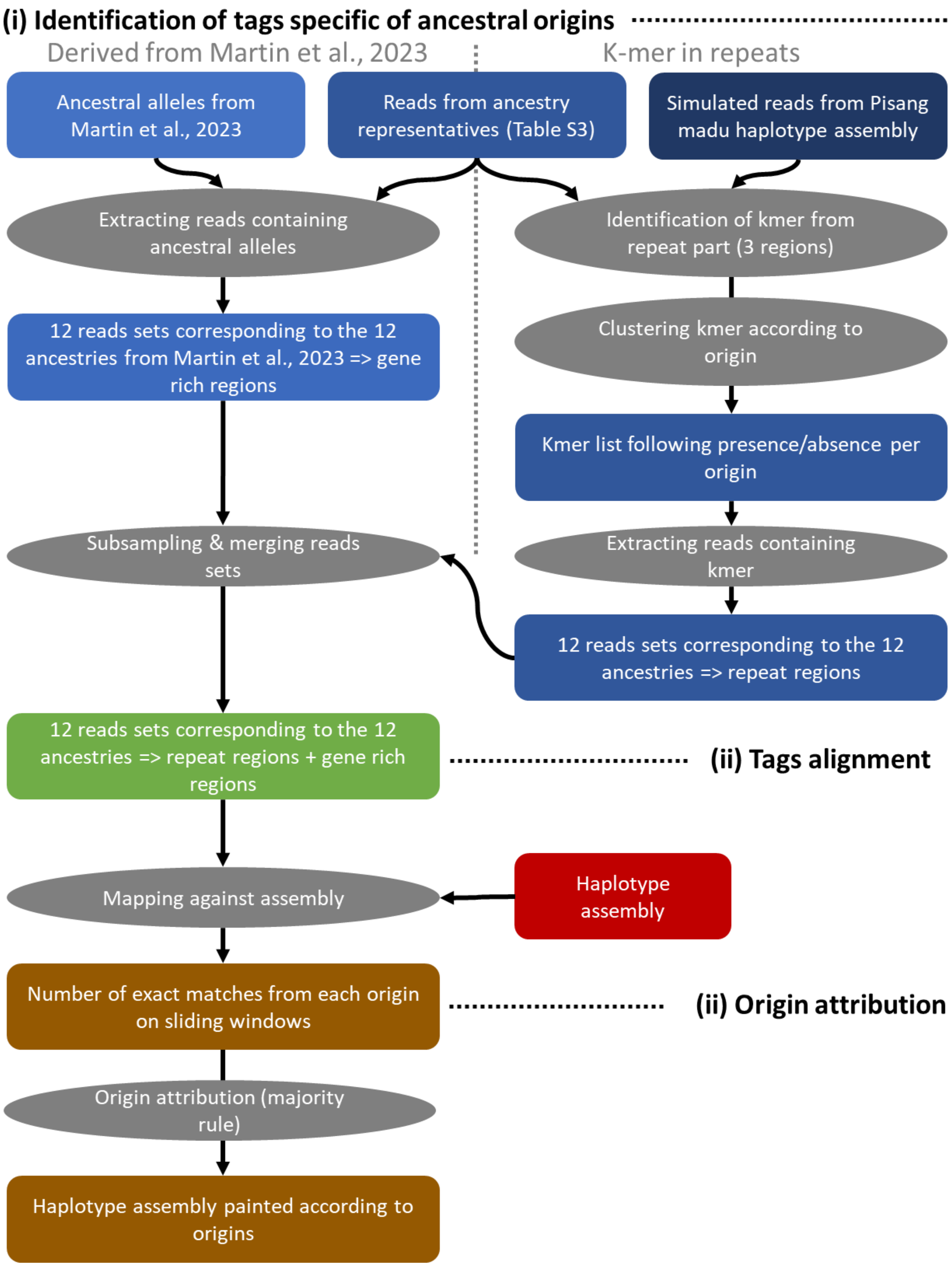

Supplementary Fig. 45. Workflow describing the process to perform ancestry painting of assemblies. Sequencing material from banana ancestry representative individuals associated to ancestry specific SNPs from Martin *et al.*[6] were used to perform ancestry chromosome painting of assemblies by (i) generating tags specific of each ancestral origin, (ii) aligning these tags against an assembly and (iii) attribute an ancestral origin along chromosomes using a sliding window.

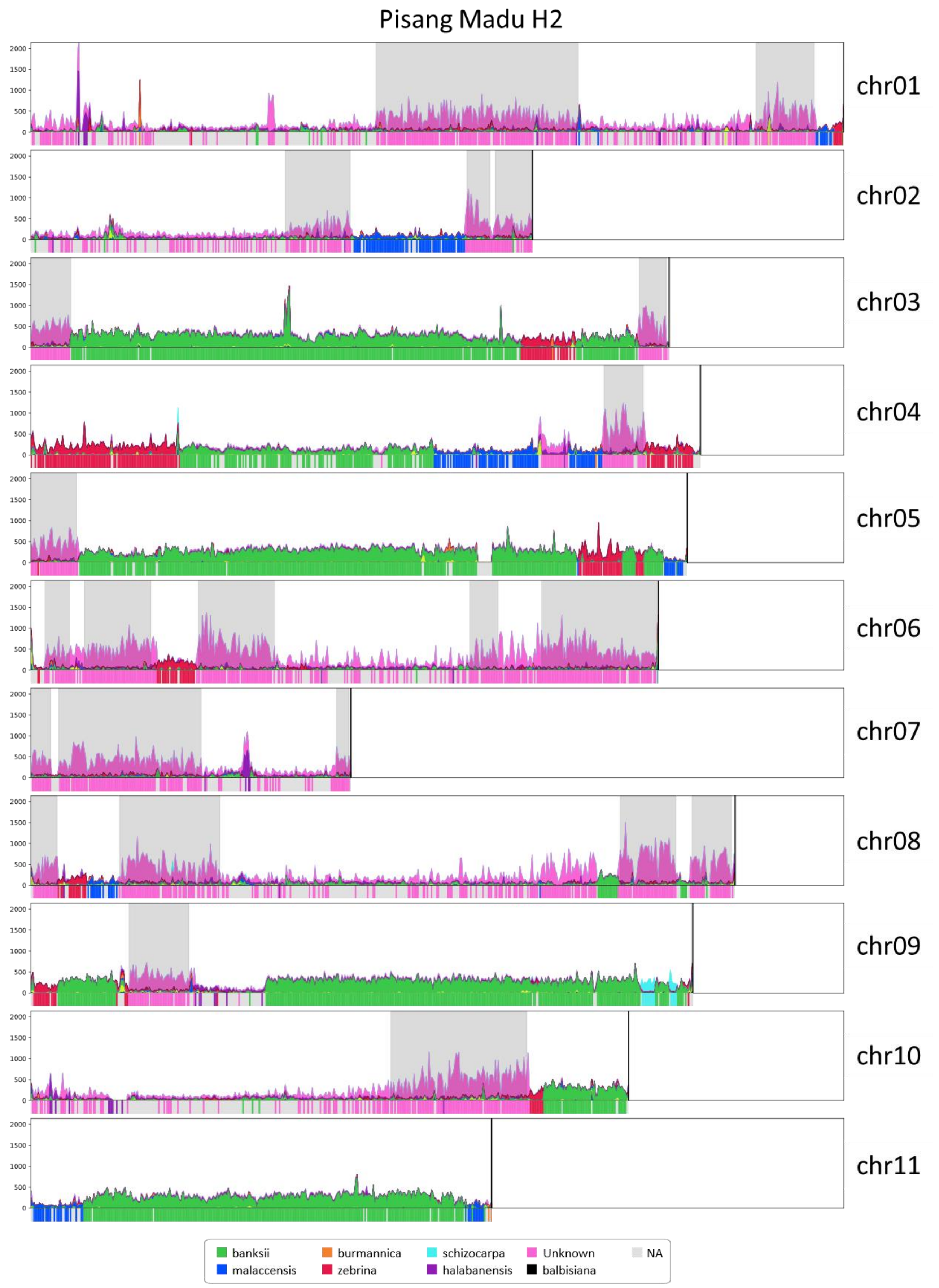

Supplementary Fig. 46. Regions identified as unknown ancestral origin in Pisang Madu H2 assembly. Curves represent the number of perfect alignments of ancestral origin tags along the assembly of Pisang Madu H2 and the inferred ancestry painting of the assembly is drawn under the curves. Grey shaded regions indicate regions of unknown origin containing genes that were used for the phylogenetic analysis. Source data are provided as a Source Data file.

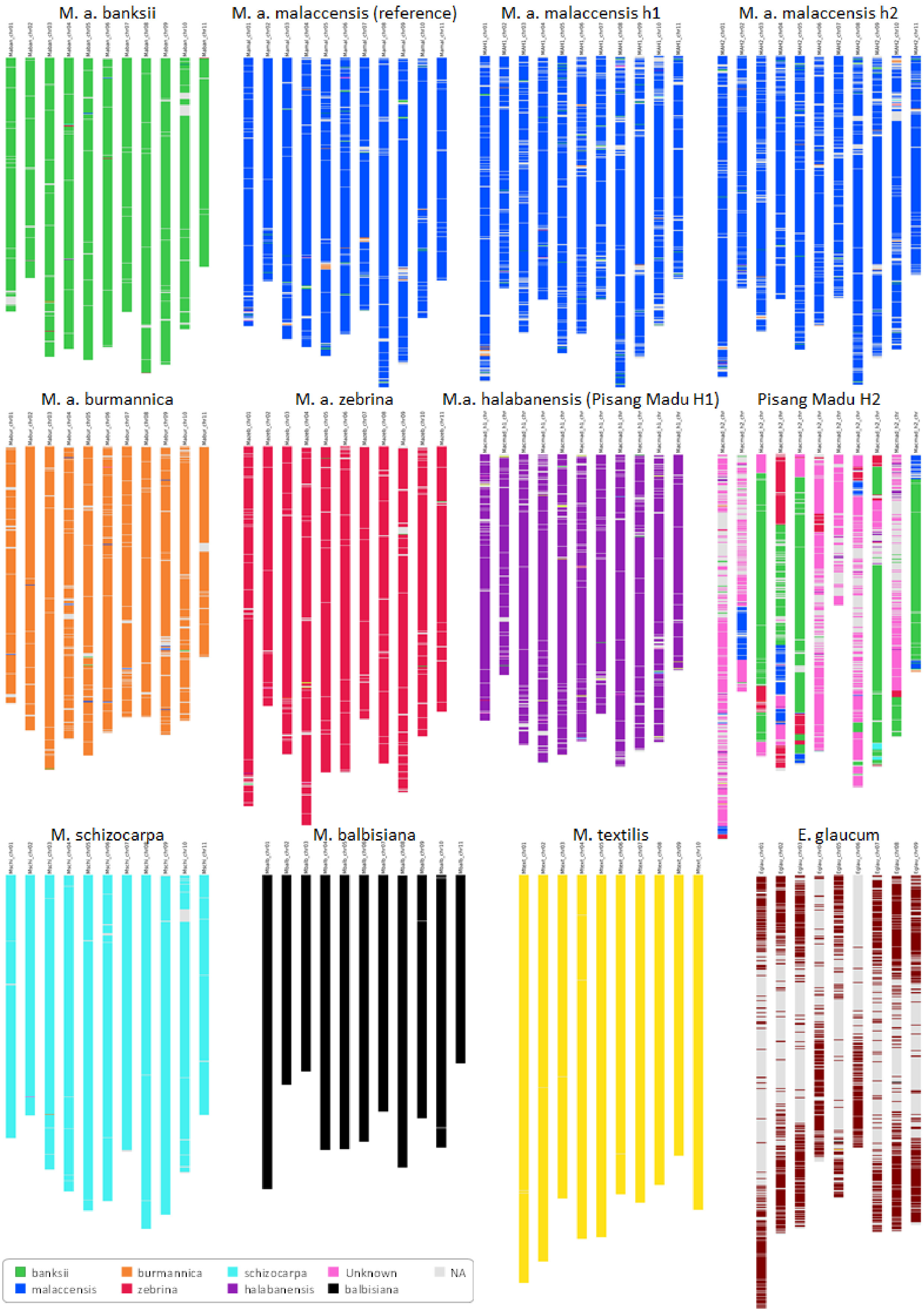

Supplementary Fig. 47. Chromosome ancestry painting of assemblies used in the phylogenetic analysis. Chromosomes are ordered by increasing numbers. Colour codes indicate the involved ancestors as defined by Martin *et al*.[6]. 'Unknown' is for the remaining unknown ancestry and 'NA' corresponds to regions in which no origin could be attributed. Source data are provided as a Source Data file.

Supplementary Note 1. Comparison with existing assemblies

Our assemblies of *M. textilis* and *M. a. zebrina* were compared (assembly metrics, dotplot and Kat-plots) with previous chromosome scale assemblies from these two taxons i.e. *M. textilis*[11] and *M. a. zebrina*[7]. Previous assemblies of *M. a. banksii* and *M. a. burmannica* from Rouard *et al.*[12] were preliminary draft assemblies and thus were not compared with our chromosome scale assemblies.

***M. textilis* assemblies**

The *M. textilis* assembly from Zhou *et al.*[11] is larger in size (by 67Mb) than our assembly Supplementary Data 7. Merqury scores and N50 metrics are lower in the *M. textilis* assembly from Zhou *et al.*[11] while their BUSCO metrics are higher, except for the BUSCO duplication metrics. Global synteny comparisons performed between the two *M. textilis* assemblies revealed a globally well conserved synteny (Supplementary Fig. 38). Some differences can be observed, in particular (i) a large region of around 6 Mb in size that is missing at the end of chromosome 1 in the assembly from our study (ii) some duplicated segments present in almost all chromosomes of *M. textilis* assembly from Zhou *et al.*[11] resulting probably from a stacking of the two haplotypes in heterozygous regions rather than merging both haplotypes in a consensus and (iii) inversions in centromeric regions that tend to be validated in our assembly by genetic mapping data, even if the reduced recombination in these regions makes it difficult to draw definitive conclusions. KAT plots revealed a heterozygous genome for both sequenced individuals (Supplementary Fig. 40 a and b); however, the *M. textilis* assembly from Zhou *et al.*[11] includes allelic duplications (violet curve) and higher proportion of missing k-mers (shown by the black curve in the second peak). Therefore, the difference of genome assembly size may be partly explained by a missing region at the end of chromosome 1 in our assembly and by the presence of artificial allelic duplications in the assembly of Zhou *et al.*[11].

***M. a. zebrina* assemblies**

Our assembly of *M. a. zebrina* and the one from Li *et al.*[7] have very similar assembly metrics (Supplementary Data 7). Dot plot analysis showed that although the synteny is globally conserved, there are (i) a few inversions in pericentromeric zones, (ii) a large missing region on chromosome 11 in the assembly of Li *et al.*[7] and (iii) a reciprocal translocation between chromosomes 3 and 8 in our assembly (Supplementary Fig. 39). Li *et al.*[7] guided their assembly with the published DH-Pahang assembly and thus kept the chromosomal structure of DH-pahang and so this assembly does not display the 3/8 translocation although all the *M. a. zebrina* accessions analyzed so far displayed this translocation[13]. In addition, genetic mapping data confirms the structure proposed by our assembly in this study. KAT plots revealed a relatively homozygous genome for both sequenced individuals and few allelic duplications of similar order of magnitude (Supplementary Fig. 40 c and d); however, the assembly of Li *et al.*[7] showed a higher proportion of missing k-mers compared to our assembly (shown by the black curve in the second peak). Globally, both assemblies are similar in content but our assembly displays the 3/8 translocation (between reference chromosomes 3 and 8) that has been observed in all *M. a. zebrina* so far.

Supplementary Method 1. Sequencing data and chromosome assembly

Illumina PCR-Free library preparation and sequencing

A PCR free library was prepared for each sample using the Kapa Hyper Prep Kit (Roche, Basel, Switzerland). Genomic DNA (1.5 to 3µg) was sonicated using a Covaris E220 sonicator (Covaris, Woburn, MA, USA). Fragments (1.1 to 1.6µg) were end-repaired, 3′-adenylated and Illumina adapters (Nextflex™ PCR Free Barcodes or NEXTflex HT™ Barcodes (Perkin Elmer, Waltham, MA, USA)) were ligated according to the manufacturer's instructions. Ligation products were purified twice with AMPure XP beads (Beckman Coulter Genomics, Danvers, MA, USA) and quantified by qPCR (MxPro, Agilent Technologies, Santa Clara, CA, USA) using the KAPA Library Quantification Kit for Illumina Libraries (Roche). Libraries were sequenced on a paired-end mode with an Illumina HiSeq 2500 instrument (Illumina, San Diego, CA, USA) using 251 base-length read chemistry.

After the Illumina sequencing, an in-house quality control process was applied to the reads that passed the Illumina quality filters. The first step discards low-quality nucleotides (Q < 20) from both ends of the reads. Next, Illumina sequencing adapters and primer sequences were removed from the reads. Then, reads shorter than 30 nucleotides after trimming were discarded. These trimming and removal steps were achieved using in-house-designed software based on the FastX package39. The last step identifies and discards read pairs that are mapped to the phage phiX genome, using SOAP aligner40 and the Enterobacteria phage PhiX174 reference sequence (GenBank: NC_001422.1). This processing, described in Alberti *et al.*[14], resulted in high-quality data.

PromethION library preparation and sequencing

Libraries were mainly prepared by ligation following the protocols provided by Oxford Nanopore with some exceptions: incubation times of the End Prep step were increased from 5 to 20 minutes, the ligation temperature was 25°C instead of room temperature, all the incubation times during the beads purification steps were increased from 5 to 10 minutes and the final elution was performed at 37°C (Supplementary Data 2).

M. a. burmannica (selfing of PT-BA-00051), *M. a. zebrina* (selfing of PT-BA-00182), *M. textilis* (PT-BA-00228): Genomic DNA was fragmented using the Megaruptor 2 (Diagenode, Seraing, Belgium), then the longest DNA fragments were size selected using a Blue Pippin instrument (Sage Science, Beverly, MA, USA). The library was prepared using the SQK-LSK108 kit (Oxford Nanopore Technologies Ltd, Oxford, UK) and sequenced with R9.4 Flow cells. Some samples were not fragmented and directly size selected before the library preparation.

M. schizocarpa (ITC0926): Libraries were prepared with the LSK-SQK109 kit (ONT) and sequenced with PromethION R9.4 flow cells.

M. a. banksii (PT-BA-00024): Libraries were prepared with the SQK-LSK108 or SQK-LSK109 kits (ONT) and sequenced with MinION or PromethION R9.4 flow cells.

Optical mapping for *M. a. banksii*

The Direct label and stain (DLS) labelling (using the DLE-1 enzyme) and the Nick Label Repair and Stain (NLRS) labelling (using the BspQI enzyme) protocols were performed according to Bionano Genomics with 750ng and 600ng of DNA respectively. The Chip loadings were performed as recommended by Bionano Genomics (Supplementary Data 2).

Hi-C library preparation and sequencing

Dovetail Omni-C library (Dovetail Genomics, Scotts Valley, CA, USA) was prepared from 300mg of flash-frozen pseudo-stem leaves, following the Non-Mammalian Samples Protocol (version 1.0). Briefly, chromatin was fixed with formaldehyde, randomly digested with DNase I and then extracted. Chromatin ends were repaired and ligated to a biotinylated bridge adapter, followed by proximity-ligation of adapter-containing ends. After proximity ligation, crosslinks were reversed and DNA was purified. Purified DNA was treated to remove biotin that was not internal to ligated fragments, and a sequencing library was generated using NEBNext Ultra enzymes and Illumina-compatible adapters. Biotin-containing fragments were isolated using streptavidin beads before PCR enrichment of the library. The Dovetail Hi-C library was then quantified by qPCR using the KAPA Library Quantification Kit for Illumina Libraries (KapaBiosystems), and the library profile was assessed using a High Sensitivity DNA kit on an Agilent Bioanalyzer

(Agilent Technologies, Santa Clara, CA, USA). The library was sequenced on an Illumina NovaSeq 6000 instrument (Illumina, San Diego, CA, USA) using 150 base length read chemistry in paired-end mode (Supplementary Data 2).

Long reads-based genome assembly

As this study lasted several years and the different genomes were sequenced during its whole life and not at the same time, different assembly strategies were used. These strategies reflect what we think were the best practices at the time.

The genome of *M. a. banksii* was assembled by giving the whole dataset of Nanopore reads (not cleaned) as input to the SMARTdenovo assembler (Github commit 5cc1356) with default parameters, except '-k 17' and '-c 1' to generate a consensus sequence. The assembly was then polished three times using Racon (version 1.2.1) with Nanopore reads and three times using Pilon (version 1.22) with Illumina reads.

A similar strategy was used to assemble the genome of *M. a. burmanica* and of *M. schizocarpa,* for which the whole Nanopore readset was used as input to the Necat assembler (github commit d377878). Alignment and assembly parameters were kept as default while the target genome size was set to 550Mb. The resulting assembly was polished 3 times with Racon (version 1.5.0) with Nanopore reads and 2 times with Hapo-G (version 1.3) with Illumina reads.

Regarding *M. a. zebrina* and *M. textilis*, we generated three samples of reads: one containing the complete Nanopore readset, one containing 30X of the longest reads and one corresponding to the highest-scoring Filtlong (version 0.2.0) reads. For the *M. a. zebrina* genome, the three subsets were inputted into Flye (v2.6), SMARTdenovo (commit 5cc1356), and Redbean (v2.5) assemblers. In the case of textilis, the inputs were processed using Flye (v2.5), Raven (v0.0.1), SMARTdenovo (commit 5cc1356), and Redbean (v2.5).

Necat (github commit d377878) was also launched on the whole Nanopore readset for zebrina. In both cases, SMARTdenovo was launched with '-k 17' and '-c 1', Redbean with '-xont -X5000 -g500m' and Flye, Raven and Necat were launched with default parameters. The best assembly was selected based on the contiguity of the assembly and polished 3 times with Racon (version 1.4.21 for zebrina and 1.4.7 for textilis) and Nanopore reads and 2 times using Illumina reads with Pilon (version 1.23) for *M. textilis* or Hapo-G (version 1.1) for *M. a. zebrina*.

Pisang Madu was the only genome sequenced using the Pacific Biosciences HiFI technology and assembled using Hifiasm (version r468) with default parameters.

Chromosome sequences reconstruction

M. a. banksii hybrid scaffolding: The BspQI and DLE-1 preparations were run on a single flow cell each. The generated molecules were assembled to produce optical maps thanks to software provided by Bionano Genomics (BNG) with the following two options: "add pre-assembly" and "non haplotype without extend and split" (bionano solve and tools Version: 3.3_10252018). The two optical maps and the ONT contigs were subjected to the 2 enzymes hybrid scaffolding pipeline to generate the hybrid scaffolds. BisCoT[15] was used to correct artefactual duplications (negative gaps) introduced during the scaffolding process. A final step of polishing was performed using Pilon and Illumina sequencing data. The assembly consisted of 149 hybrid scaffolds with a cumulative size of 485,32 Mb (Supplementary Data 3).

M. a. zebrina, *M. textilis* and *M. a. burmannica* Hi-C scaffolding: The ONT contigs were organized thanks to Hi-C scaffolding. Mapping of the Hi-C reads on the assembly was done thanks to Juicer tool with -s none parameter (https://github.com/aidenlab/juicer, version 1.6). The scaffolding was performed using 3D-DNA with -r 0 parameter (https://github.com/aidenlab/3d-dna, Software version: 180922). The Hi-C map was edited using Juicebox (https://github.com/aidenlab/Juicebox, version 1.11) and the scaffolding was manually corrected. The 406 *M. a. zebrina* contigs were scaffolded in 90 scaffolds with a N50 of 49.2Mb and a cumulative size of 551.5Mb. The 413 *M. a. burmannica* contigs were scaffolded in 66 scaffolds with a N50 of 44.1Mb and a cumulative size of 505.4Mb. The 838 M. textilis nanopore contigs were scaffolded in 104 scaffolds with a N50 of 50.9Mb and a cumulative size of 551.7Mb (Supplementary Data 3).

M. schizocarpa v2 hybrid scaffolding: Synteny analysis of the *M. schizocarpa* genome assembly[9] against *M. a. malaccensis* reference assembly (DH-Pahang v4[10]) revealed two large gene rich regions missing in the *M. schizocarpa* chromosomes 6 and 9 (Supplementary Fig. 37). Coverage analysis of Illumina read sequences (ERR10695626) from the same *M. schizocarpa* accession along the *M. a. malaccensis* reference assembly revealed that the *M. schizocarpa* accession used for the assembly was aneuploid in these missing regions with only one haplotype having them. The BspQI and DLE-1 preparations were run on a single flow cell each. The generated molecules were assembled to produce optical maps thanks to software provided by Bionano Genomics (BNG) with the following two options: "add pre-assembly" and "non haplotype without extend and split" (Bionano Solve Pipeline version 3.1.1)[9]. The two optical maps and the ONT contigs were subjected to the 2 enzymes hybrid scaffolding pipeline to generate the hybrid scaffolds (bionano solve and tools Version: 3.6.1_1116202). A new assembly using the same data as in Belser *et al*.[9] was performed with NECAT[16] but the two regions were still missing from the newly assembled version. The missing region of chromosome 6 was present in the ONT contigs and aligned on the optical maps but not scaffolded by the BNG software. The corresponding contig was manually added by taking into account the mapping results of the scaffolding process. In contrast, the missing region of chromosome 9 was absent in the ONT contigs. The ONT reads were mapped using minimap2 on the corresponding region of *M. a. malaccensis* reference assembly (DH-Pahang v4[10]). The aligned reads were assembled using NECAT (github commit d377878). The obtained contig was polished 3 times with Racon (version 1.4.7) and Nanopore reads and 2 times using Illumina reads with Hapo-G (version 1.1). This contig should be introduced in another contig which has been split. The hybrid scaffolding of chromosome 9 was redone with the new contig, the split contig and all the contigs previously placed on the chromosome 9. BisCoT[15] was used to correct artifactual duplications (negative gaps) introduced during the scaffolding processes. A final step of polishing was performed using Pilon and Illumina sequencing data. The assembly consisted of 213 hybrid scaffolds with a cumulative size of 534,64 Mb (Supplementary Data 3).

Assembly validation and genetic map anchoring

In order to obtain a quality score used to compare the different versions of the assembly, we downloaded and used merqury[17] version 1.3 (git commit 6b5405e). We used meryl[18] version 1.3 (git commit 3400615) to compute the Illumina reads k-mer counts via the meryl count command with a k-mer size of 21 and every other parameter set as default. Merqury was then launched on each set of k-mer counts (Supplementary Data 4).

Segregating populations (raw genotyping data available under projects PRJNA667853 and PRJNA1078411) used for anchoring the *M. a. zebrina, M. a. burmannica, M. a. banksii* and Pisang Madu assemblies (Supplementary Data 5), were described in Martin *et al.* [13]. In addition, a *M. textilis* self-progeny was developed at the CIRAD banana breeding platform in Guadeloupe. Segregating populations were genotyped-by-sequencing (GBS), the resulting raw reads from each population were aligned along corresponding assemblies (Supplementary Data 4). Segregating markers were obtained and selected and used to calculate simple matching pairwise dissimilarities (corresponding to the observed recombination frequency between pairs of markers) and to generate dot-plots showing marker genetic linkage as described in Martin *et al*.[13]. Dot-plot inspection allowed validation of the contiguity of assemblies. The linkage between contigs/scaffolds was used to anchor them into chromosomes.

As no genetic map was available for *M. schizocarpa,* scaffolds were then ordered into chromosomes based on synteny with *M. a. malaccensis* reference assembly (DH-Pahang v4[10]) as described in Belser *et al*.[9].

For scaffolds that were not anchored into chromosomes, an additional procedure was applied to classify them. The chloroplastic scaffolds were discarded by similarity to the chloroplast genome of DH-Pahang[19]. Mitochondrial derived scaffolds were selected based on BLAST similarity (Evalue 1.10^{-20}) to the 40 mitochondrial genes of the DH-Pahang Mitochondrial assembly[10]. If sequencing coverage of the scaffold was above the median coverage threshold, it was included into the "putative_mitochondrion" sequence. Remaining scaffolds were compared to the assembly with BLAST, BLASTn hits were merged using bedtools[20]. Scaffolds with less than 99% of sequence already present in the assembly were grouped in a chrUn-random synthetic sequence. Other scaffolds which were present in the assembly with more than 99% coverage were grouped in RepeatedInAssembly synthetic sequence. Thus, each assembly is provided with chromosomal sequences, a putative mitochondrion sequence containing mitochondrial scaffolds, a "chrUn-random" synthetic sequence with scaffolds that have not been anchored to chromosome sequence, and a "RepeatedInAssembly" synthetic sequence with scaffolds that are already present in chromosomes (Supplementary Data 4).

Gene prediction

Repeats in the genome assembly were masked using Tandem Repeat Finder[21] for tandem repeats and RepeatMasker[22] for simple repeats as well as known repeats included in RepBase[23]. In addition, known *Musa* transposable elements (from D'Hont *et al.*[24]), were detected using RepeatMasker.

Gene prediction was done using proteomes from homologous species, *Musa acuminata* (UP000012960), *Oryza. sativa* (GCF_001433935.1), *Zingiber officinale* (GCF_018446385.1) and *Musa balbisiana* (GCA_004837865.1).

The proteomes were aligned against the genome assembly in two steps. Firstly, BLAT[25] (default parameters) was used to quickly localize corresponding putative genes of the proteins on the genome. The best match and matches with a score ≥ 90% of the best match score were retained. Secondly, the alignments were refined using Genewise[26] (default parameters), which is more precise for intron/exon boundary detection. Alignments were kept if more than 50% of the length of the protein was aligned to the genome.

In order to predict expressed genes, several public RNAseq data were used according to their respective species as described in Supplementary Data 6. Reads were aligned on genome assembly using HiSat2 (v2.2.1, default parameters). Then, the output sam file was sorted using samtools (v1.15.1). The resulting BAM file was given to StringTie (v2.2.1, default parameters + -m 150 –rf) to generate transcript models. For each locus, the model with the highest TPM is kept only.

To proceed to the gene prediction, we integrated the protein homologies and transcript mapping using a combiner called Gmove[27]. This tool can find CDSs based on genome located evidence without any calibration step. Briefly, putative exons and introns, extracted from the alignments, were used to build a simplified graph by removing redundancies. Then, Gmove extracted all paths from the graph and searched for open reading frames (ORFs) consistent with the protein evidence. Finally, genes with CDS size less than 150 nt, or with CDS size less than 300 nt and a coding ratio (CDS size / transcript size) less 0.66, are removed. Completeness of the predicted genes was assessed with BUSCO[28] version 5.4.3 (embryophyta dataset odb10).

Supplementary Method 2. Pisang Madu haplotype parsing

The biparental mapping population Magda[13] (raw sequencing data available under project PRJNA667853) in which Pisang Madu is one parent was aligned onto *M. a. malaccensis* reference assembly (DH-Pahang v4[10]) in order to identify, select and phase segregating markers following the procedure described in Martin *et al.*[13]. The phased markers allowed access to Pisang Madu haplotypes. In parallel, sequences from two parents-child trios (DYN304 x DYN097 = MG3; DYN304 x DYN310 = PP2) implicating Pisang Madu as parent (the vcf from Martin *et al.*[6], raw sequencing data available under project PRJEB58004), were used to access Pisang Madu pseudo-haplotypes. These are not true haplotypes because recombination events occurred between the parents and the children. Phasing data from the population, which has been genotyped using GBS approach, does not allow for dense haplotyping whereas pseudo-haplotype data from parent-child trios, obtained from WGS data, allows for dense haplotype genotyping. These two datasets were thus combined, with phasing data from the population used as backbone to identify recombination in pseudo-haplotypes from each trio and correct these pseudo-haplotypes into the real haplotypes. These real haplotypes from each trio were then merged in order to increase the number of phased sites. Final result is a set of SNP positions along *M. a. malaccensis* reference sequence in which alleles were attributed to Pisang Madu haplotypes. Pisang Madu Illumina reads (from Martin *et al.*[6]) were aligned along the *M. a. malaccensis* reference sequence using vcfhunter tool box (https://github.com/SouthGreenPlatform/VcfHunter) and reads were then parsed according to the presence of alleles attributed to haplotypes.

At this stage a pool of reads per chromosome and per haplotype of Pisang Madu was obtained. However, the haplotype correspondence between chromosomes is not established (haplotype transmitted from parents of Pisang Madu). Pisang Madu is a hybrid with contributions of distinct genetic pools including the M_1 (=halabanensis) and M_2 ancestors[6]. The halabanensis origin, present along the full length of all chromosomes, was hypothesized to originate from the last hybridization event (one parent of Pisang Madu is representative of *M. a. halabanensis*). Thus, the idea is to identify per chromosome, the pool of reads corresponding to the haplotype of halabanensis origin that represents one of the Pisang Madu haplotypes. To do so, each read pool was aligned along the *M. a. malaccensis* reference sequence in order to perform a chromosome painting using methodology and ancestry specific alleles from Martin *et al.*[6] (Supplementary Fig. 42). The 11 read pools (1 per *M. a. malaccensis* reference sequence chromosome) that painted chromosomes of

halabanensis origin were assigned to Pisang Madu haplotype 1 and the 11 remaining read pools were assigned to Pisang Madu haplotype 2. These assigned read pools were then merged according to their origin (haplotype 1 or 2).

Merged read pools (from haplotype 1 and from haplotype 2) were then aligned against each contig obtained from Pisang Madu haplotype assemblies using BWA[29] with mem algorithm. Multiple hits, secondary alignments as well as alignments with mismatches were discarded using a combination of samtools[30] and BASH functions. Then, the number of hits of each read pool was calculated on sliding windows of 1kb and a haplotype was attributed to the segment if more than 10 reads were perfectly aligned and more than 60% of reads were from one of the 2 pools. Contigs with more than 10 regions of 10 kb attributed and more than 80% of these regions attributed to one haplotype were associated to the corresponding haplotype (Supplementary Data 8). The 10kb regions attribution to haplotype 1 and 2 were visualized along attributed contigs to look for large chimeric contigs (Supplementary Fig. 43). The absence of a large fragment from one haplotype followed by a large fragment from the second haplotype suggested that there are no large chimeric contigs. The relatively small regions of one haplotype into a background of the other haplotype are probably the consequence of the design of reads from each haplotype on a reference sequence that is slightly divergent from the present assembled genome haplotypes, especially for repeat regions, rather than small chimeric regions.

Supplementary Method 3. Chromosome ancestry painting of assemblies

We developed a methodology to "paint" chromosomes of assemblies according to ancestral genetic pools. The method consisted in (i) generating tags specific of each ancestral origin, (ii) aligning these tags against an assembly and (iii) attribute an ancestral origin along chromosomes using a sliding window.

Tags were generated using two distinct approaches. The first approach takes advantage of the work from Martin *et al.*[6] in which alleles specific to ancestral contributors to bananas were determined along *M. a. malaccensis* reference assembly (DH-Pahang v4[10]). Reads containing these ancestral alleles were parsed into different files (one per ancestral origin) after mapping reads from accessions from which these ancestral alleles were derived (Supplementary Data 9) along *M. a. malaccensis* reference assembly using vcfhunter toolbox. The aligned reads were parsed using the ParseReadsOnHaplo.py script that was added to vcfhunter toolbox. However, the (peri-)centromeric regions are poorly represented. Therefore, a second approach was developed in order to generate tags that sample these regions. For this, we looked for k-mers in the repeat part of the genome using individuals that are homogenous for only one ancestral contributor in pericentromeric regions[6] (Supplementary Data 9). The assembled haplotypes of Pisang Madu were also used to generate *in silico* 2 additional reads sets that were used in this analysis. One Pisang Madu set represented the *M. a. halabanensis* origin and the second set, by subtraction with sets from other ancestry, represented the unknown ancestor[6]. A total of 10,000,000 Illumina reads from each of these accessions were splitted into 50-mer. The number of reads bearing each k-mer was counted. K-mers were selected if present in more than 9 reads for each read set. This statistically samples k-mers found at least in three regions of the genome. Ancestry specific k-mers were identified as being present in at least one accession of a group but absent from all accessions from other groups. Ancestry specific k-mers were then used to search for corresponding reads in the complete read dataset for each origin.

A total of 1,000,000 and 2,000,000 reads per ancestry for the first and second approach were subsampled respectively. This allowed homogenisation of the number of exact hits (see next paragraph) between ancestry and regions sampled between approaches (Supplementary Fig. 44).

Read sets were then aligned against assemblies using BWA, exact matches were counted on non-overlapping sliding windows of 100kb using samtools and homemade scripts. An origin was attributed to a region based on the following criteria: (i) a window is considered if more than 10 total exact matches are observed in the window and (ii) an origin is attributed only if it represents more than 60% of the total exact matches in the window. Windows were then coloured according to attributed origin along the assembly. The complete painting process was performed using the PaintAssembly.sh that was added to VCFhunter toolbox (https://github.com/SouthGreenPlatform/VcfHunter).

The same painting approach was applied to Cavendish assemblies but using as colours the Cavendish haplotypes obtained by combining the 1x gametes and the 2x gamete proposed by Martin *et al.*[5]. The 1x gamete specific alleles were obtained as described in Martin *et al.* by removing the Mchare complete genotype from Cavendish genotyping. Alleles specific of each of the 2x gamete (unrecombined and unreduced gamete from an accession belonging to the Mchare group of clones) were determined using a combination of parents-child trio (DYN454 x DYN010 = MC2; raw sequencing data available under project PRJNA667853) and biparental mapping population (raw sequencing data

available under project PRJNA1182927) following methodology similar to the one described for Pisang Madu in Supplementary Method 2. In the trio, the true parent of individual MC2 is the Chicame accession but as there is no WGS sequencing data on this accession we used the Akondro Mainty accession which is an accession belonging to the same clonal group (Mchare) as Chicame[31].

Supplementary references

1. Belser, C. *et al.* Telomere-to-telomere gapless chromosomes of banana using nanopore sequencing. *Commun. Biol.* **4**, 1–12 (2021).
2. Wang, Z. *et al.* Musa balbisiana genome reveals subgenome evolution and functional divergence. *Nat. Plants* **5**, 810–821 (2019).
3. Martin, G. *et al.* Chromosome reciprocal translocations have accompanied subspecies evolution in bananas. *Plant J.* **104**, 1698–1711 (2020).
4. Huang, H.-R. *et al.* Telomere-to-telomere haplotype-resolved reference genome reveals subgenome divergence and disease resistance in triploid Cavendish banana. *Hortic. Res.* **10**, uhad153 (2023).
5. Martin, G. *et al.* Shared pedigree relationships and transmission of unreduced gametes in cultivated banana. *Ann. Bot.* **131**, 1149-1161 (2023).
6. Martin, G. *et al.* Interspecific introgression patterns reveal the origins of worldwide cultivated bananas in New Guinea. *Plant J.* **113**, 802–818 (2023).
7. Li, X. *et al.* Origin and evolution of the triploid cultivated banana genome. *Nat. Genet.* **56**, 136-142 (2024).
8. Liu, X. *et al.* The phased telomere-to-telomere reference genome of *Musa acuminata*, a main contributor to banana cultivars. *Sci. Data* **10**, 631 (2023).
9. Belser, C. *et al.* Chromosome-scale assemblies of plant genomes using nanopore long reads and optical maps. *Nat. Plants* **4**, 879-887 (2018).
10. Belser, C. *et al.* Telomere-to-telomere gapless chromosomes of banana using nanopore sequencing. *Commun. Biol.* **4**, 1047 (2021).
11. Zhou, R. *et al.* High-quality genome assemblies for two Australimusa bananas (*Musa* spp.) and insights into regulatory mechanisms of superior fiber properties. *Plant Commun.* **5**, 100681 (2023).
12. Rouard, M. *et al.* Three new genome assemblies support a rapid radiation in *Musa acuminata* (wild banana). *Genome Biol. Evol.* **10**, 3129–3140 (2018).
13. Martin, G. *et al.* Chromosome reciprocal translocations have accompanied subspecies evolution in bananas. *Plant J.* **104**,1698-1711 (2020).
14. Alberti, A. *et al.* Viral to metazoan marine plankton nucleotide sequences from the Tara Oceans expedition. *Sci. Data* **4**, 170093 (2017).
15. Istace, B., Belser, C. & Aury, J.-M. BiSCoT: improving large eukaryotic genome assemblies with optical maps. *PeerJ* **8**, e10150 (2020).
16. Chen, Y. *et al.* Efficient assembly of nanopore reads via highly accurate and intact error correction. *Nat. Commun.* **12**, 60 (2021).
17. Rhie, A., Walenz, B. P., Koren, S. & Phillippy, A. M. Merqury: reference-free quality, completeness, and phasing assessment for genome assemblies. *Genome Biol.* **21**, 245 (2020).
18. Miller, J. R. *et al.* Aggressive assembly of pyrosequencing reads with mates. *Bioinformatics* **24**, 2818–2824 (2008).
19. Martin, G., Baurens, F.-C., Cardi, C., Aury, J.-M. & D'Hont, A. The complete chloroplast genome of banana (*Musa acuminata*, Zingiberales): insight into plastid monocotyledon evolution. *PLoS ONE* **8**, e67350 (2013).
20. Quinlan, A. R. & Hall, I. M. BEDTools: a flexible suite of utilities for comparing genomic features. *Bioinformatics* **26**, 841–842 (2010).
21. Benson, G. Tandem repeats finder: a program to analyze DNA sequences. *Nucleic Acids Res.* **27**, 573–580 (1999).
22. Smit, A., Hubley, R. & Green, P. RepeatMasker Open-3.0. (1996-2010) http://www.repeatmasker.org.
23. Bao, W., Kojima, K. K. & Kohany, O. Repbase Update, a database of repetitive elements in eukaryotic genomes. *Mob. DNA* **6**, 11 (2015).
24. D'Hont, A. *et al.* The banana (*Musa acuminata*) genome and the evolution of monocotyledonous plants. *Nature* **488**, 213–217 (2012).
25. Kent, W. J. BLAT—The BLAST-Like Alignment Tool. *Genome Res.* **12**, 656–664 (2002).
26. Birney, E., Clamp, M. & Durbin, R. GeneWise and Genomewise. *Genome Res.* **14**, 988–995 (2004).
27. Dubarry, M. *et al.* Gmove a tool for eukaryotic gene predictions using various evidences. (2016) https://f1000research.com/posters/5-681.
28. Waterhouse, R. M. *et al.* BUSCO Applications from quality assessments to gene prediction and phylogenomics. *Mol. Biol. Evol.* **35**, 543–548 (2018).

29. Li, H. & Durbin, R. Fast and accurate long-read alignment with Burrows–Wheeler transform. *Bioinformatics* **26**, 589–595 (2010).
30. Danecek, P. *et al.* Twelve years of SAMtools and BCFtools. *GigaScience* **10**, giab008 (2021).
31. Perrier, X. *et al.* East African diploid and triploid bananas: a genetic complex transported from South-East Asia. *Ann. Bot.* **123**, 19–36 (2019).
