# Supplementary material for: Unravelling genomic drivers of speciation in Musa through genome assemblies of wild banana ancestors
Source: Nat Commun. 2025 Jan 23;16:961. doi: 10.1038/s41467-025-56329-4 (PMC11754795; doi:10.1038/s41467-025-56329-4)
Supplement: Supplementary file 14 — Reporting Summary [file 41467_2025_56329_MOESM14_ESM.pdf]

nature portfolio

Corresponding author(s): Guillaume Martin
Last updated by author(s): 14/01/25



# Reporting Summary

Nature Portfolio wishes to improve the reproducibility of the work that we publish. This form provides structure for consistency and transparency in reporting. For further information on Nature Portfolio policies, see our Editorial Policies and the Editorial Policy Checklist.

## Statistics

For all statistical analyses, confirm that the following items are present in the figure legend, table legend, main text, or Methods section.

| n/a | Confirmed | |
|---|---|---|
| ☐ | ☒ | The exact sample size (*n*) for each experimental group/condition, given as a discrete number and unit of measurement |
| ☒ | ☐ | A statement on whether measurements were taken from distinct samples or whether the same sample was measured repeatedly |
| ☒ | ☐ | The statistical test(s) used AND whether they are one- or two-sided<br>*Only common tests should be described solely by name; describe more complex techniques in the Methods section.* |
| ☒ | ☐ | A description of all covariates tested |
| ☒ | ☐ | A description of any assumptions or corrections, such as tests of normality and adjustment for multiple comparisons |
| ☒ | ☐ | A full description of the statistical parameters including central tendency (e.g. means) or other basic estimates (e.g. regression coefficient) AND variation (e.g. standard deviation) or associated estimates of uncertainty (e.g. confidence intervals) |
| ☒ | ☐ | For null hypothesis testing, the test statistic (e.g. *F*, *t*, *r*) with confidence intervals, effect sizes, degrees of freedom and *P* value noted<br>*Give P values as exact values whenever suitable.* |
| ☒ | ☐ | For Bayesian analysis, information on the choice of priors and Markov chain Monte Carlo settings |
| ☒ | ☐ | For hierarchical and complex designs, identification of the appropriate level for tests and full reporting of outcomes |
| ☒ | ☐ | Estimates of effect sizes (e.g. Cohen's *d*, Pearson's *r*), indicating how they were calculated |

*Our web collection on statistics for biologists contains articles on many of the points above.*

## Software and code

Policy information about availability of computer code

| | |
|---|---|
| Data collection | *Provide a description of all commercial, open source and custom code used to collect the data in this study, specifying the version used OR state that no software was used.* |
| Data analysis | Sequencing data and chromosome assembly:<br>Necat, Flye, Raven, SMARTdenovo, Redbean, Racon, Pilon, Hapo-G, Hifiasm; Merqury<br>Genome synteny, phylogenetic analysis and divergence time<br>liftoff v1.6.3, jcvi v1.1.18 - MCscan tool, MAFFT v7.475, PHYMLv3.1, Clustal W, PAL2NAL, MEGAX<br>Chromosome ancestry painting of assemblies:<br>https://github.com/SouthGreenPlatform/VcfHunter (https://github.com/SouthGreenPlatform/VcfHunter)<br>Repeats in Musaceae Assemblies:<br>EDTA 1.9.5, RepeatMasker 4.1.1, uclust 1.2.22, usearch 11.0.66, LTR Harvest 1.0, MREPS 2.6, ncbi-blast/2.10.1, vcfhunter 2.1.0, bedtools 2.29.2<br>Repeat elements Figures:<br>https://github.com/institut-de-genomique/Pahang-associated-data |

For manuscripts utilizing custom algorithms or software that are central to the research but not yet described in published literature, software must be made available to editors and reviewers. We strongly encourage code deposition in a community repository (e.g. GitHub). See the Nature Portfolio guidelines for submitting code & software for further information.

# Data

Policy information about availability of data

All manuscripts must include a data availability statement. This statement should provide the following information, where applicable:

- Accession codes, unique identifiers, or web links for publicly available datasets
- A description of any restrictions on data availability
- For clinical datasets or third party data, please ensure that the statement adheres to our policy

The genome assemblies and gene and TE annotations are available at Banana Genome Hub (BGH)(Droc et al, 2022) http://banana-genome-hub.southgreen.fr. The Illumina, ONT, PACBIO HiFi, Bionano Genomics, Hi-C data, assemblies and annotations are available in the European Nucleotide Archive under the following project PRJEB72282. GBSS and ADH alignments, EDTA repeat database and LTR repeat database produced are available on the Banana Genome Hub (http://banana-genome-hub.southgreen.fr), in the download section and on public dataverse (https://doi.org/10.18167/DVN1/6TLMD3). Source Data are also available on public dataverse (https://doi.org/10.18167/DVN1/6TLMD3). The GBS data are available in the Short Read Archive under the following projects PRJNA1078411, PRJNA1182927 and PRJNA667853.

# Research involving human participants, their data, or biological material

Policy information about studies with human participants or human data. See also policy information about sex, gender (identity/presentation), and sexual orientation and race, ethnicity and racism.

| | |
|---|---|
| Reporting on sex and gender | *Use the terms sex (biological attribute) and gender (shaped by social and cultural circumstances) carefully in order to avoid confusing both terms. Indicate if findings apply to only one sex or gender; describe whether sex and gender were considered in study design; whether sex and/or gender was determined based on self-reporting or assigned and methods used.*<br>*Provide in the source data disaggregated sex and gender data, where this information has been collected, and if consent has been obtained for sharing of individual-level data; provide overall numbers in this Reporting Summary. Please state if this information has not been collected.*<br>*Report sex- and gender-based analyses where performed, justify reasons for lack of sex- and gender-based analysis.* |
| Reporting on race, ethnicity, or other socially relevant groupings | *Please specify the socially constructed or socially relevant categorization variable(s) used in your manuscript and explain why they were used. Please note that such variables should not be used as proxies for other socially constructed/relevant variables (for example, race or ethnicity should not be used as a proxy for socioeconomic status).*<br>*Provide clear definitions of the relevant terms used, how they were provided (by the participants/respondents, the researchers, or third parties), and the method(s) used to classify people into the different categories (e.g. self-report, census or administrative data, social media data, etc.)*<br>*Please provide details about how you controlled for confounding variables in your analyses.* |
| Population characteristics | *Describe the covariate-relevant population characteristics of the human research participants (e.g. age, genotypic information, past and current diagnosis and treatment categories). If you filled out the behavioural & social sciences study design questions and have nothing to add here, write "See above."* |
| Recruitment | *Describe how participants were recruited. Outline any potential self-selection bias or other biases that may be present and how these are likely to impact results.* |
| Ethics oversight | *Identify the organization(s) that approved the study protocol.* |

Note that full information on the approval of the study protocol must also be provided in the manuscript.

# Field-specific reporting

Please select the one below that is the best fit for your research. If you are not sure, read the appropriate sections before making your selection.

☐ Life sciences ☐ Behavioural & social sciences ☒ Ecological, evolutionary & environmental sciences

For a reference copy of the document with all sections, see nature.com/documents/nr-reporting-summary-flat.pdf

# Ecological, evolutionary & environmental sciences study design

All studies must disclose on these points even when the disclosure is negative.

| | |
|---|---|
| Study description | Construction of high standard genome assembly for most ancestral contibutors of cultivated banana in addition to the banana reference genome to study chromosomal evolution in Musa lineage and domestication. |
| Research sample | Wild and cultivated diploid banana plants corresponding to potential ancestors of cultivated varieties. One individual per genetic group. |
| Sampling strategy | Based on litterature. Selection of one individual per genetic group corresponding to the potential ancestors of cultivated varieties. |

| | |
|---|---|
| Data collection | Plants were obtained from the Bioversity International Musa Germplasm Transit Centre (ITC) and from CIRAD-INRAE Biological Resource Centre for Tropical Plants (CRB-PT) (see PLANT section). |
| Timing and spatial scale | Single date of collection |
| Data exclusions | no data excluded |
| Reproducibility | DNA was extracted from young leaves and cytogenetic was performed on young roots. All attempts to repeat the experiment were successful. |
| Randomization | no randomization |
| Blinding | no blinding |

Did the study involve field work? ☐ Yes ☒ No

# Reporting for specific materials, systems and methods

We require information from authors about some types of materials, experimental systems and methods used in many studies. Here, indicate whether each material, system or method listed is relevant to your study. If you are not sure if a list item applies to your research, read the appropriate section before selecting a response.

## Materials & experimental systems

| n/a | Involved in the study | |
|---|---|---|
| ☒ | ☐ | Antibodies |
| ☒ | ☐ | Eukaryotic cell lines |
| ☒ | ☐ | Palaeontology and archaeology |
| ☒ | ☐ | Animals and other organisms |
| ☒ | ☐ | Clinical data |
| ☒ | ☐ | Dual use research of concern |
| ☐ | ☒ | Plants |

## Methods

| n/a | Involved in the study | |
|---|---|---|
| ☒ | ☐ | ChIP-seq |
| ☒ | ☐ | Flow cytometry |
| ☒ | ☐ | MRI-based neuroimaging |

## Dual use research of concern

Policy information about dual use research of concern

### Hazards

Could the accidental, deliberate or reckless misuse of agents or technologies generated in the work, or the application of information presented in the manuscript, pose a threat to:

| No | Yes | |
|---|---|---|
| ☒ | ☐ | Public health |
| ☒ | ☐ | National security |
| ☒ | ☐ | Crops and/or livestock |
| ☒ | ☐ | Ecosystems |
| ☒ | ☐ | Any other significant area |

### Experiments of concern

Does the work involve any of these experiments of concern:

| No | Yes | |
|---|---|---|
| ☒ | ☐ | Demonstrate how to render a vaccine ineffective |
| ☒ | ☐ | Confer resistance to therapeutically useful antibiotics or antiviral agents |
| ☒ | ☐ | Enhance the virulence of a pathogen or render a nonpathogen virulent |
| ☒ | ☐ | Increase transmissibility of a pathogen |
| ☒ | ☐ | Alter the host range of a pathogen |
| ☒ | ☐ | Enable evasion of diagnostic/detection modalities |
| ☒ | ☐ | Enable the weaponization of a biological agent or toxin |
| ☒ | ☐ | Any other potentially harmful combination of experiments and agents |

## Plants

| | |
|---|---|
| Seed stocks | CIRAD-INRAE Biological Resource Centre for Tropical Plants (CRB-PT) in the West Indies (Guadeloupe, France) for accessions PT-BA-00024 (M. acuminata. ssp banksii); PT-BA-00228 for M. textilis; PT-BA-00182 for M. a. spp zebrina; PT-BA-00051 for M. a. spp burmannicoides and PT-BA-00304 for the diploid cultivated Pisang Madu. The Bioversity International Transit Center in Leuven (Belgium) for accession ITC926 of M. schizocarpa |
| Novel plant genotypes | *Describe the methods by which all novel plant genotypes were produced. This includes those generated by transgenic approaches, gene editing, chemical/radiation-based mutagenesis and hybridization. For transgenic lines, describe the transformation method, the number of independent lines analyzed and the generation upon which experiments were performed. For gene-edited lines, describe the editor used, the endogenous sequence targeted for editing, the targeting guide RNA sequence (if applicable) and how the editor was applied.* |
| Authentication | *Describe any authentication procedures for each seed stock used or novel genotype generated. Describe any experiments used to assess the effect of a mutation and, where applicable, how potential secondary effects (e.g. second site T-DNA insertions, mosiacism, off-target gene editing) were examined.* |
